# MISPR: an open-source package for high-throughput multiscale molecular simulations

**DOI:** 10.1038/s41598-022-20009-w

**Published:** 2022-09-21

**Authors:** Rasha Atwi, Matthew Bliss, Maxim Makeev, Nav Nidhi Rajput

**Affiliations:** Department of Materials Science and Chemical Engineering, Stony Brook University, Stony Brook, NY 11794 USA

**Keywords:** Atomistic models, Computational methods, Electronic structure, Fluids, Computational chemistry, Density functional theory, Molecular dynamics

## Abstract

Computational tools provide a unique opportunity to study and design optimal materials by enhancing our ability to comprehend the connections between their atomistic structure and functional properties. However, designing materials with tailored functionalities is complicated due to the necessity to integrate various computational-chemistry software (not necessarily compatible with one another), the heterogeneous nature of the generated data, and the need to explore vast chemical and parameter spaces. The latter is especially important to avoid bias in scattered data points-based models and derive statistical trends only accessible by systematic datasets. Here, we introduce a robust high-throughput multi-scale computational infrastructure coined MISPR (Materials Informatics for Structure–Property Relationships) that seamlessly integrates classical molecular dynamics (MD) simulations with density functional theory (DFT). By enabling high-performance data analytics and coupling between different methods and scales, MISPR addresses critical challenges arising from the needs of automated workflow management and data provenance recording. The major features of MISPR include automated DFT and MD simulations, error handling, derivation of molecular and ensemble properties, and creation of output databases that organize results from individual calculations to enable reproducibility and transparency. In this work, we describe fully automated DFT workflows implemented in MISPR to compute various properties such as nuclear magnetic resonance chemical shift, binding energy, bond dissociation energy, and redox potential with support for multiple methods such as electron transfer and proton-coupled electron transfer reactions. The infrastructure also enables the characterization of large-scale ensemble properties by providing MD workflows that calculate a wide range of structural and dynamical properties in liquid solutions. MISPR employs the methodologies of materials informatics to facilitate understanding and prediction of phenomenological structure–property relationships, which are crucial to designing novel optimal materials for numerous scientific applications and engineering technologies.

## Introduction

In the past decades, immense progress has been made in developing computational tools to predict material properties across multiple length and time scales^1^. These tools have significantly contributed to the understanding of complex physical/chemical processes and facilitated the discovery of novel materials in fields such as batteries^2–4^, photovoltaics^5^, catalysts^6,7^, and pharmaceuticals^8^. However, the search for advanced materials requires navigating multidimensional landscapes of enormous complexity. Given the intricate involvement of multiple computational chemistry software and the vast quantity of heterogeneous data generated from these simulations, it has become imperative to automate these calculations for screening materials within realistic time constraints. A key question is how to gain a predictive understanding of complex inter- and intra-atomic interactions in multi-component liquid solutions that control various electronic, thermodynamic, structural, and dynamical properties. In this respect, developments of software infrastructure beyond simple scripting for high-throughput computations have been on the rise within the materials science community, following the impetus from the experimental high-throughput approaches^9,10^. Some noteworthy examples include Polymer Genome^11^, Automatic Flow (AFLOW)^12^, atomic simulation recipes (ASR)^13^ and MyQueue task scheduler^14^, Novel Materials Discovery (NOMAD)^15^, the Materials Project^16^, the Harvard Clean Energy Project^17^, the Electronic Structure Project^18^, the Open Quantum Materials Database (OQMD)^19^, the Materials Simulation Toolkit (MAST)^20^, and AiiDA^21^. The common goals of many of these frameworks are to employ high-throughput computations in three connected steps: (1) calculation of thermodynamic and electronic properties of materials, (2) storage of materials information in database repositories, and (3) data analysis aimed at screening promising material candidates for a given application or gaining new physical insights. The data stored in these databases include crystal structures, nanoporous materials, bandgaps, formation energies, piezoelectric coefficients, elastic constants, and magnetic moments. These developments show immense promise for accelerating materials discovery and the excellent level of maturity of the computational high-throughput approach. However, similar approaches at both quantum and classical levels to predict molecular and ensemble properties in liquid solutions are still in their infancy. Integrating hierarchies of paradigms and scales is also crucial for building a meaningful understanding of various physical and chemical processes and obtaining insights beyond chemical intuition and, thus, is the primary driver for the development of MISPR (Materials Informatics for Structure–Property Relationships).

Implemented and operated through the Python language, MISPR is a high-level interface designed to automate complex hierarchical simulations through the definition of modular density functional theory (DFT) and classical molecular dynamics (MD) workflows. Our work intersects with the previously mentioned computational frameworks in several features, such as the use of built-in databases for storing calculation results and the addition of error handlers to automatically restart a calculation. However, what distinguishes MISPR from existing frameworks are its (1) flexibility in running multi-scale simulations in the same workflow, (2) integration of MD analysis tools with high-throughput workflows, (3) automatic cluster sampling from MD simulations to build explicit solvation environments crucial for obtaining accurate properties from DFT calculations^22–24^, and (4) support for workflow templates to compute molecular (e.g. bond dissociation energy, binding energy, and redox potentials, with unprecedented support for multiple methods) and ensemble (e.g., diffusivity, viscosity, ionic conductivity, residence time, radial distribution function, coordination number, solvation structure, and dielectric constant) properties in high-throughput mode.

MISPR stores the resulting heterogeneous data in a database in a way that is easily shareable, accessible, and reusable with different tools, following the FAIR data principles^25,26^. MISPR databases, constructed via the FireWorks^27^ library, record provenance in detail, by storing the interconnections between data and the chain of steps that produced it as output. They also include the input parameters and the metadata necessary to make them easily queryable. Other features of MISPR include inherited support for multiple queueing systems from FireWorks, automatic force field generation through AmberTools^28^, and flexibility to implement custom workflows.

This paper is organized as follows. First, we give an overview of the functionalities supported by MISPR, discuss its dependencies on the software stack developed by the Materials Project, and describe its architecture. We then highlight the automatic error handling and technical validation aspects when running simulations in high-throughput mode using MISPR. Finally, we provide an in-depth description of the implemented DFT and MD workflows, along with demonstrations of their outputs and the structure of the generated databases.

## Results and discussion

### Overview of the infrastructure

**Figure 1:**
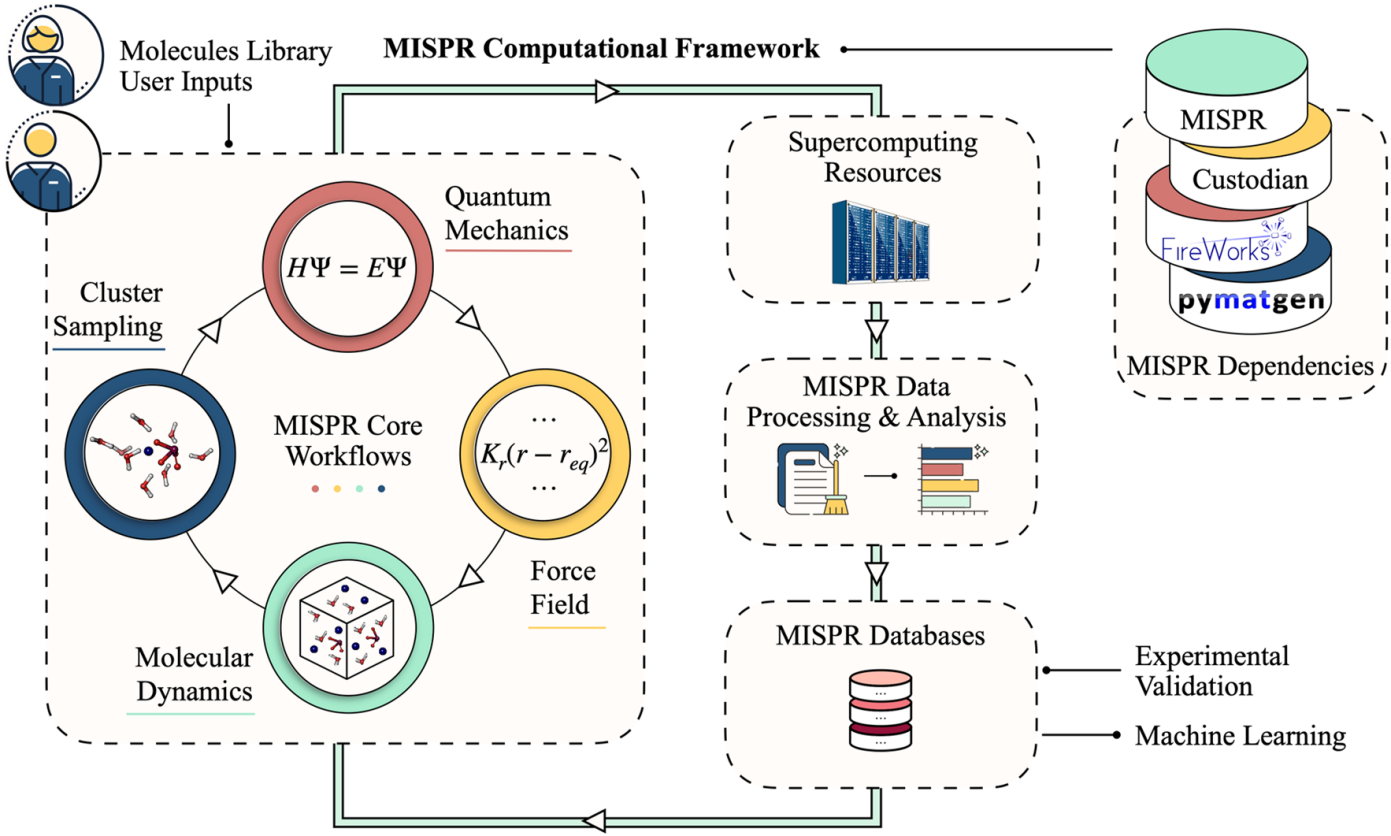
An overview of MISPR infrastructure. Users may choose to integrate several MISPR components to derive a specific materials property and design optimal or novel materials.

Figure 1 depicts an overview of the MISPR infrastructure along with its dependencies. It starts from a database of individual molecular species of interest to a particular materials science application. First, MISPR uses DFT calculations to optimize the geometry of these molecules, ensure that a true minimum is reached, and extract partial charges on atoms, which it then fits using the standard RESP protocol^29^. In the next step, the infrastructure uses the optimized geometries and partial charges to build an initial configuration of the multi-bodied system and obtain force field parameters. Then, it generates input files for the target simulation engine and runs it to relax the system to its equilibrium state. Finally, it produces trajectories representing the motion of atoms at specific time intervals. Force field parameters present a challenging aspect of such a framework, as they can be a frequent source of error in MD simulations. Therefore, MISPR supports multiple state-of-the-art force fields, allowing the user to rapidly and systematically assess the accuracy of physical models for a particular application or property. The infrastructure also utilizes MDPropTools, a standalone in-house suite of Python-based post-processing routines, to perform statistical analysis of thermodynamic, structural, and dynamical properties by averaging over user-selected number of independent trajectories.

In some cases, dielectric continuum models are inadequate for describing relatively strong interactions that may occur between solute and solvent molecules^30^. Combining discrete and continuum models is thus essential for an accurate evaluation of properties in solution, such as spectroscopic properties and redox potentials. Therefore, MISPR supports automatic sampling of representative solute–solvent configurations from MD workflows and feeding them to various DFT workflows.

MISPR uses the object-oriented programming (OOP) paradigm in the high-level programming language Python. Such an OOP design has numerous advantages. It facilitates code reuse and ensures modularity, making it easy to utilize existing classes to incorporate new workflows. In addition, it allows grouping together different calculation steps into a single entity, thus hiding as many details as possible, which allows the user to interact with the infrastructure at a relatively high level. MISPR comes with online documentation (https://molmd.github.io/mispr/) and distributed version control through GitHub. At the backend, it has support for Gaussian electronic structure software package^31^, which is widely used for DFT-based calculations, AmberTools for force field parameter generation^28^, and LAMMPS (http://lammps.sandia.gov) open-source package for classical MD simulations^32^. We note that although MISPR itself is open-source, some of the software packages that it interfaces with (e.g., Gaussian) are not and require a commercial license to use.

### Base libraries

Each of the techniques supported by MISPR (i.e., DFT and MD simulations) has its technical settings, program packages, data representations, and input/output file formats. In addition to handling this heterogeneity, running the calculations requires writing and submitting scripts to different queueing systems, monitoring the status of jobs, and dealing with potential errors during the computations. MISPR is developed with a central goal of automating these repetitive tasks via creating, managing, and analyzing scientific data and workflows. The infrastructure leverages several continuously growing base Python packages developed by the Materials Project. Specifically, (1) pymatgen^33^ is used for structure representation and handling input/output file formats, (2) FireWorks for managing and executing workflows on various job schedulers such as SGE, PBS, and SLURM and monitoring the status of calculations, and (3) custodian^33^ for automatic diagnosis of inevitable errors and job resubmission by applying rule-based fixes to failed runs. MISPR combines these three codebases to achieve the goals presented earlier. We extended custodian by implementing new plugins for applying recipe-like fixes to errors specific to Gaussian codes. In the infrastructure, we also took inspiration from other efforts by the Materials Project: (1) atomate^34^, which interfaces with multiple computational chemistry software focusing on VASP^35–37^-based workflows, and (2) Electrolyte Genome^38^, which was developed for predicting molecular properties of organic liquid-electrolyte materials via ab initio calculations in Q-Chem code^39^.

### Software architecture

The DFT and MD sub-packages represent the core of MISPR, each consisting of five principal pillars: (1) high-level scientific workflow abstractions, (2) low-level classes and functionalities that can be reused to compute similar properties in different contexts, (3) tools for data processing, storing, and analytics, (4) utility modules, and (5) error-correcting plugins.

A scientific workflow provides a complete description of the procedure leading to the final data used to predict the desired property. It consists of multiple steps ranging from the initial setup of a molecule or system of molecules to a sequence of calculations with dependencies and optional automated post-processing of parsed data to derive properties of interest. The workflow model we use to encode DFT and MD recipes in MISPR is based on the classes and structure of the FireWorks workflow library. A workflow in FireWorks is modeled as a Directed Acyclic Graph representing the chain of relationships between computational operations. A workflow consists of one or more Fireworks (jobs) with dependencies. Examples of Fireworks implemented in MISPR include running a DFT geometry optimization and equilibrating a multi-bodied system via MD simulations. The workflow contains information about the links between Fireworks to execute them in the correct order. Each Firework consists of one or more Firetasks that run sequentially. A Firetask is an atomic computing job that can call shell scripts, transfer files, write/delete files, or execute other Python functions^27^. For instance, Firetasks  in MISPR can write input files for Gaussian and LAMMPS software, parse output files, perform analysis to derive final properties, and insert results into the database.

The DFT and MD sub-packages of MISPR are organized according to these three levels (Firetasks, Fireworks, workflows). The user can tune the calculations at the workflow level by specifying input parameters (e.g., the level of theory and solvent model for DFT calculations and the ensemble, temperature, and timestep for MD simulations). Many of the input parameters to the workflows are optional. The user can run an entire calculation with minimal input, and default values will be used if these parameters are not provided. In addition, the user can build custom workflows by assembling existing Firetasks and Fireworks. The user has the flexibility to skip specific steps of a workflow (e.g., optimization and frequency of a structure in DFT calculations), perform seamless restarts, and modify a predefined workflow to include additional tasks. In the last step of the analysis Firetask of some workflows, the infrastructure invokes specific Matplotlib^40^ functions for data visualization.

The DFT and MD sub-packages of MISPR also include utility modules containing Python functions for frequently repeated tasks. These tasks range from handling various structure representations via Open Babel^41^ and pymatgen libraries, generating metadata for data storage, modifying predefined workflows to achieve a specific desired behavior, among many others.

Multiple input, intermediate, output, and error files are generated while running DFT and MD simulations in high-throughput mode. Therefore, besides database storage, it is crucial to have a parallel file management system. To this end, the infrastructure organizes files resulting from a workflow in a pre-determined directory structure, as explained in Sect. 1 of the SI. Output files generated by workflows are parsed and converted to a JSON-style document with the necessary metadata. Users can insert this document into the database or write it to disk as a JSON file in its corresponding subfolder.

### Error handling

Automatic error recovery is an essential aspect of high-throughput simulations. Therefore, we implement an error-correcting framework specific to Gaussian through the custodian library and using the concept of dynamic workflows. A parallel LAMMPS plugin is under development. The infrastructure gives the flexibility of running workflows independent of the custodian library.

In Table 1, we provide a list of some of the commonly encountered errors in DFT calculations and their fixing methods as implemented in MISPR. When an error is encountered, the error-handler applies an appropriate fix, generally by modifying the input parameters (e.g., the number of SCF cycles in DFT calculations) and restarting the calculation. Because the same calculation is prone to fail again, the infrastructure applies the same recipe-based error correction procedure to the task. However, non-trivial situations in which the infrastructure cannot interpret the error or find an appropriate remedy are possible, in which case the calculation is allowed to fail. Furthermore, errors not accounted for so far can be quickly added to the plugins. Hence, we continue to develop these error handlers.

**Table 1:** Examples of DFT (Gaussian) calculations fixing methods as implemented in MISPR.

Error	Remedy
Linear bend angle/torsion	Regenerate a new set of internal coordinates and restart the calculations
SCF convergence	(1) Increase the number of SCF cycles (2) Use an alternate SCF algorithm like xqc (3) Generate a better initial guess at a lower level of theory and use it as input for the calculation at the original level of theory
Optimization failure	(1) Increase the number of optimization cycles, set the last geometry as the new starting geometry, and rerun (2) Restart the job from the last geometry if the job appears to be converging (*i.e.*, convergence criteria have values lower than the starting point) (3) Use an ultrafine integration grid for numerical integration (4) Generate a better initial guess for the geometry using a lower level of theory and use it to initiate calculations at the original level of theory
PCM iteration convergence	Change the type of molecular surface representing the solute–solvent boundary and restart the calculations
Internal coordinate failure	For optimization jobs, switch to Cartesian coordinates while overwriting other explicitly set coordinate systems and disable symmetry when applicable; this remedy comes at an additional computational cost
Exceeded wall time	Cancel the running job just before it hits the allocated wall time and restart the job by submitting a new job script

### Basic operation

The user can call a workflow and provide inputs as minimal as the initial structure(s) and carry an entire set of predefined calculations. The user can override default parameters at the workflow, Firework, and Firetask levels. This is done by creating a Python script, in which the user calls existing DFT and MD workflow functions and provides their corresponding inputs. Figure S1 shows an example of a snippet code that demonstrates how to submit a bond dissociation energy (BDE) calculation for a monoglyme molecule defined in XYZ file format. The user submits the jobs to the FireWorks databases, from which jobs can then be queried for execution at distributed resources. The FireWorks database is updated with runtime provenance, allowing the user to search for and further examine the jobs and their states, which are marked as RUNNING, FIZZLED (failed), etc.^27^.

### Technical validation

By automatically documenting data transformations during a workflow with all inputs consumed and the codes run by the process, the results predicted by MISPR are, in principle, fully reproducible. Versions of the infrastructure, the relevant plugins, and the source code of the external simulation software are stored as metadata in the database for each calculation. Besides enabling reproducibility, tracking data transformation can serve as a "caching" mechanism that saves computational resources by skipping a calculation if a similar one has been previously completed with identical inputs.

MISPR also enables systematic exploration of different simulation parameters, e.g., the effect of density functional and basis set in DFT calculations and force field parameters and box size in MD simulations. The default parameters are set based on extensive benchmarking to balance the computational cost and accuracy for DFT and MD simulations. Still, the user can tune these inputs based on tests for a benchmark set in a specific application area. In addition, situations in which a calculation succeeds but leads to physically meaningless results are not uncommon and can lead to outliers in final results. Therefore, the infrastructure sets checkpoints in the workflows to identify these occurrences. For instance, whenever a geometry optimization is performed, it is followed by a frequency calculation to guarantee a true minimum is reached. Fluctuations in MD properties like temperature and pressure are reported to ensure a stable system. If a checkpoint is not met, dynamic changes (e.g., perturbing the geometry of a molecule in DFT calculations and randomizing the initial configuration of molecules in MD simulations) are made to the workflows during its execution.

### Workflows

**Figure 2:**
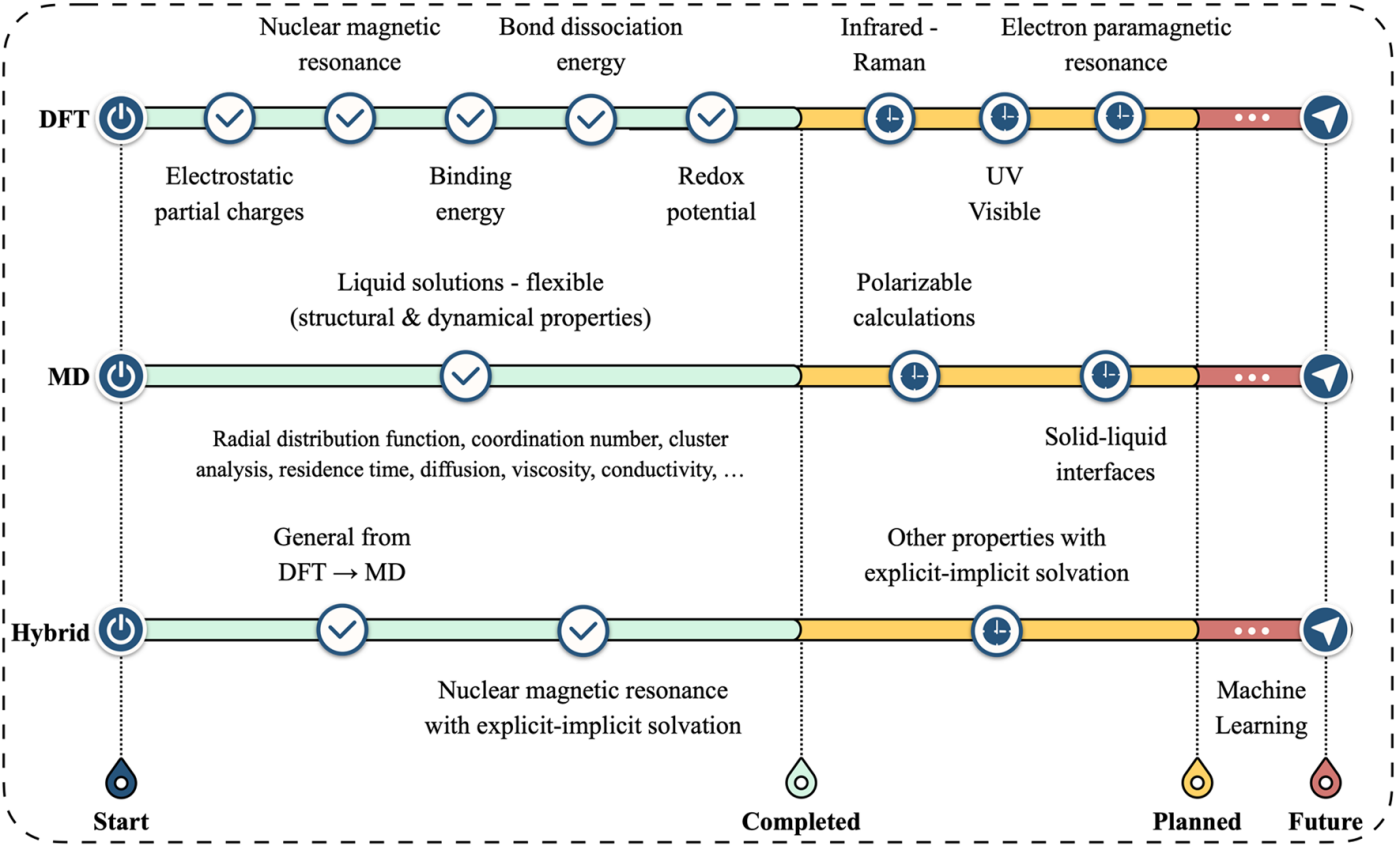
Roadmap of existing and planned workflows in MISPR.

This section describes DFT and MD workflows implemented in MISPR. A summary roadmap of existing and planned workflows is shown in Fig. 2.

#### Electrostatic partial charges

**Figure 3:**
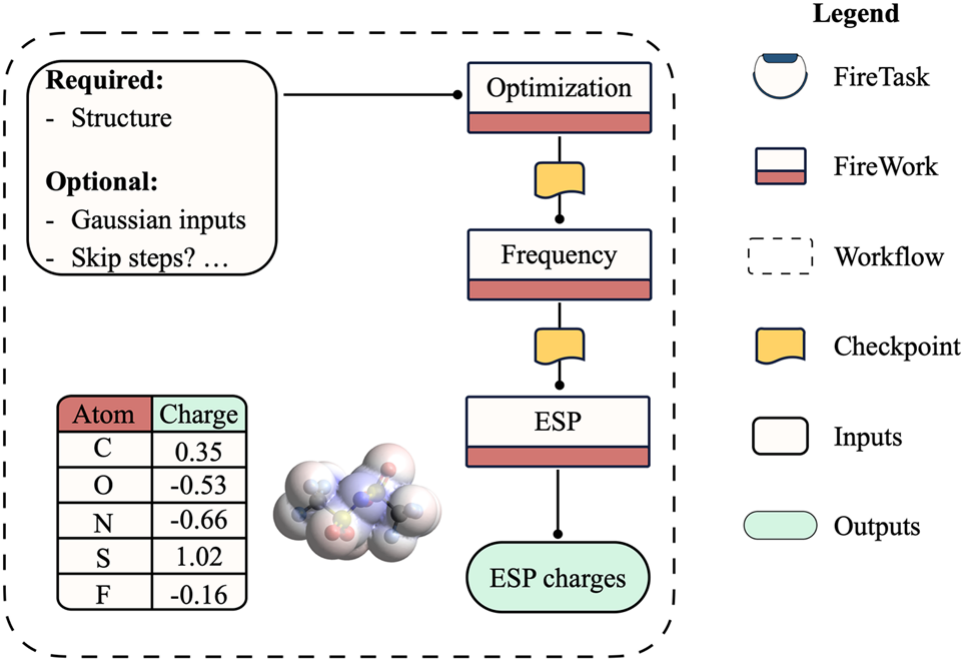
Workflow diagram for the electrostatic partial charge calculation.

To describe columbic interactions in dynamic environments, we implement a workflow (Fig. 3) that calculates electrostatic partial charges (ESP) that can be fit using the popular RESP method. By default, this workflow produces charges fit to the electrostatic potential at points selected according to the Merz–Singh–Kollman scheme^42,43^, but any other scheme supported by Gaussian can be used. The ESP workflow starts by processing information about the initial molecular structure provided by the user. This information can be a pymatgen molecule object, a previously calculated molecule stored in the database, a Gaussian output file or dictionary, or any other format handled by OpenBabel^41^ and pymatgen. The workflow also supports the on-spot derivation of molecules by either attaching functional groups or connecting two molecules at a binding site. The structure processing step can be done recursively to, for example*,* attach more than one functional group or link more than two structures simultaneously, making structures more readily accessible. This step also comes with an optional local optimization procedure using a force field calculation to feed a better initial guess to the next DFT job. The workflow proceeds with a structure optimization, whose output is passed to a Firework representing a frequency calculation to ensure a true minimum is reached. Following this, an ESP job is performed in a separate Firework, and predicted ESP charges are inserted into an ESP collection in the database and output to a file that can be later used for deriving RESP charges. The user can tune the standard ESP workflow in MISPR as needed. For example, the optimization and frequency steps can be bypassed when a relaxed structure is available. Bulk contributions can be approximated by providing the workflow with an implicit solvation model along with solvent properties. The user can override preset components of the level of theory like the density functional and basis set, along with other DFT parameters like convergence parameters for the SCF procedure and geometry optimization and numerical and algorithmic parameters. Many of the features described here are also common with other DFT workflows in MISPR. Sample ESP charges calculated using MISPR for a monoglyme molecule are shown in Table S1 and an extended dataset of ESP charges for 500 molecules is available in the data GitHub repository associated with this manuscript.

#### Nuclear magnetic resonance

**Figure 4:**
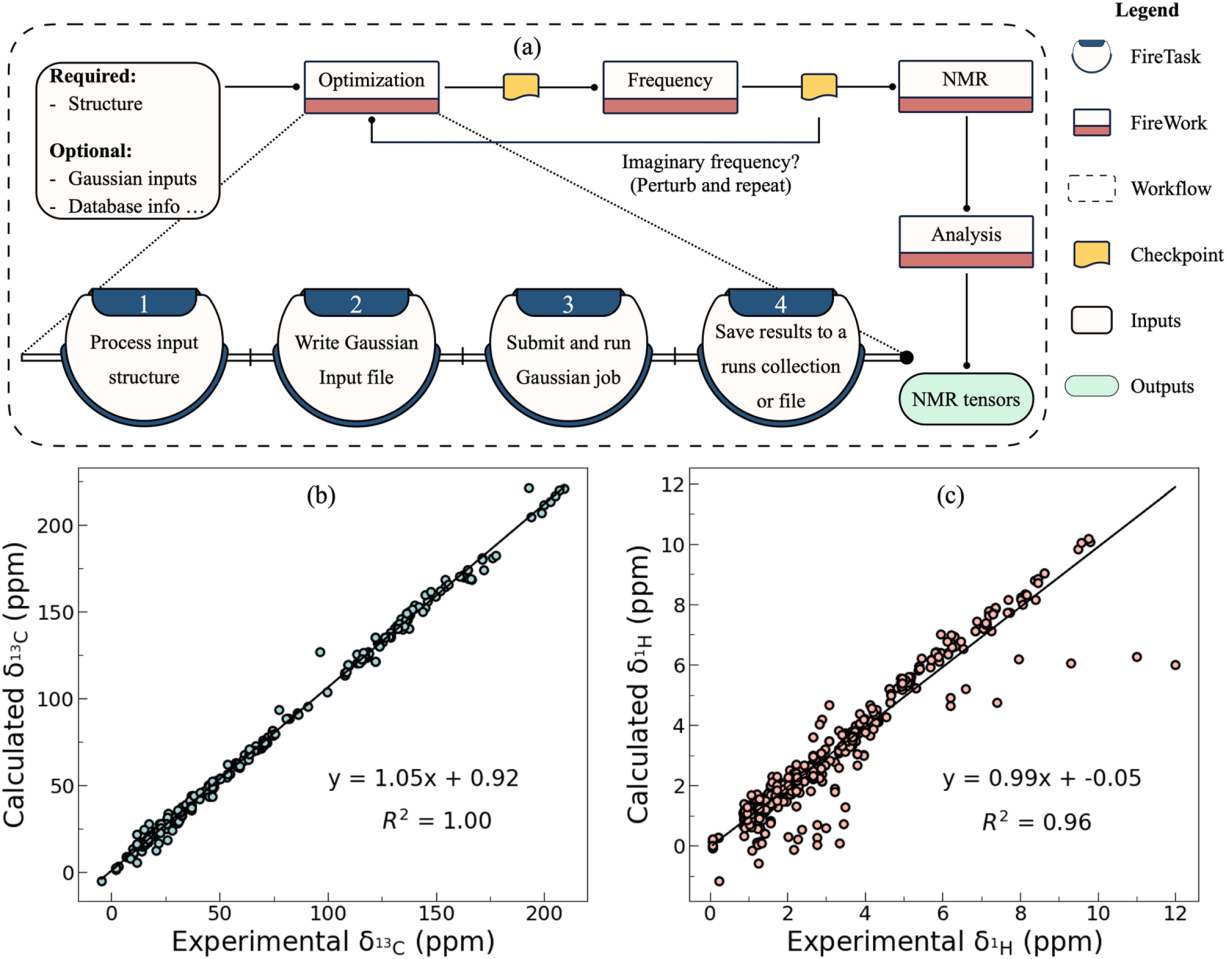
NMR workflow (**a**) diagram of the standard steps of the workflow that only requires the structure as input but the user can customize parameters and operations as needed, and linear regression of sample (**b**) ^13^C and (**c**) ^1^H chemical shifts obtained from the MISPR workflow versus experimental NMR chemical shifts. R^2^ indicates the correlation coefficient.

Nuclear magnetic resonance (NMR) spectroscopy is a powerful tool for characterizing materials and identifying reaction intermediates^44^. Therefore, we implement an NMR workflow in MISPR (Fig. 4a). The structure of this workflow closely mirrors that of the ESP workflow. Calculations begin with a structure optimization followed by a frequency job. The optimized structure is then passed to an NMR Firework, which produces by default shielding tensors on each atom. Users may request to compute magnetic susceptibility and spin–spin coupling constants by modifying the corresponding Gaussian input parameters. The preset settings use the Gauge-Independent Atomic Orbitals (GIAO)^45–49^ method, but this can be overridden by other Gaussian-supported methods like CSGT^49–51^ and IGAIM^50,51^. NMR tensors are then stored in the NMR collection of the database or written to a local JSON file. This output can be used to calculate the chemical shift of a specific nucleus by simply subtracting it from the output of a parallel calculation using a reference compound. The workflow can be applied not only for small molecules in the gas phase but also for structures of varying sizes and chemistries in solution. Users may choose to append this workflow to the MD workflow in MISPR to seamlessly sample several thousands of solvation structures and calculate their NMR properties in high-throughput mode. The benefits of this procedure are multifold. First, it improves accuracy by reliably accounting for the underlying environmental effects on the atoms/molecules of interest by mixing discrete and continuous solvent models. Second, it eliminates trial and error associated with building possible solvation structures based on chemical intuition, particularly in complex multi-component solutions or systems that have never been probed before. In addition, it accounts for multiple solvation structures representing different numbers and types of molecules correctly placed in the first solvation shell of the particle of interest, a step that is especially important in dynamic solutions. We applied this procedure to elucidate the solvation structure of a complex multi-component solution of magnesium bis(trifluoromethylsulfonyl)imide salt dissolved in monoglyme solvent and found excellent agreement with experimentally measured ^25^Mg, ^13^C, and ^1^H NMR spectra^52^.

Figure 4b,c show sample parity plots of ^13^C and ^1^H chemical shifts automatically calculated using this workflow for a library of 100 organic compounds consisting of 351 ^13^C and 685 ^1^H chemical shifts. The workflow automatically generated and processed more than 600 input and output files, and the calculations took less than 12 h of wall-clock time for the entire library. A positive correlation is found between the predicted ^13^C and ^1^H chemical shifts and the experimental measurements with only minor deviations from the fitted line. The error distributions are shown in Fig. S2.

#### Bond dissociation energy

**Figure 5:**
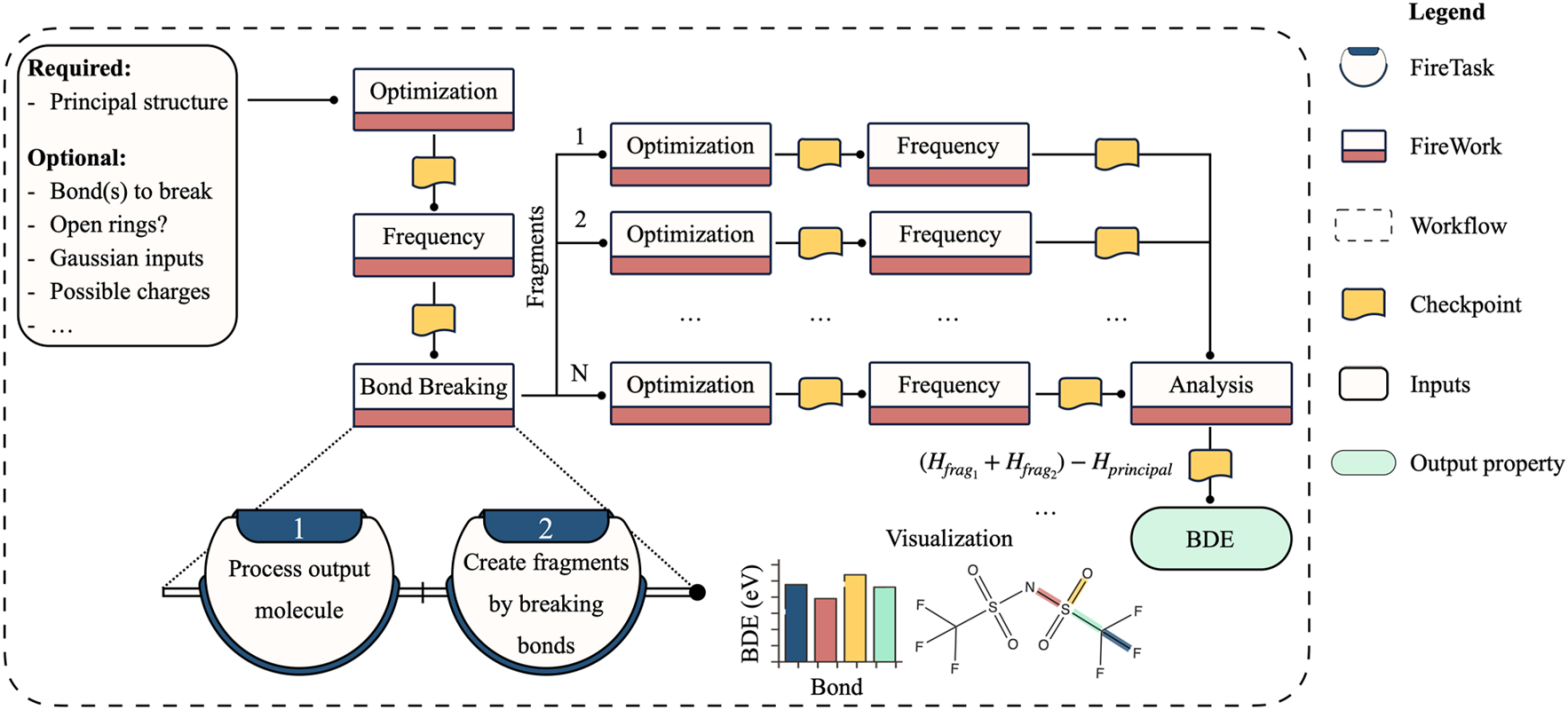
Workflow diagram for the bond dissociation energy calculation.

BDE is a thermodynamics metric used widely to determine the stability of chemical compounds and their chemical reactivity and selectivity^53,54^. It is typically the first step in characterizing dominant reaction pathways in biofuel combustion^55^, drug metabolism^56^, plastic polymers decomposition^57^, electrolytes in electrosynthesis^58^ and energy storage devices^59^. However, BDE calculations require substantial effort, in which the reactant and all its possible products need to be optimized, and their Hessian be evaluated. This process becomes impractical when a relatively large chemical space is explored. Therefore, MISPR includes a BDE workflow that requires the structure of the reactant as input at a minimum. A visual depiction of this workflow is shown in Fig. 5. The workflow subjects the reactant to the typical relaxation and vibrational frequency Fireworks. The frequency Firework outputs thermodynamic quantities including energy, enthalpy, entropy, and Gibb free energy in JSON format. Next, the relaxed structure is passed to a *BreakMolecule* Firework that, by default, attempts to break all non-cyclic bond(s) in the molecular graph representation of the reactant, forming two products. This fragmentation process is performed via the *fragmenter* module in the pymatgen package. Users may also choose which bonds to break by providing their corresponding site indexes. The *BreakMolecule* Firework generates a new sequence of optimization and frequency Fireworks for each product. A direct consequence of this dynamic workflow is that the number of Fireworks in the workflow depends on the type of molecule and the broken bonds. Products resulting from the fragmentation process are filtered for uniqueness using their graph representation and used as inputs to these calculations. Calculations on the products are independent and are thus run in parallel. The workflow supports homolytic and heterolytic bond dissociations of neutral and charged species. In addition, it comes with an option to open bonds that are part of a ring. In this case, the fragmentation process results in a single product fed to the optimization and frequency Fireworks. Users may specify to break one or more bond and open rings in the same workflow, thus outputting more than one BDE value. However, each BDE predicted by the workflow results from one bond broken or opened at a time; otherwise, predicted energies would be less reliable. The workflow gives the option to run a local energy minimization via OpenBabel on the generated fragments before using them as initial guesses for subsequent geometry optimization at the higher level of theory. This is especially useful with ring-opened products to prevent the ring from reclosing during the DFT geometry optimization. After all Fireworks corresponding to the products are completed, computed enthalpies from the frequency Fireworks are passed to a final analysis step. In this step, the BDE of each broken bond is calculated as ${(H}_{frag1}+{H}_{frag2})-{H}_{principal}$, where ${H}_{frag1}$, ${H}_{frag2}$, and ${H}_{principal}$ are the enthalpies of fragment 1, fragment 2, and the principal molecule, respectively. In the case of ring-opening, BDE represents the difference between the enthalpy of the ring-opened product and that of the reactant.

Once BDE calculations are completed, the workflow outputs a summary figure similar to the one displayed in Fig. 5. The figure contains a 2D representation of the reactant with the broken or open bonds highlighted in color and the corresponding calculated BDEs in the form of a bar plot. Benchmarking was performed by computing a set of 30 BDEs using the BDE workflow and comparing the predictions to experimental data found in the iBond database^60^. Sample results from the BDE workflow are shown in Table S2, where a MAE of 0.15 eV is obtained, thereby approaching the underlying uncertainty in experimental measurements^54^.

#### Binding energy

**Figure 6:**
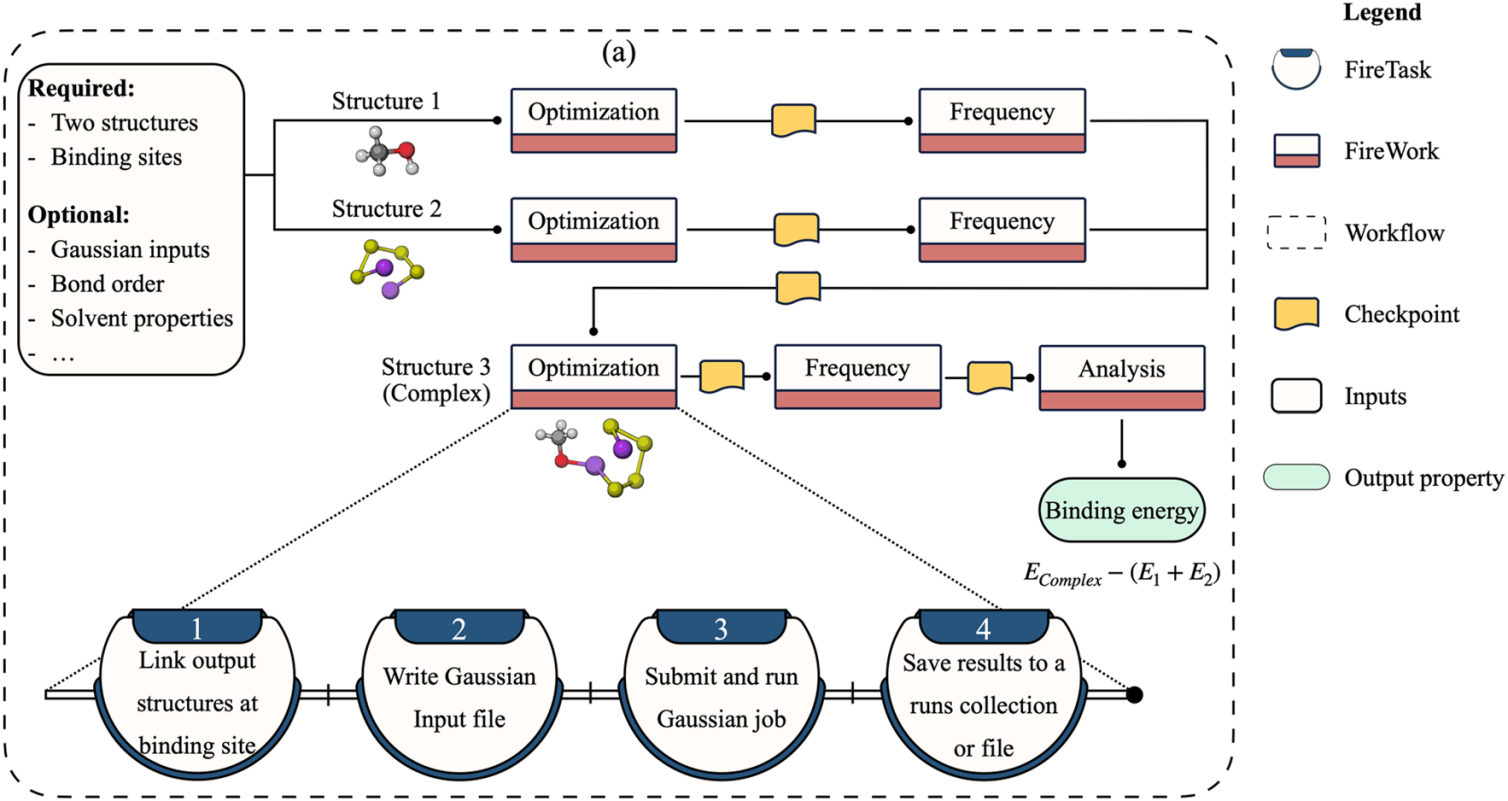
Workflow diagram for the binding energy calculation.

Binding energy (BE) is used in evaluating the anchoring performance of substrates and understanding the adsorption mechanism of molecular species on surfaces. For example, it is critical for screening electrolyte additives for forming solid-electrolyte interphases in lithium-ion batteries^61^ and identifying promising functional groups for electrodes and membranes in lithium-sulfur (Li-S) batteries^62^. We implement a BE workflow (Fig. 6) that provides a quantitative description of the interaction between two structures. Required inputs to this workflow include two structures and the site indexes at which they are expected to bind. The workflow starts with relaxing the two geometries in separate Fireworks executed in parallel. The optimized structures are then used in vibrational frequency Fireworks that provide information about the nature of the stationary point and produce thermodynamic properties. If the stationary points are confirmed to be potential energy surface minima, they are connected to form the complex structure in an automated fashion. By default, a single bond is formed, but users may request to form double or triple bonds as needed for their study. Next, another sequence of optimization and frequency Fireworks are run. The outputs from the frequency Fireworks of the original structures and the formed complex are then post-processed using an analysis Firework to calculate the BE according to ${E}_{complex}-({E}_{1}+{E}_{2})$, where ${E}_{complex}$ is the energy of the molecular complex and ${E}_{1}$ and ${E}_{2}$ are the energies of structures 1 and 2, respectively. If the BE is negative, the formation of the bond is predicted to be exergonic. In addition, the high absolute value of BE corresponds to strong interactions between the two structures. This final analysis Firework gives the option to store the workflow output in a database collection specific to BE calculations. Sample binding energy results obtained from the workflow between lithium polysulfides of different chain lengths and various graphene functionalized materials are shown in Fig. S3. These results provide a rational guideline for screening promising materials for carbon/sulfur cathodes in Li-S batteries to encapsulate lithium polysulfides and reduce the shuttle phenomenon^63^.

#### Redox potentials

**Figure 7:**
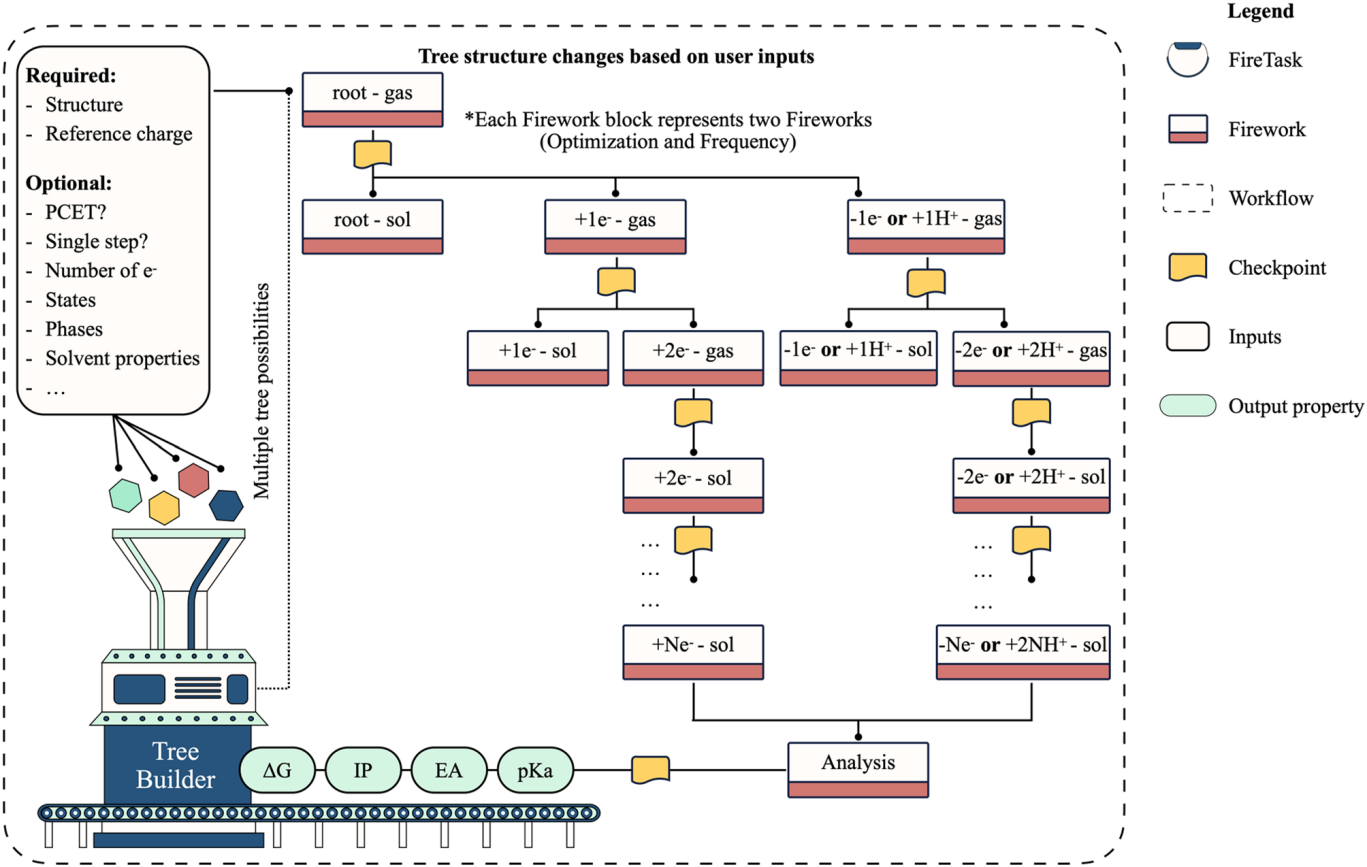
Workflow diagram for redox potential calculations.

Understanding many biological, chemical, and industrial electron-transfer reactions requires a precise knowledge of redox potentials^64^. Redox potentials, including the ionization potential (IP) and electron affinity (EA), refer to the propensity of a molecule to donate or accept an electron and thereby be oxidized or reduced, respectively. There have been significant efforts to rapidly screen optimal redox-active molecules via reliable computational methods and down select molecular candidates for targeted systems^23,38,65^. While these efforts include some of the features we describe below, they are focused on non-aqueous solutions and thus are not well suited for modeling proton-coupled electron-transfer^66^ (PCET) reactions in protic media such as aqueous redox flow batteries. Here, we develop a redox potential workflow that employs an adaptive tree data structure, as shown in Fig. 7. The tree structure, i.e., size (number of nodes), height, and depth, varies based on user inputs, making our design flexible to support different types of redox potential calculations. These methods include HOMO/LUMO, vertical IP/EA, adiabatic IP/EA, and sequential PCET calculations (Table S3). Also supported is the capability to run electron transfer calculations via single-step or multi-step pathways. To request a specific method, the user needs to set the appropriate submission parameters to the workflow. The only required inputs are the structure and its charge at the reference state. Optional inputs include the number of electrons transferred, charge state (cation, anion, or both), phase (gas, solution, or both), whether electron transfer occurs via a single-step or a multi-step process, whether electron transfer is accompanied by hydrogen transfer, i.e., PCET, among many others. Such flexibility to run variations of redox potential calculations, including automated PCET calculations, by simply turning on or off specific input parameters, is, to the best of our knowledge, unique to our design. If a PCET calculation is requested, the user needs to specify the atomic site(s) at which the hydrogen atom(s) is expected to attach. By default, the workflow runs the adiabatic single-step IP/EA, which uses the thermodynamic cycle and optimizes the geometry at each charge state, thus emphasizing high-fidelity calculations.

The user can choose the redox potential calculation method based on the desired accuracy and availability of computational resources. The root node, i.e., the topmost node of the tree, corresponds to calculations on the molecule at the reference state. At this node, optimization and frequency Fireworks are created and run sequentially. Upon completion of these steps, branching is done at the root node to generate the children nodes, at which optimization and frequency Fireworks are again created at different charge states and phases. This process is repeated at each level of the constructed tree until all required calculations have been set up and executed. Children nodes take the optimized geometries from their parents as an initial guess. Fireworks generated at the sibling nodes are run in parallel. It is important to note that the optimization Firework is not always performed at all nodes in every variation of redox potential calculations, such as the vertical IP/EA calculations. For example, for vertical IP/EA calculations, children nodes use the optimized geometry at the parent node in their frequency Fireworks. After calculations at all nodes are completed, a final analysis Firework is carried out to compute redox potentials and other relevant properties. Computed properties include IP, EA, p*K*_a_, and Gibbs free energies of oxidation and reduction in the gas and solution phase. The computed Gibbs free energies of individual molecules/ions include entropic and zero-point vibrational energy corrections. The Gibbs free energy of the free electrons can also be included in these calculations. The properties output by the workflow depend on the redox potential method the user chose upon setting up the workflow. For example, if the user requested a PCET calculation, the workflow outputs EA and p*K*_a_. If the study is limited to the oxidation process, the workflow outputs IP, and so on. The workflow converts the absolute oxidation and reduction potentials to commonly used potential scales, including the standard hydrogen electrode^67^, Li^+^/Li^68^, and Mg^2+^/Mg^69^ to compare them with experimental data. Because the conversion factor depends on several factors like the solvent type^23^, the user may override the default factors and provide additional standard potentials. The workflow stores both the absolute and the converted potentials. As a final step, the workflow stores the output in a collection specific to redox potentials in the database or to a local JSON file. Besides the properties described above, the workflow stores all relevant information like the type of calculation, solvent model, number of electrons, among many others. It is important to note that including the effect of intermolecular interactions has led, in some cases, to substantial variations in redox potentials compared to calculations on isolated molecules surrounded by implicit solvent^22–24^. Therefore, users may choose to append this workflow to the MD workflow to sample charge-transfer complexes and use them for modeling redox reactions.

**Figure 8:**
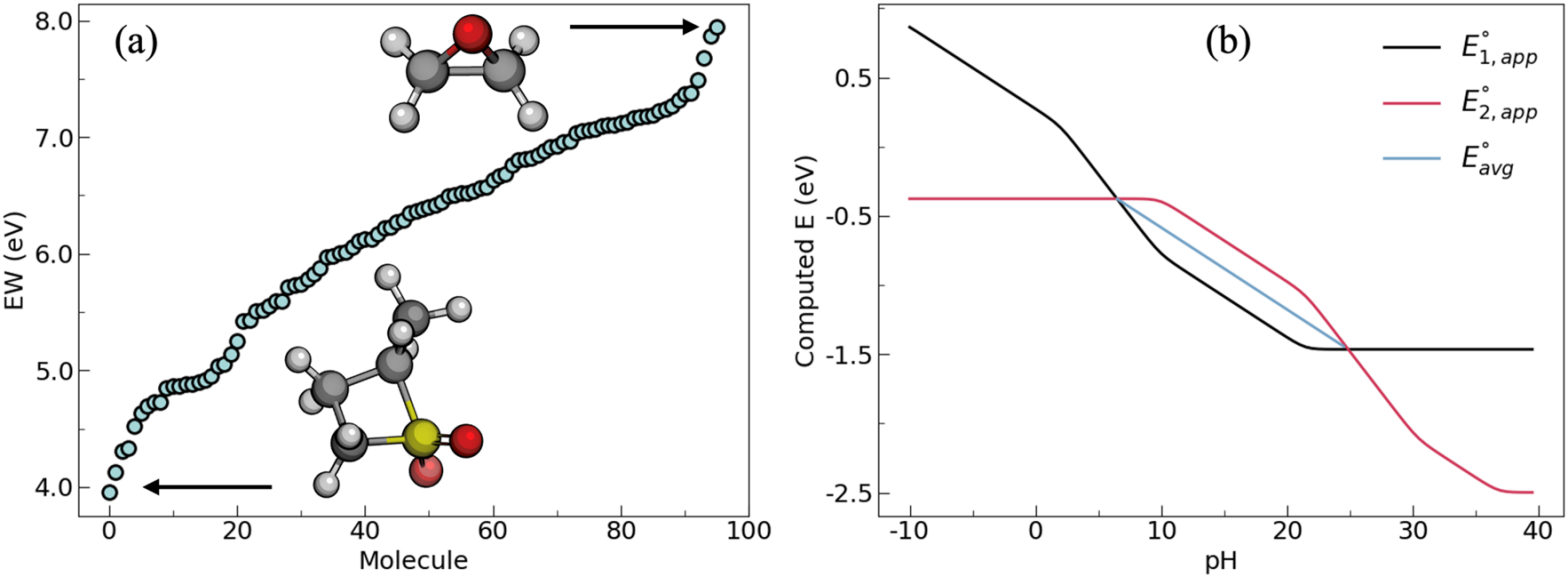
Sample calculations of (**a**) electrochemical stability window of additives and cosolvents relevant to Li-ion battery and (**b**) equilibrium potential diagram resulting from a two-step PCET reaction of a quinone molecule using the redox potential workflow.

We use the redox potential workflow to determine the first adiabatic oxidation and reduction stability of 100 molecules as a test case. Figure 8a shows a sample plot of their computed electrochemical stability window. This library (Table S4) is primarily composed of additives and cosolvents relevant to Li-ion batteries, including but not limited to linear and cyclic carbonates, sulfones, and fluorinated solvents. Initial structures were parsed from the PubChem^70^ database. The calculations took less than 24 h of wall-clock time and generated around 1200 input and output files to determine the electrochemical properties of these compounds. Around 18% of the calculations failed due to errors related to SCF and geometry optimization failures. The automatic error handler fixed 77.8% of the failed calculations, thus improving the success rate from 82 to 96%. However, as noted by Blau et al.^71^, more challenging electrochemically relevant molecules such as radicals and metal-coordinated complexes can increase the risk of calculation failure, thus necessitating the use of a robust error handler like the one implemented here. Figure 8b also shows a sample equilibrium potential diagram constructed from the output of the workflow for the PCET of a quinone molecule.

#### MD workflow

**Figure 9:**
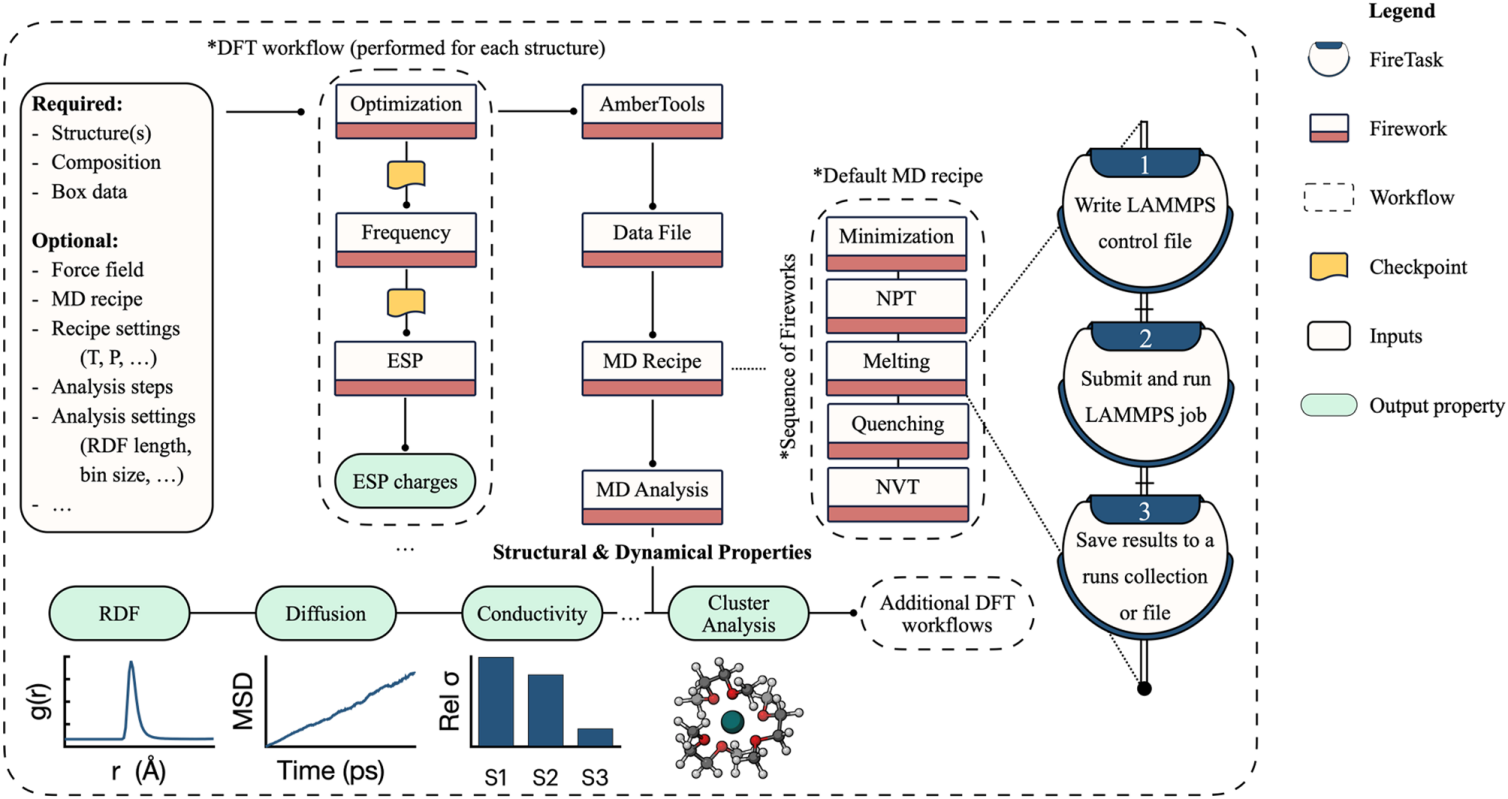
Diagram for the workflow that seamlessly integrates DFT calculations with MD simulations to derive various structural and dynamical properties of liquid solutions.

MISPR includes a standard workflow for performing classical MD simulations of liquid solutions and subsequently deriving various structural and dynamical properties in an automated manner. Support for solid–liquid interfaces is planned for the future. The workflow, displayed in Fig. 9, requires as inputs the number and structure of the individual species comprising a liquid solution of interest, the geometry information of the simulation box, and details of the force field parameters. The workflow either takes the force field parameters as direct inputs in the form of a user-defined dictionary, retrieves them from an in-house database of force field parameters based on input query criteria, or derives them by utilizing the output of the ESP workflow. The user may choose between the three force field options independently for each species in the system. By default, the workflow runs the ESP workflow described earlier to optimize the geometry of the species and compute their ESP charges. It then fits the output charges to the RESP model and generates GAFF parameters by utilizing programs from AmberTools^28^. The workflow proceeds by extracting the force field parameters from the generated files and storing them in a Python dictionary via the ParmEd package^72^. An example of the format of the force field dictionary is shown in Fig. S4. In the next step, the workflow passes the optimized structures along with the details of the system (i.e., number of molecules of each type and geometry of the system box) to the *PackmolRunner* class in pymatgen to run Packmol^73^ and build the initial system configuration. The workflow then writes the data file that contains the basic information needed to run a LAMMPS simulation, including the initial atomic coordinates, molecular topology, and force field parameters. While the workflow uses GAFF by default, it supports other types of force fields (e.g., OPLS-AA^74^ and CHARMM^75^). In these cases, the user needs to provide their parameters either as direct inputs or, if available, retrieve them from the in-house database.

The workflow uses a default set of ensembles as the main recipe for equilibrating the liquid solutions to derive various structural and dynamical properties. This recipe includes sequential Fireworks for a two-step energy minimization procedure using steepest descent followed by conjugate gradient, NPT equilibration run, melting and quenching to ensure that the molecules are not trapped in a metastable state, and an NVT production run. The ensembles used in this recipe are defined by parameters written in a LAMMPS control file. The user may alter the default settings (e.g., temperature, pressure, timestep, etc.) for each Firework as needed. These Fireworks are made up of a sequence of Firetasks for writing the LAMMPS control file, submitting and running the job on supercomputing resources, and post-processing the output. The workflow is flexible enough to allow the user to define and run any series of LAMMPS calculations other than the default MD recipe.

Several analysis Fireworks are then performed to post-process the output and trajectory files containing atomic positions, velocities, and forces and compute various ensemble properties. To this end, the workflow utilizes MDPropTools, a standalone in-house Python package for the temporal analysis of LAMMPS outputs. By relying on our own MD analysis package, we maintain full compatibility with the MISPR package and enable the extendibility of the modular MD analysis codes. Tight integration between MDPropTools and MISPR allows getting invaluable insights from MD simulations while freeing the user from having to be acquainted with the details of the I/O operations associated with the output and trajectory files. In addition, appending analysis Fireworks to the MD workflow is especially important when high-throughput screening calculations are performed since it is impractical to rely on manual intervention when thousands of trajectories are analyzed. MDPropTools utilizes Python libraries such as NumPy^76^ and SciPy^77^, which provide optimized classes and functions for handling multidimensional arrays and linear algebra routines. MDPropTools also uses the Numba library^78^ to create faster compiled code with minimal changes to the standard Python syntax.

Examples of supported dynamical properties in MDPropTools include self-diffusion coefficients, viscosity, and ionic conductivity. MDPropTools also provides codes for calculating other properties such as radial distribution function (RDF), coordination number, number density, residence time, and dielectric constant. It contains utility functions to convert data from the output and trajectory files to SI units. All unit styles supported by LAMMPS are convertible by these utility functions. By default, the MD workflow in MISPR computes the RDF between all possible pairs of atoms in the solution and the self-diffusion coefficients of the molecular species. However, the user may choose which properties to compute by providing their corresponding input parameters. Therefore, the number and type of Fireworks in the MD workflow varies based on the inputs. In addition, the user may skip the analysis step and perform it independently of MISPR. An important feature implemented in MDPropTools is the ability to extract atomic clusters corresponding to the solvation shell of species of interest using a cutoff radius either provided as input or automatically extracted from the RDF. These clusters represent a realistic chemical environment of the species in solution. Therefore, they can be fed to the DFT workflows for a more accurate prediction of properties compared to similar calculations on isolated species. Besides utilizing the ESP workflow to assign partial atomic charges for building GAFF parameters, the cluster analysis step at the end of the MD simulations serves as a bridge for integrating MD simulations with DFT workflows in MISPR.

We use the MD workflow to predict the coordination number and diffusion coefficients in systems that have been extensively studied both computationally and experimentally for lithium-ion batteries. The systems consist of 1 M lithium bis(trifluoromethylsulfonyl)imide (LiTFSI) salt dissolved in various organic and inorganic solvents (Fig. S5). The code in Fig. S6 demonstrates how the task of performing and managing these calculations is reduced to a single submission procedure starting from the individual structures defined in XYZ file format. Sample plots showing the computed properties are shown in Fig. S5.

### Database

MISPR stores all inputs and outputs of the DFT and MD workflows in MongoDB^79^, a document-oriented NoSQL datastore. In MongoDB, documents are grouped into collections according to their structure, but they also have a flexible schema. MongoDB allows user-friendly queries and easy updates to schemas compared to rigid, tabular data models used by relational databases.

**Figure 10:**
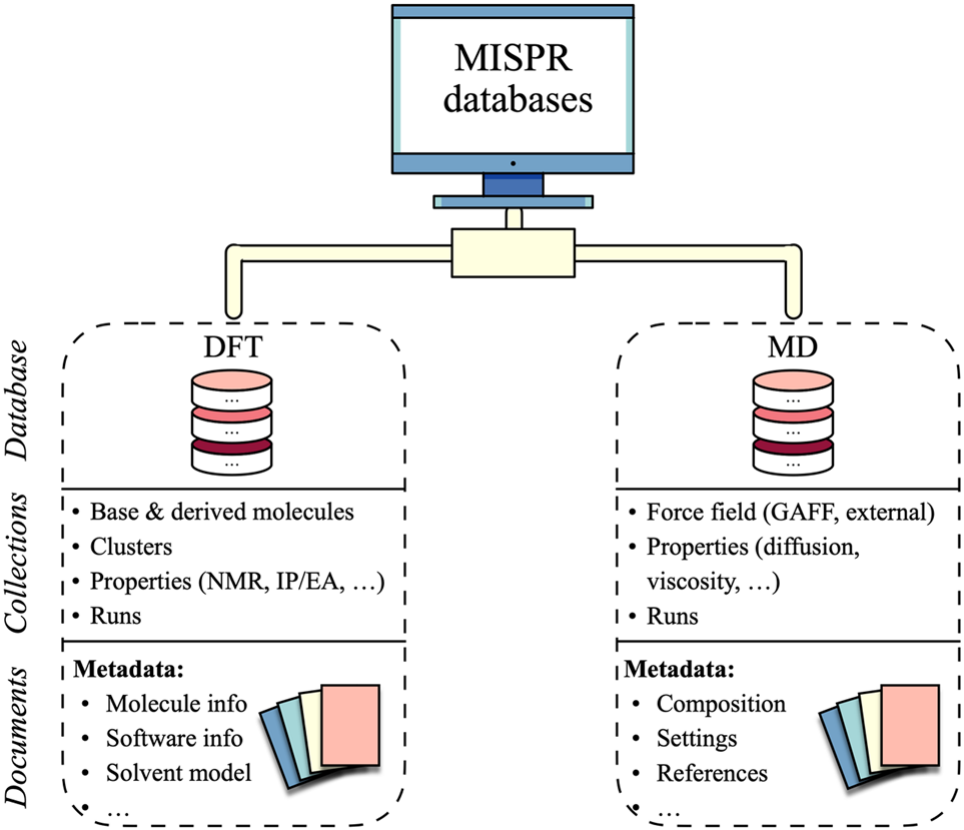
An overview of MISPR databases, including the collections in each database and the type of metadata stored in the documents of these collections.

Figure 10 shows an overview of MISPR databases. At the time of writing, MISPR includes two databases: *DFT* and *MD*. MISPR handles heterogeneity of data collected from the workflows by converting it into a standardized, code-independent format for the relevant properties. However, storing raw or standardized data without any metadata would be futile because such data can neither be easily queried nor used for data analytics and ML models. For example, a predicted BDE is of limited use if there is not enough information about the level of theory, the solvation model, the structure geometry, etc. Therefore, MISPR stores the metadata necessary for storing and retrieving the predicted properties. MISPR also stores the provenance during execution, thus implicitly documenting the logical flow of calculations and ensuring reproducibility of results. Other important stored information include versions of the codes that generated the data (i.e., Gaussian, LAMMPS, and MISPR) and submission metadata (e.g., author). Users may also store custom attributes for later querying and filtering (e.g., a tag specifying the project title, comments, etc.). MISPR creates appropriate indexes to enable the efficient execution of queries. Figure S7 shows examples of documents from the *DFT* and *MD* databases. In addition to database storage, MISPR writes local JSON files with identical content as the database documents. Creating these files allows users to quickly check outputs, collect data without accessing the database, and exchange data with other parties.

In the *DFT* database, a collection of MongoDB documents called *runs* contains the output data for all calculations. Each document includes metadata (e.g., job type, wall time, datetime, automatically generated structure metadata like the InChI and SMILES representations), input data (e.g., initial geometry, functional, basis set), and parsed output data (e.g., final geometry, energy, dipole moment). Separate collections are dedicated to computed properties (e.g., NMR tensors, binding energies, redox potentials), often resulting from multiple calculations on the same molecule or across different molecules. To keep track of the property "history", MISPR saves the *ObjectID* of the *runs* documents corresponding to the calculations performed to derive the final property. Other collections in the *DFT* database include a collection for molecules (isolated molecules, derived molecules, and clusters) and a collection for functional groups. Documents in these collections include the geometry of the molecules and their metadata. Users may provide query criteria (*e.g.*, *ObjectID*, InChI code) to the workflows to directly retrieve molecules from these collections and use them as inputs to the DFT and MD workflows.

Similar to the *DFT* database, the *MD* database contains a *runs* collection for storing information about LAMMPS simulations such as the type and number of molecules, the job type (e.g., NPT, NVT, etc.), system geometry (*e.*g., box lengths), and control settings (e.g., timestep, temperature, pressure, etc.). Another collection in the MD database is for properties derived from MD simulations. Documents in this collection include input parameters for the analysis codes, computed properties (e.g., diffusion coefficients), and information about the chain of steps leading to the final property. Because it is desirable to run multiple replicates of the same system to obtain reliable statistics, documents in this collection also contain the run replicate number and the seeds used to initialize the positions and velocities of each particle. Force field parameters are stored in a separate collection in the MD database. This collection includes GAFF parameters that are built using AmberTools as well as other types of force fields that are directly input by the user. Documents in this collection follow the same format as the force field parameter dictionary used in the data file preparation step, as shown in Fig. S4. Because force field parameters are heterogeneous in origin, the force field documents include a field for their source in the form of a digital object identifier. Other metadata stored include structural information such as the SMILES representation and molecular formula.

## Methods

All DFT calculations were performed using Gaussian 16 Revision C.01. Initial structures for the NMR calculations were sourced from the PubChem database. We compiled the experimental data from the Spectral Database for Organic compounds (SDBS)^80^ and a previous study^81^, with a restriction that the experimental ^13^C and ^1^H NMR data are measured in chloroform solvent and referenced to tetramethylsilane (TMS). We also used compounds spanning a broad array of chemical classes and distribution of molecular sizes. Detailed information about the library is given in Table S5. Isotropic magnetic shielding tensors were computed using the NMR workflow in a chloroform solvent at the ωB97X/def2-TZVP level of theory. An ultrafine integration grid and tight convergence criteria were used for the optimization step of the workflow. Solvent effects were treated using the polarizable continuum model (PCM)^82–84^. Optimized geometries were confirmed to be minimum structures by vibrational frequency calculations at the same level of theory. ^13^C and ^1^H chemical shifts were then computed relative to TMS using the GIAO^45–49^ formalism. Calculated chemical shifts were then compared to experimental values from SDBS^80^ and a previous study^81^.

The PBE1PBE functional and 6–31 + G* basis set were adopted for the BE calculations between lithium polysulfides (Li_2_S_x_, x = 2, 4, 8) and various functionalized graphene models. The D3 version of Grimme's dispersion^85^ was employed to account for long-distance van der Waals interactions. The structure of the pristine graphene model and the Li_2_S_x_ species are displayed in Fig. S8. The functionalized graphene model was derived by attaching each of the tested functional groups to an edge carbon atom in the pristine graphene model.

BDE calculations were performed in the gas phase at the ωB97X/def2-TZVP level of theory for a set of 30 BDEs from the iBond database^60^. The structure of the molecules along with the broken bonds are shown in Table S2.

The SMD^86^ solvation model was used in the IP and EA calculations to estimate the free energies of formation of the oxidized, reduced, and initial structures. Three solvents covering a wide range of dielectric constants were tested: (1) tetrahydrofuran (ε = 7.4257), (2) acetone (ε = 20.493), and (3) water (ε = 78.355). We found a more significant dependence of the IP and EA on the dielectric constant for ε between 7.4257 and 20.493. On the other hand, the solvation contribution is smaller (mean absolute difference < 0.1 eV) for ε between 20.493 and 78.355, which is in agreement with previously reported results^87^. Acetone has a dielectric constant that is close to many commonly used mixed electrolytes in Li-ion batteries^87^. We also tested the effect of two basis sets (6–311++G** versus def2-TZVPD) on the computed redox potentials. We found a better agreement with experimental measurements with the def2-TZVPD basis set accompanied by a slightly higher computational cost. Based on a benchmarking study on 100 combinations of levels of theory, Blau et al. reported that B3LYP/def2-TZVPD combined with the SMD model is adequate for the calculation of redox potentials of electrochemically relevant molecules^71^. Therefore, we proceeded with acetone as the solvent and B3LYP/def2-TZVPD level of theory for the rest of the redox potential calculations. Benchmarking results on the effect of the solvent type and basis set are shown in Table S6. Optimization and frequency calculations were performed in vacuum and in solution at each charge state for the first electron transfer reactions for the isolated molecules. Equations used for computing the free energies and redox potentials from the thermodynamic cycle are presented in Table S3. The predicted redox potentials were then converted to the commonly used Li^+^/Li potential scale by subtracting 1.4 V^87^. Finally, the PCET calculations for quinone were performed in water at the B3LYP functional, 6–311++G** basis set, and the CPCM^88,89^ solvation model. Optimization and frequency calculations were performed in the gas and solution phases for the first and second hydrogen and electron transfer reactions. The equilibrium potential diagram shown in Fig. 8b was constructed from the computed reduction potentials and the p*K*_a_ values according to the equations shown in Table S3.

MD simulations were performed using the LAMMPS MD simulation package version 3Mar2020. The default MD recipe in MISPR was used for the simulations. We considered 1 M LiTFSI in various solvents at 1 atm and 25 ℃ in a cubic box of size 5*5*5 nm^3^ with periodicity in XYZ direction. GAFF parameters were used for the anion and the solvent molecules, while parameters by Aqvist^90^ were used for the cation. Water molecules were described by the rigid SPC/E^91^ water potential, and the constraints for the rigid water model were fulfilled using the SHAKE^92^ algorithm. Lennard–Jones interactions were truncated at a cutoff distance of 1.0 nm. The particle–particle particle-mesh (PPPM)^93^ method was used to handle long-range electrostatic interactions using a cutoff of 1.0 nm. NPT simulations were performed for 1 ns with the Nosé/Hoover^94,95^ thermostat and barostat. The systems were melted to 227 ℃ for 500 ps and quenched for 2 ns to achieve a temperature of 25 ℃ and ensure that the molecules are not trapped in the metastable state. NVT simulations were performed for 5 ns and molecular trajectories were sampled every 50 ps, resulting in around 100 configurations per system, for a total of 1000 configurations, from which properties of interest were calculated.

## Supplementary Information

Supplementary Information.

## Data Availability

The molecular libraries used in the NMR, BDE, and redox potential calculations in this paper are included in the Supplementary Information. The geometry of the optimized structures used in these calculations in XYZ format are available in the GitHub repository at https://github.com/rashatwi/mispr-dataset. In addition, calculated ESP charges, ^13^C and ^1^H NMR chemical shifts, BDEs, IP, EA, and electrochemical windows in gas and/or solution phases, are also made publicly available in the same repository in CSV format.

## Code availability

DFT calculations were performed using the proprietary Gaussian code, while MD simulations were performed using the open-source LAMMPS-codes. The MISPR and MDPropTools packages developed and described in this work are publicly available free of charge at https://github.com/molmd/mispr and https://github.com/molmd/mdproptools, respectively.
