# Supplementary material for: MISPR: an open-source package for high-throughput multiscale molecular simulations
Source: Sci Rep. 2022 Sep 21;12:15760. doi: 10.1038/s41598-022-20009-w (PMC9492707; doi:10.1038/s41598-022-20009-w)
Supplement: Supplementary file 1 — Supplementary Information. [file 41598_2022_20009_MOESM1_ESM.pdf]

MISPR: An open-source package for high-throughput multiscale molecular simulations

Rasha Atwi[1], Matthew Bliss[1], Maxim Makeev[1], Nav Nidhi Rajput[1]

[1]Department of Materials Science and Chemical Engineering, Stony Brook University, Stony Brook, New York 11794, United States

* To whom correspondence should be addressed:

Nav Nidhi Rajput; Email: navnidhi.rajput@stonybrook.edu

Table of Contents

1. File storage 3

Figures

Fig. S1 Code snippet of the submission of a bond dissociation energy calculation ____ 4

Fig. S2 Distribution of error associated with (a) ^{13}C and (b) ^{1}H chemical shift predictions in a chloroform solvent _ 6

Fig. S3 Binding energy between lithium polysulfides (Li_2S_x, x = 2, 4, 8) and a functionalized graphene model at the PBE1PBE/6-31+G* level of theory ____ 10

Fig. S4 Example of a (a) force field dictionary and (b) the labeled structure of the corresponding molecule ____ 20

Fig. S5 (a) Solvents used for MD simulations with 1 M lithium bis(trifluoromethylsulfonyl)imide, (b) coordination number, and (c) diffusion coefficients computed using MISPR ____ 20

Fig. S6 Code snippet of the submission of a hybrid DFT-MD workflow for a lithium bis(trifluoromethylsulfonyl)imide in monoglyme liquid solution using the default MD recipe in MISPR, GAFF parameters for the anion and solvent, and user-defined parameters for the cation ____ 21

Fig. S7 Example of (a) binding energy document from the DFT database and (b) MD document ____ 22

Fig. S8 Structure of Li_2S_x (x = 2, 4, 8) species and pristine graphene model used in the BE calculations ____ 34

Tables

Table S1 Sample ESP and RESP charges for a monoglyme molecule computed at the B3LYP/6-31+G* level of theory and compared to OPLS charges ____ 5

Table S2 BDE results with experimental data retrieved from the iBond database[1] and computational data obtained using MISPR; broken bonds are highlighted in the 2D structure of the molecule ____ 7

Table S3 Methods supported by the redox potential workflow in MISPR; redox potential calculations can be done in any phase and at any charge state (i.e., any number of electron and hydrogen transfers) ____ 11

Table S4 Details of the library used for the redox potential calculations ____ 12

Table S5 Details of the library used for the NMR calculations along with the experimental measurements made in chloroform solvent at 298 K and referenced to tetramethylsilane ____ 23

Table S6 Benchmarking results of the adiabatic IP/EA calculations using the SMD solvation model; potentials are reported relative to the Li^+/Li potential scale ____ 34

1. File storage

Every time a workflow is created and executed, the infrastructure automatically creates a single folder for the workflow with an assigned unique name that can either be provided by the user or automatically determined from the structural representation of the molecule or system of molecules (*e.g.*, molecular formula). Within this main folder, multiple subfolders are created for calculation steps included in the workflow (*e.g.*, geometry optimization and frequency calculation in DFT, ensemble type in MD) with a name corresponding to the type of calculation. A subfolder is also created for the final analysis performed to derive a property (such as binding energy, diffusion, or viscosity) using data parsed from the individual steps. Any file, if present, associated with a calculation is stored within its corresponding subfolder. The number and names of subfolders created are workflow-specific. All folders resulting from a workflow are confined within a parent directory configured when loading and setting the workflow input parameters.

```python
from fireworks import LaunchPad

from mispr.gaussian.workflows.base.bde import get_bde

lpad = LaunchPad.auto_load()

wf = get_bde(mol_operation_type="get_from_file",
             mol="dme.xyz",
             bonds=[(1, 6)],
             open_rings=False,
             process_mol_func=False,
             skips=None,
             mol_name="dme",
             solvent_gaussian_inputs="(SMD, Solvent=TetraHydroFuran)",
             save_to_db=False,
             save_to_file=True,
             visualize=True,
             tag="bde-example")
lpad.add_wf(wf)
```

Fig. S1 Code snippet of the submission of a bond dissociation energy calculation

Table S1 Sample ESP and RESP charges for a monoglyme molecule computed at the B3LYP/6-31+G* level of theory and compared to OPLS charges

Number	Atom	Computed ESP charges	Derived RESP charges	OPLS charges
1	O	-0.450597	-0.396634	-0.4
2	O	-0.448858	-0.396634	-0.4
3	C	0.168498	0.106870	0.14
4	C	0.173399	0.106870	0.14
5	C	0.10666	0.057713	0.11
6	C	0.096242	0.057713	0.11
7	H	0.0358	0.047255	0.03
8	H	0.036371	0.047255	0.03
9	H	0.033803	0.047255	0.03
10	H	0.034374	0.047255	0.03
11	H	0.05954	0.045847	0.03
12	H	0.021866	0.045847	0.03
13	H	0.021589	0.045847	0.03
14	H	0.025167	0.045847	0.03
15	H	0.060705	0.045847	0.03
16	H	0.025442	0.045847	0.03

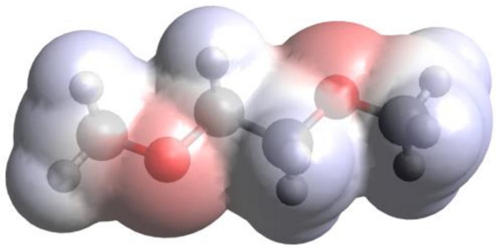

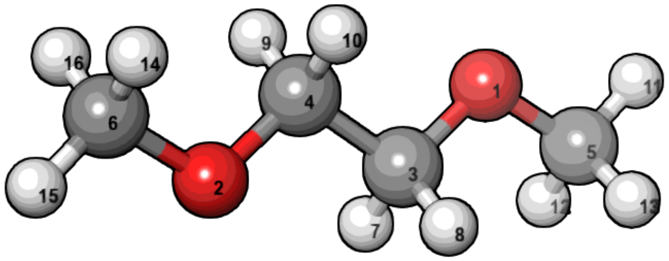

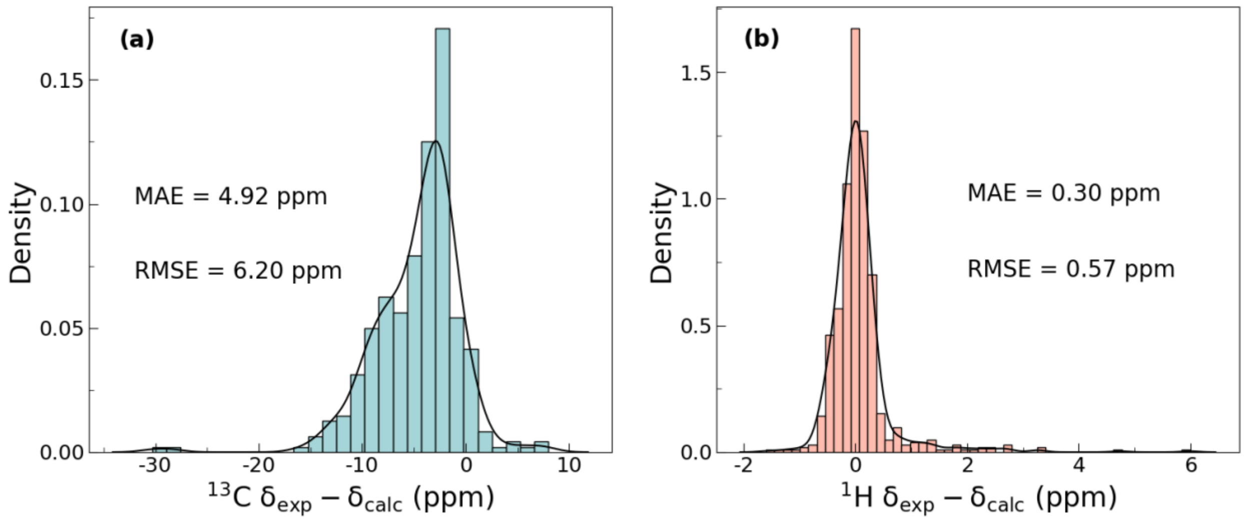

Fig. S2 Distribution of error associated with (a) ^{13}C and (b) ^{1}H chemical shift predictions in a chloroform solvent

Table S2 BDE results with experimental data retrieved from the iBond database[1] and computational data obtained using MISPR; broken bonds are highlighted in the 2D structure of the molecule

Bond number	InChI code	Molecule name	2D representation	Experimental BDE (eV)	Computed BDE (eV)	Absolute error (eV)
1	InChI=1S/CH4/h1H4	methane	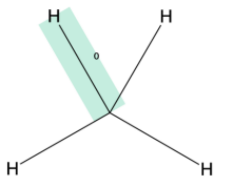	4.5150	4.4980	0.0169
2	InChI=1S/CHN/c1-2/h1H	formonitrile	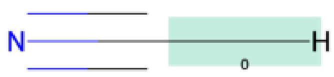	5.3750	5.6117	0.2367
3	InChI=1S/C2H6/c1-2/h1-2H3	ethane	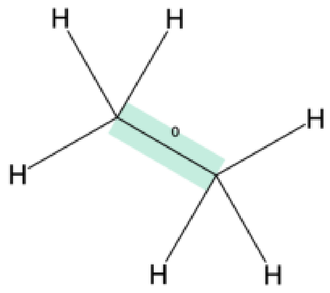	3.8786	3.9347	0.0561
4	InChI=1S/CH3F/c1-2/h1H3	fluoromethane	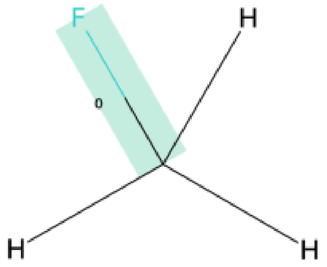	4.7214	4.7687	0.0473
5	InChI=1S/CH3F/c1-2/h1H3	fluoromethane	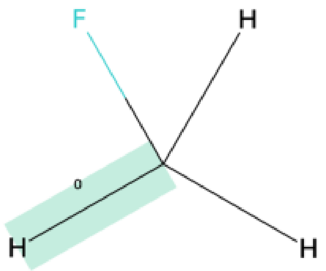	4.7988	4.2804	0.5183
6	InChI=1S/C2H3N/c1-3-2/h1H3	isocyanomethane	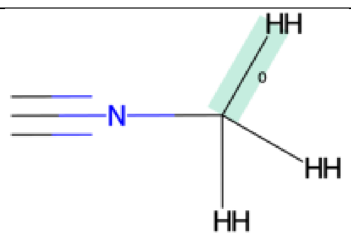	4.1581	4.0451	0.1129
7	InChI=1S/CH2N2/c2-1-3/h2H2	cyanamide	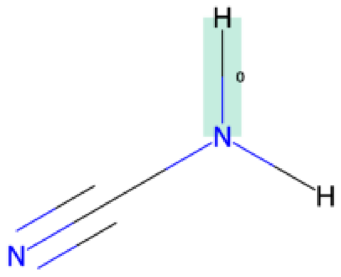	4.2570	4.0311	0.2258
8	InChI=1S/C2H6O/c1-3-2/h1-2H3	methoxymethane	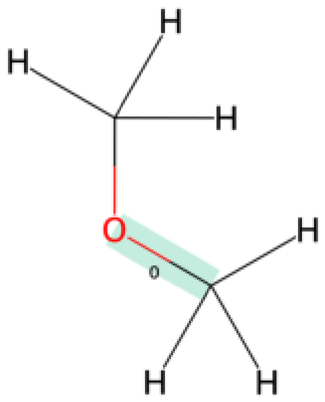	3.6163	3.5138	0.1024
9	InChI=1S/C3H7F/c1-2-3-4/h2-3H2,1H3	1-fluoropropane	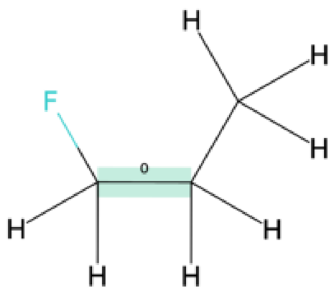	4.3645	3.8951	0.4694
10	InChI=1S/C3H7F/c1-2-3-4/h2-3H2,1H3	1-fluoropropane	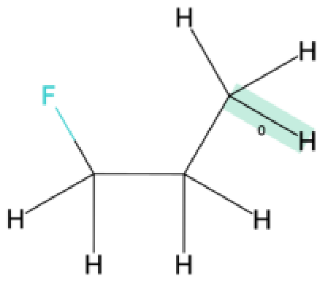	3.9388	4.3316	0.3928

11	InChI=1S/CH4S/c1-2/h2H,1H3	methanethiol	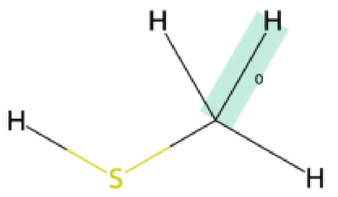	3.9732	4.0309	0.0577
12	InChI=1S/CH4S/c1-2/h2H,1H3	methanethiol	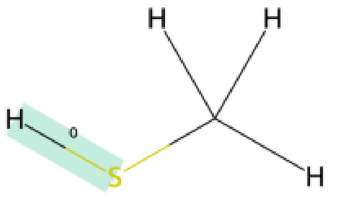	3.7582	3.6603	0.0978
13	InChI=1S/CH4O2/c1-3-2/h2H,1H3	hydroperoxymethane	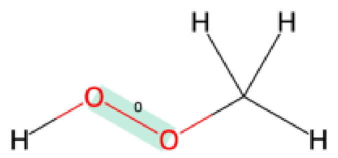	1.9436	1.7591	0.1844
14	InChI=1S/C3H7F/c1-3(2)4/h3H,1-2H3	2-fluoropropane	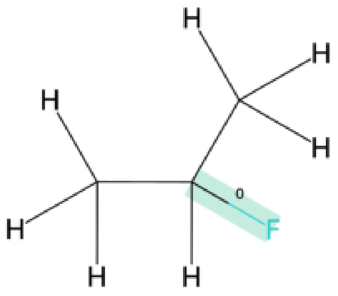	4.7343	4.9154	0.1811
15	InChI=1S/C2H5F/c1-2-3/h2H2,1H3	fluoroethane	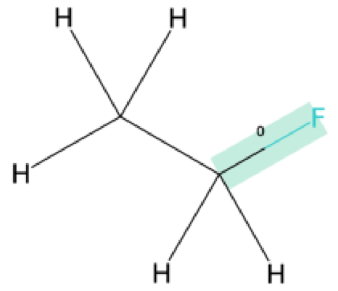	4.6311	4.8545	0.2234
16	InChI=1S/CH3Cl/c1-2/h1H3	chloromethane	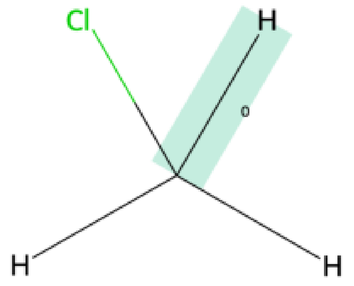	4.3000	4.2064	0.0935
17	InChI=1S/C3H2N2/c4-2-1-3-5/h1H2	propanedinitrile	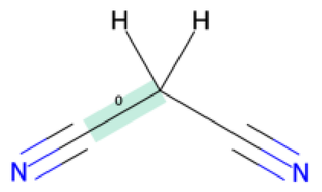	4.3817	4.7628	0.3811
18	InChI=1S/C5H12O/c1-2-3-4-5-6/h6H,2-5H2,1H3	pentan-1-ol	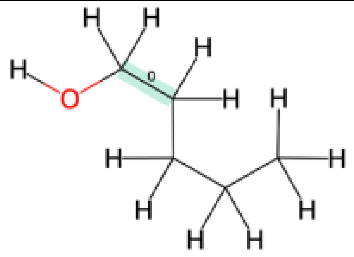	3.6550	3.6437	0.0112
19	InChI=1S/CH3Cl/c1-2/h1H3	chloromethane	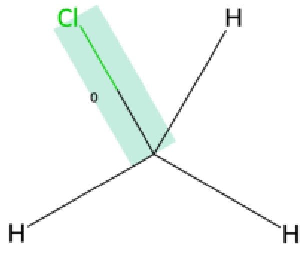	3.5991	3.6438	0.0447
20	InChI=1S/C12H9N/c13-9-8-11-6-3-5-10-4-1-2-7-12(10)11/h1-7H,8H2	2-naphthalen-1- ylacetonitrile	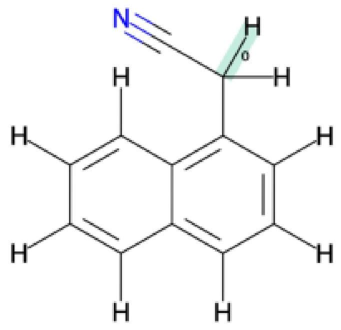	3.4959	3.4197	0.0761
21	InChI=1S/CH2O3/c2-1-4-3/h1,3H	peroxyformic acid	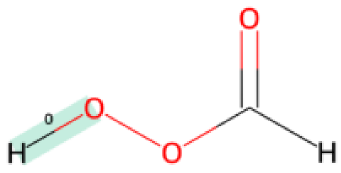	4.2355	3.7367	0.4987
22	InChI=1S/C4H10O/c1-2-3-4-5/h5H,2-4H2,1H3	butan-1-ol	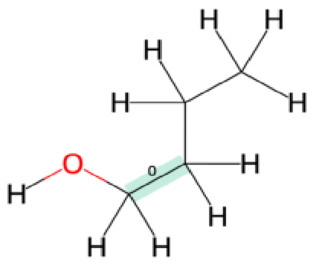	3.6722	3.5984	0.0737

23	InChI=1S/C2H2ClF3/c3-1-2(4,5)6/h1H2	2-chloro-1,1,1-trifluoroethane	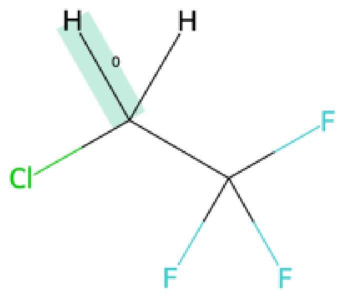	4.3774	4.1860	0.1913
24	InChI=1S/C5H12/c1-4-5(2)3/h5H,4H2,1-3H3	2-methylbutane	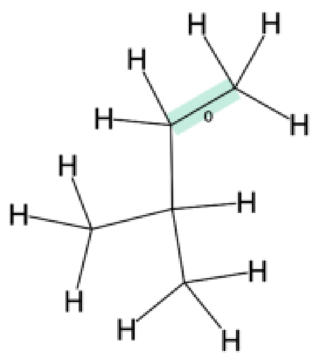	3.7840	3.8374	0.0534
25	InChI=1S/C3H4O/c1-2-3-4/h2H,1H3	Methylketene	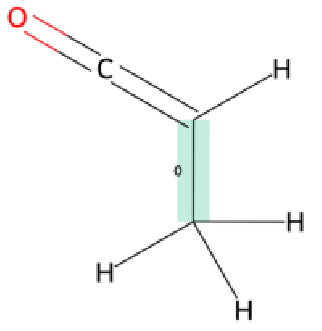	4.0119	4.0019	0.0099
26	InChI=1S/C3H8/c1-3-2/h3H2,1-2H3		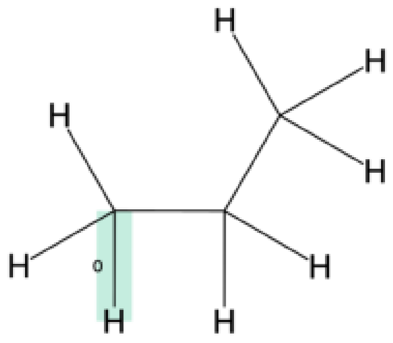	4.2183	4.3126	0.0943
27	InChI=1S/C4H10/c1-4(2)3/h4H,1-3H3		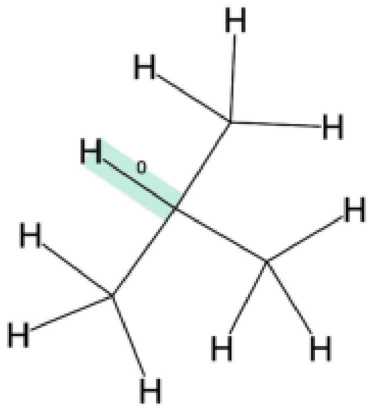	4.1151	4.0563	0.0587
28	InChI=1S/C6H14/c1-3-5-6-4-2/h3-6H2,1-2H3		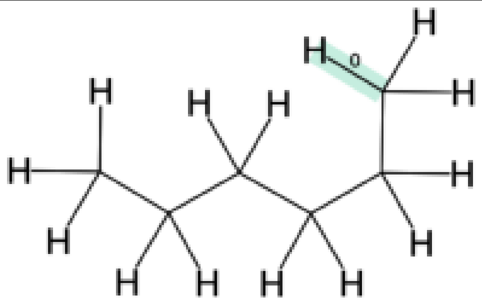	4.2570	4.3373	0.0803
29	InChI=1S/C5H12/c1-4-5(2)3/h5H,4H2,1-3H3		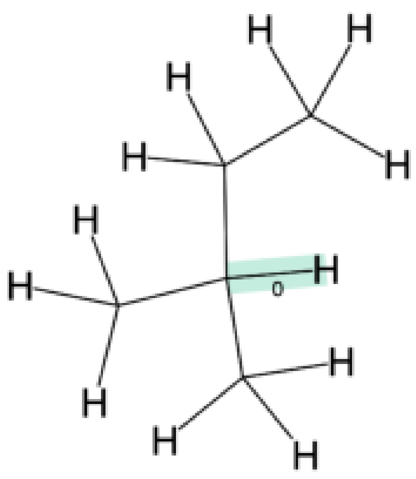	4.1194	4.0778	0.0415
30	InChI=1S/C7H16/c1-4-6-7(3)5-2/h7H,4-6H2,1-3H3/t7-/m0/s1		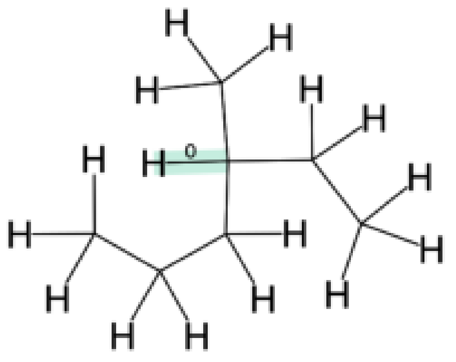	4.0807	4.0971	0.0164

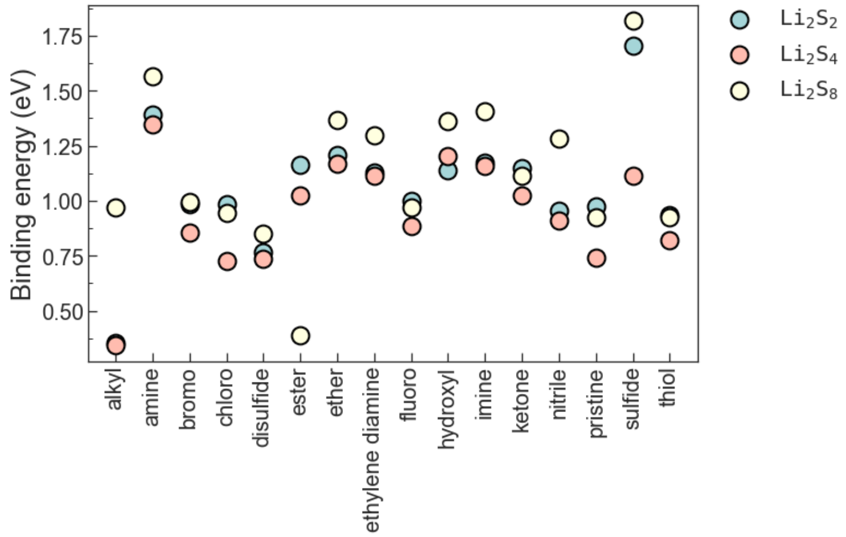

Fig. S3 Binding energy between lithium polysulfides (Li_2S_x, x = 2, 4, 8) and a functionalized graphene model at the PBE1PBE/6-31+G* level of theory

Table S3 Methods supported by the redox potential workflow in MISPR; redox potential calculations can be done in any phase and at any charge state (*i.e.*, any number of electron and hydrogen transfers)

Method	Notes	Example scheme(s)	Equations	Abbreviations
HOMO/LUMO	Geometry optimization is done for the molecule at the reference state to calculate the HOMO/LUMO energies		IP = HOMO<br>EA = LUMO	IP: ionization potential (eV)<br>EA: electron affinity (eV)
Direct electron transfer	Geometry optimization and frequency calculation are done at each charge state in one phase specified by the user (vacuum or solution)	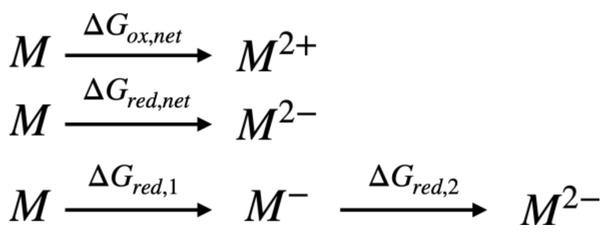	$\Delta G_{ox,net} = G(M^{2+}) - G(M)$ $IP = \frac{\Delta G_{ox,net}}{2F}$<br>$\Delta G_{red,net} = G(M^{2-}) - G(M)$ $EA = -\frac{\Delta G_{red,net}}{2F}$<br>$\Delta G_{red,1} = G(M^{-}) - G(M)$ $EA_1 = -\frac{\Delta G_{red,1}}{F}$<br>$\Delta G_{red,2} = G(M^{2-}) - G(M^{-})$ $EA_2 = -\frac{\Delta G_{red,2}}{F}$	$\Delta G_{ox,net}$: Net Gibbs free energy oxidation<br>$\Delta G_{red,net}$: Net Gibbs free energy of reduction<br>$\Delta G_{red,1}$: Gibbs free energy of reduction for the first electron transfer<br>$\Delta G_{red,1}$: Gibbs free energy of reduction for the second electron transfer<br>EA_1: electron affinity from the first electron transfer<br>EA_2: electron affinity from the second electron transfer
Vertical IP/EA	Geometry optimization is done for molecules at the reference state only at each phase specified by the user<br>Frequency calculation is done at each charge state and phase	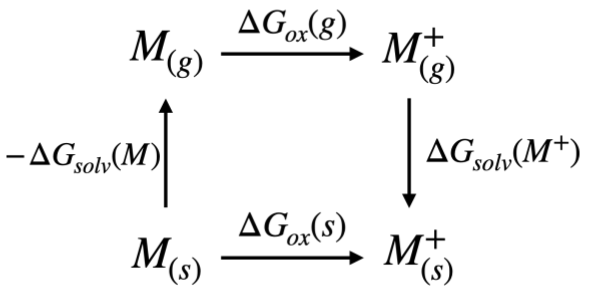	$G = H - T\Delta S = E_{SCF} + E_{ZPVE} + H_{corr} - T\Delta S_{corr}$<br>$\Delta G_{ox}(g) = G(M^{+}_{(g)}) - G(M_{(g)})$<br>$\Delta G_{red}(g) = G(M^{-}_{(g)}) - G(M_{(g)})$<br>$\Delta G_{ox}(s) = \Delta G_{ox}(g) + \Delta G_{solv}(M^{+}) - \Delta G_{solv}(M)$<br>$\Delta G_{red}(s) = \Delta G_{red}(g) + \Delta G_{solv}(M^{-}) - \Delta G_{solv}(M)$<br>$IP = \frac{\Delta G_{ox}(s)}{nF}$ $EA = -\frac{\Delta G_{red}(s)}{nF}$	E_{SCF}: energy<br>E_{ZPVE}: zero-point vibrational energy correction<br>S_{corr}: correction to entropy<br>H_{corr}: correction to enthalpy<br>$\Delta G_{ox}(g)$: Gibbs free energy of oxidation in the gas phase<br>$\Delta G_{red}(g)$: Gibbs free energy of reduction in the gas phase<br>ΔG_{solv}: Free energy of solvation<br>$\Delta G_{ox}(s)$: Gibbs free energy of oxidation in the solution phase<br>$\Delta G_{red}(s)$: Gibbs free energy of reduction in the solution phase<br>F: Faraday constant<br>n: Number of electrons
Adiabatic IP/EA	Geometry optimization and frequency calculation are done at each charge state and phase	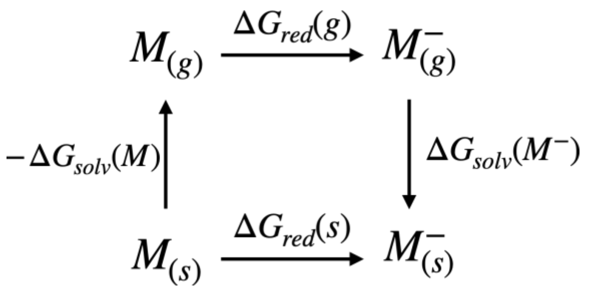		
Sequential proton-coupled electron transfer (PCET)	Direct as well as adiabatic calculations are supported; Number of hydrogen transfer steps is assumed to be equal to the number of electron transfer steps	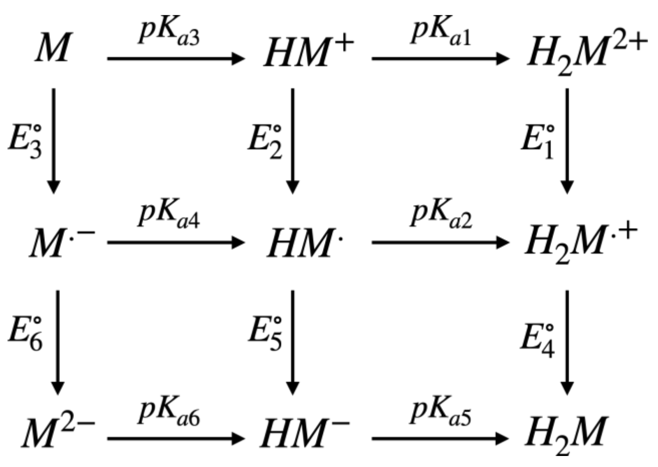	$pK_a = \frac{\Delta G}{2.303RT}$ $K_a = 10^{-pK_a}$<br>$E^\circ = -\frac{\Delta G}{nF} + E^\circ_{ref}$<br>$E^\circ_{1,app} = E^\circ_2 + \frac{RT}{F} ln\left(\frac{\frac{K_{a4}}{[H^+]} + 1 + \frac{[H^+]}{K_{a2}}}{\frac{K_{a3}}{[H^+]} + 1 + \frac{[H^+]}{K_{a1}}}\right)$<br>$E^\circ_{2,app} = E^\circ_5 + \frac{RT}{F} ln\left(\frac{\frac{K_{a6}}{[H^+]} + 1 + \frac{[H^+]}{K_{a5}}}{\frac{K_{a4}}{[H^+]} + 1 + \frac{[H^+]}{K_{a2}}}\right)$<br>$E^\circ_{avg} = \frac{E^\circ_{1,app} + E^\circ_{2,app}}{2}$	pK_a: acid dissociation constant at the logarithmic scale<br>K_a: acid dissociation constant<br>T: temperature<br>R: gas constant<br>E°: reduction potential<br>E°_{ref}: experimentally measured reduction potential of the reference compound<br>E°_{app}: apparent potential<br>E°_{avg}: average potential

Table S4 Details of the library used for the redox potential calculations

Molecule number	InChI code	Molecule name	2D representation	Type	Note
1	InChI=1S/C4H10O/c1-4(2,3)5/h5H,1-3H3	tert-butanol	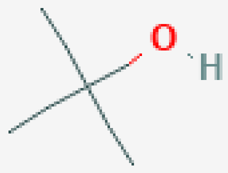	Linear oligo (ether carbonate)	formed by ring opening of EC
2	InChI=1S/C5H10O2/c1-3-4-7-5(2)6/h3-4H2,1-2H3	n-Propyl acetate	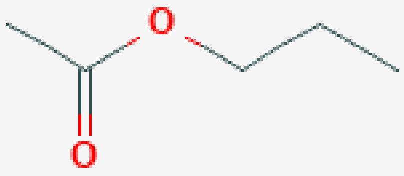	Solvent	Ester
3	InChI=1S/C8H18O3S/c1-3-5-7-11-12(9,10)8-6-4-2/h3-8H2,1-2H3	butyl sultone	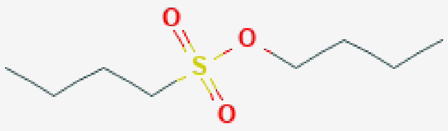	Electrolyte additive	SEI forming additive
4	InChI=1S/C3H6O3S/c4-7(5)3-1-2-6-7/h1-3H2	1,3-propane sultone	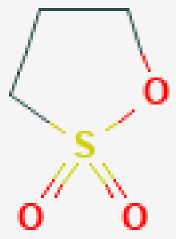	Electrolyte additive	Suppresses decomposition of solvents with graphite electrode
5	InChI=1S/C4H8O2S/c5-7(6)3-1-2-4-7/h1-4H2	tetra methylene sulfolane	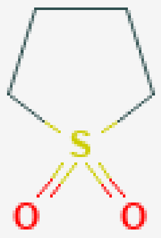	Electrolyte additive	
6	InChI=1S/C3H8O2S/c1-3-6(2,4)5/h3H2,1-2H3	ethyl methyl sulfone	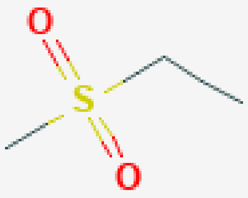	Electrolyte additive	
7	InChI=1S/C3H6O2S/c4-6(5)2-1-3-6/h1-3H2	tri-methylene sulfone	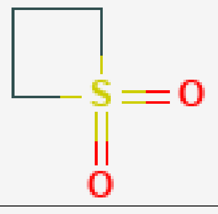	Electrolyte additive	Regulates Li deposition
8	InChI=1S/C6H14O2S/c1-4-6(3)9(7,8)5-2/h6H,4-5H2,1-3H3	ethyl sec-butyl sulfone	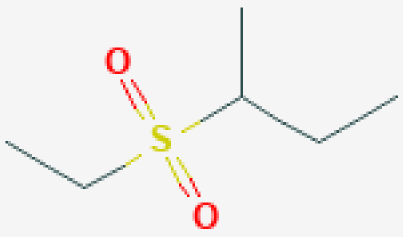	Electrolyte additive	Low boiling point and high conductivity
9	InChI=1S/C6H14O2S/c1-4-9(7,8)5-6(2)3/h6H,4-5H2,1-3H3	ethyl iso-butyl sulfone	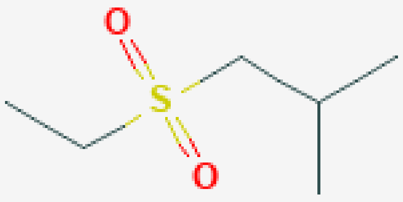	Electrolyte additive	Low melting point (< 0 °C)
10	InChI=1S/C5H12O2S/c1-4-8(6,7)5(2)3/h5H,4H2,1-3H3	ethyl iso-propyl sulfone	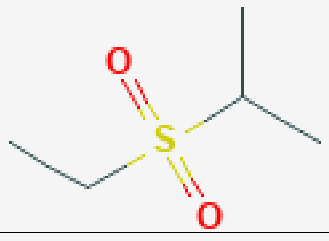	Electrolyte additive	Hydrolysis-resistant
11	InChI=1S/C7H7FO2S/c1-11(9,10)7-5-3-2-4-6(7)8/h2-5H,1H3	1-flouro-2-(methyl-sulfonyl) benzene	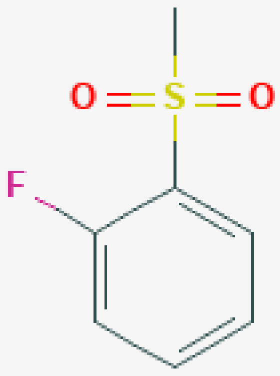	Electrolyte additive	

12	InChI=1S/C5H12O3S/c1-3-9(6,7)5-4-8-2/h3-5H2,1-2H3	ethyl 2-methoxyethyl sulfone	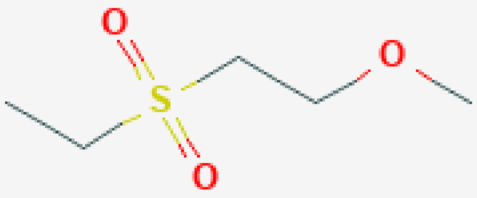	Electrolyte additive	
13	InChI=1S/C7H16O4S/c1-3-12(8,9)7-6-11-5-4-10-2/h3-7H2,1-2H3	ethyl methoxy ethoxy ethyl sulfone	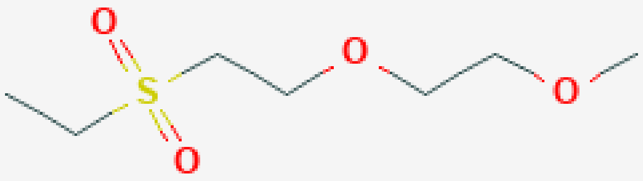	Electrolyte additive	Large oxidation potential
14	InChI=1S/C4H8O2/c1-3-6-4(2)5/h3H2,1-2H3	ethyl acetate	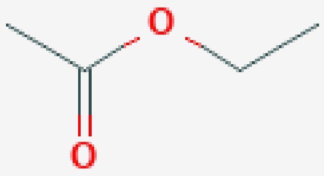	Solvent	Low viscosity and melting point compared to carbonates
15	InChI=1S/C3H6O2/c1-3(4)5-2/h1-2H3	methyl acetate	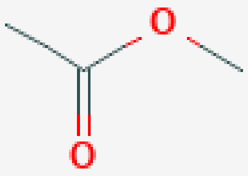	Solvent	Low viscosity and melting point compared to carbonates
16	InChI=1S/C2H4O2/c1-4-2-3/h2H,1H3	methyl formate	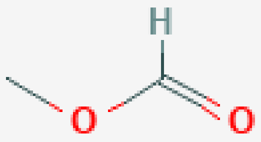	Solvent	Low viscosity and melting point compared to carbonates
17	InChI=1S/C5H10O2/c1-3-5(6)7-4-2/h3-4H2,1-2H3	ethyl propionate	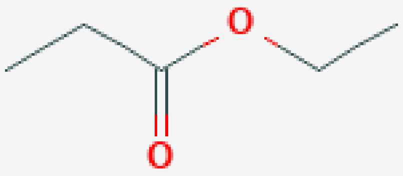	Solvent	
18	InChI=1S/C6H12O2/c1-3-5-6(7)8-4-2/h3-5H2,1-2H3	ethyl butyrate	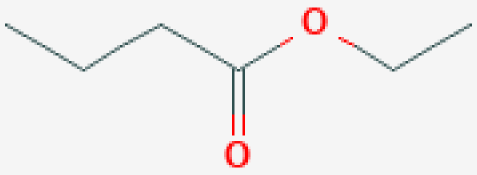	Solvent	
19	InChI=1S/C4H7FO2/c1-2-7-4(6)3-5/h2-3H2,1H3	ethyl fluoroacetate	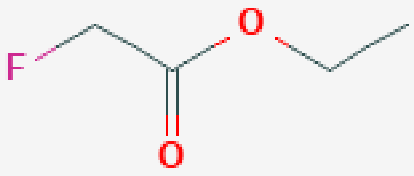	Solvent	Fluorinated
20	InChI=1S/C3H4F2O2/c1-7-3(6)2(4)5/h2H,1H3	methyl difluoro acetate	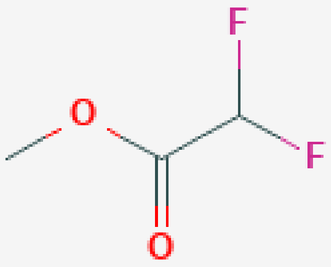	Solvent-Additive	Fluorinated
21	InChI=1S/C5H9FO2/c1-3-8-5(7)4(2)6/h4H,3H2,1-2H3	ethyl 2-fluoropropionate	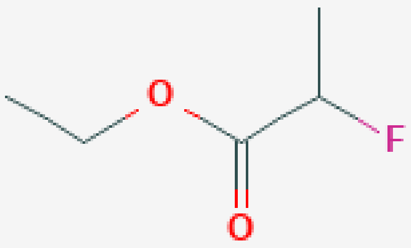	Additive	Fluorinated
22	InChI=1S/C5H9FO2/c1-2-5(7)8-4-3-6/h2-4H2,1H3	2-fluoroethyl propionate	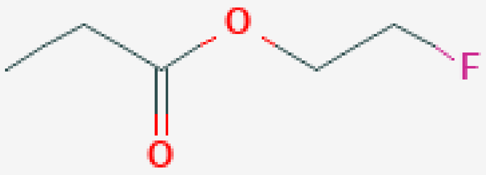	Additive	Fluorinated
23	InChI=1S/C5H9FO2/c1-2-8-5(7)3-4-6/h2-4H2,1H3	ethyl 3-fluoropropionate	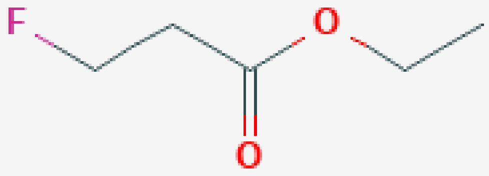	Additive	Fluorinated
24	InChI=1S/C5H10O3/c1-3-7-5(6)8-4-2/h3-4H2,1-2H3	diethyl carbonate	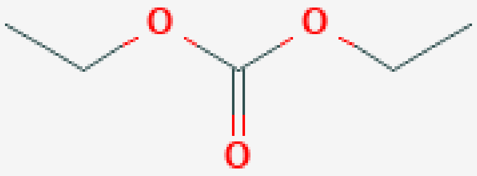	Solvent	
25	InChI=1S/C3H6O3/c1-5-3(4)6-2/h1-2H3	dimethyl carbonate	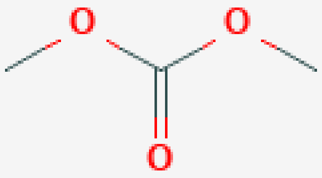	Solvent	

26	InChI=1S/C3H4O3/c4-3-5-1-2-6-3/h1-2H2	ethylene carbonate	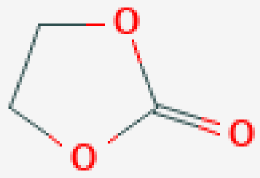	Solvent	
27	InChI=1S/C4H6O3/c1-3-2-6-4(5)7-3/h3H,2H2,1H3	propylene carbonate	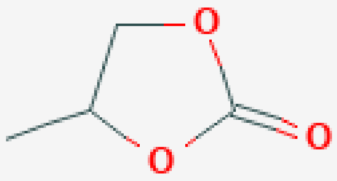	Solvent	
28	InChI=1S/C4H8O3/c1-3-7-4(5)6-2/h3H2,1-2H3	ethyl methyl carbonate	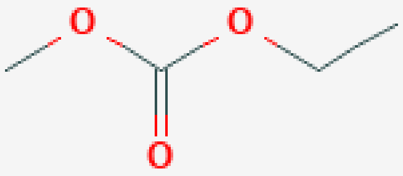	Solvent	
29	InChI=1S/C4H10O2/c1-5-3-4-6-2/h3-4H2,1-2H3	1,2-dimethoxyethane	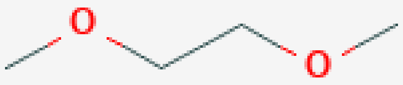	Solvent	
30	InChI=1S/C3H6O2/c1-2-5-3-4-1/h1-3H2	Dioxolane	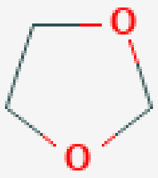	Solvent	
31	InChI=1S/C4H7FO2/c1-4(6)7-3-2-5/h2-3H2,1H3	Fluoroethyl acetate	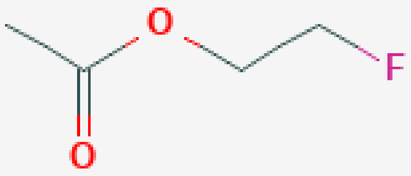	Solvent	
32	InChI=1S/C4H8O2/c1-4-5-2-3-6-4/h4H,2-3H2,1H3	2-methyl-1,3-dioxolane	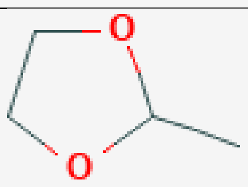	Product	oligomer of alkyl carbonates – formed upon oxidation of EC
33	InChI=1S/C5H8O4/c6-5-8-3-1-7-2-4-9-5/h1-4H2	1,3,6-trioxocan-2-one	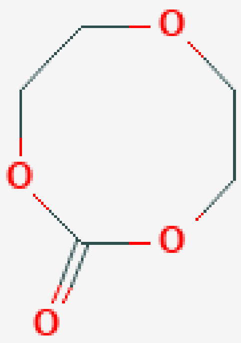	Product	oligomer of alkyl carbonates – formed upon oxidation of EC
34	InChI=1S/C5H10O/c1-5-3-2-4-6-5/h5H,2-4H2,1H3	methyl tetrahydrofuran	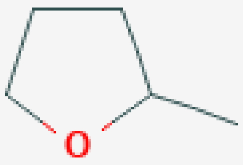	Solvent	
35	InChI=1S/C2H3N/c1-2-3/h1H3	acetonitrile	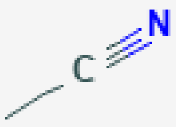	Solvent	
36	InChI=1S/C4H6O2/c5-4-2-1-3-6-4/h1-3H2	gamma-Butyrolactone	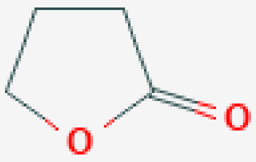	Solvent	
37	InChI=1S/C4H10O/c1-3-5-4-2/h3-4H2,1-2H3	diethyl ether	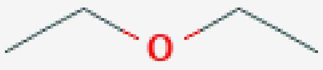	Additive	
38	InChI=1S/C2H6OS/c1-4(2)3/h1-2H3	dimethyl sulfoxide	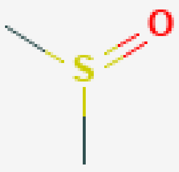	Solvent	
39	InChI=1S/C4H7NO2/c1-5-2-3-7-4(5)6/h2-3H2,1H3	3-methyl oxazolidin-2-one	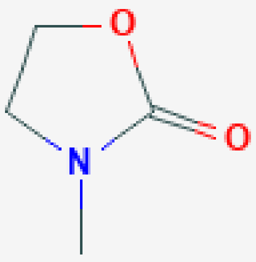	Solvent	

40	InChI=1S/C4H8O/c1-2-4-5-3-1/h1-4H2	tetrahydrofuran	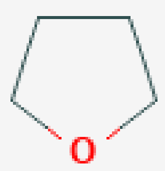	Solvent	
41	InChI=1S/C5H10O2S/c1-5-2-3-8(6,7)4-5/h5H,2-4H2,1H3	3-methyl sulfolane	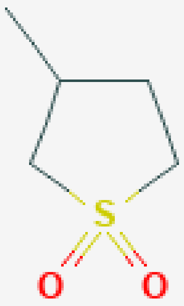	Solvent-additive	
42	InChI=1S/C4H8O2/c1-4-2-5-3-6-4/h4H,2-3H2,1H3	4-methyl dioxolane	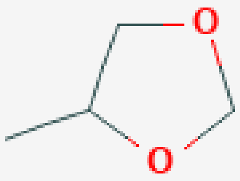	Solvent	
43	InChI=1S/C5H8O3/c1-2-4-3-7-5(6)8-4/h4H,2-3H2,1H3	1,2-butylene carbonate	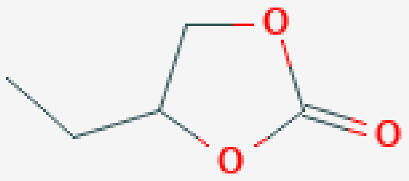	Solvent	
44	InChI=1S/C5H9ClO2/c1-2-3-5(7)8-4-6/h2-4H2,1H3	chloromethyl butyrate	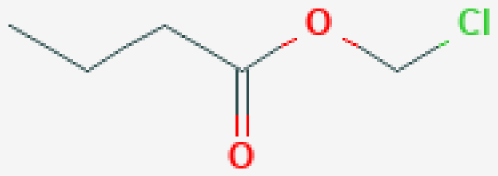	Solvent	
45	InChI=1S/C6H14O2/c1-3-7-5-6-8-4-2/h3-6H2,1-2H3	1,2-diethoxyethane	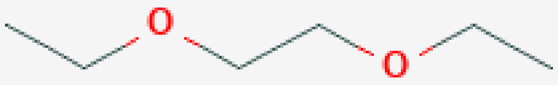	Solvent	
46	InChI=1S/C4H10S/c1-3-5-4-2/h3-4H2,1-2H3	diethyl sulfite	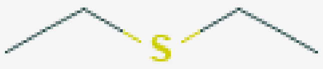	Additive	Similar in structure to organic carbonates; decomposition potential lower than 3.5 V
47	InChI=1S/C4H6F2O3/c1-8-4(7)9-2-3(5)6/h3H,2H2,1H3	difluoro ethyl methyl carbonate	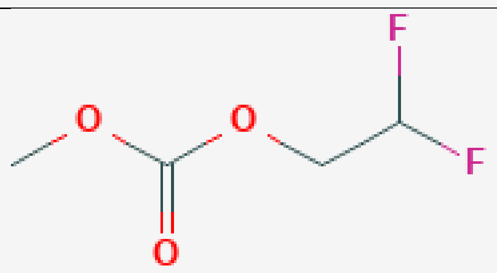	Solvent	Partially fluorinated alkyl carbonate
48	InChI=1S/C3H8O2/c1-4-3-5-2/h3H2,1-2H3	dimethoxymethane	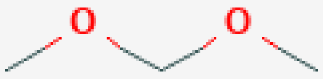	Solvent	
49	InChI=1S/C2H6S/c1-3-2/h1-2H3	dimethyl sulfide	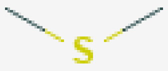	Solvent	Decomposition potential > 4.5 V;<br>Improves conductivity of electrolyte and capacity of the battery
50	InChI=1S/C5H8O2/c6-5-3-1-2-4-7-5/h1-4H2	δ-valero lacton	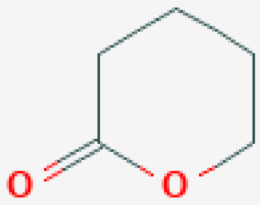	Solvent	Lactone
51	InChI=1S/C4H6F2O2/c1-2-8-4(7)3(5)6/h3H,2H2,1H3	ethyl difluoro acetate	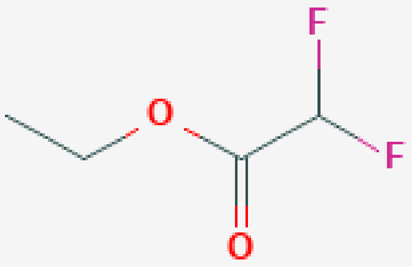	Solvent-Additive	Fluorinated
52	InChI=1S/C5H8F2O3/c1-2-9-5(8)10-3-4(6)7/h4H,2-3H2,1H3	ethyl difluoro ethyl carbonate	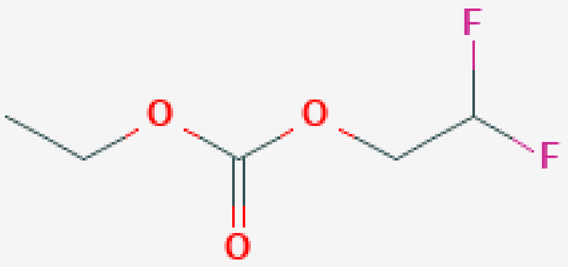	Solvent	

53	InChI=1S/C6H12F2O2/c1-2-9-3-4-10-5-6(7)8/h6H,2-5H2,1H3	ethoxy difluoro ethoxy ethane	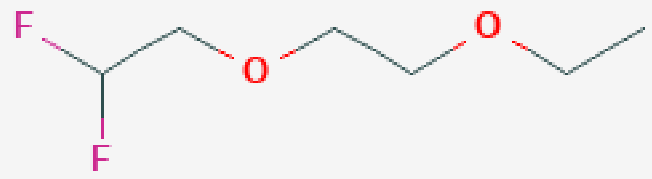	Solvent	Fluorinated
54	InChI=1S/C5H9FO3/c1-2-8-5(7)9-4-3-6/h2-4H2,1H3	ethyl fluoroethyl carbonate	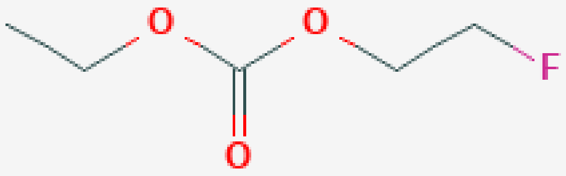	Solvent	Offers higher conductivity than diethyl carbonate
55	InChI=1S/C6H13FO2/c1-2-8-5-6-9-4-3-7/h2-6H2,1H3	ethoxy fluoro ethoxy ethane	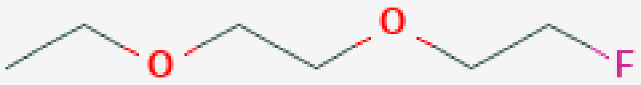	Solvent	Fluorinated
56	InChI=1S/C2H6O2/c3-1-2-4/h3-4H,1-2H2	ethylene glycol	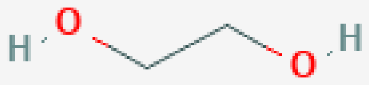	Filler material	Component of solid-state polymer electrolytes
57	InChI=1S/C3H8O2/c1-5-3-2-4/h4H,2-3H2,1H3	2-methoxyethanol	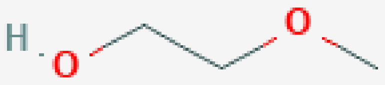	Solvent	
58	InChI=1S/C5H12O2/c1-3-7-5-4-6-2/h3-5H2,1-2H3	1-ethoxy-2-methoxyethane	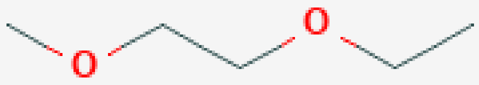	Solvent	High relative permittivity and lower viscosity compared to dimethyl carbonate
59	InChI=1S/C4H10O2S/c1-3-7(5,6)4-2/h3-4H2,1-2H3	ethyl methyl sulfone	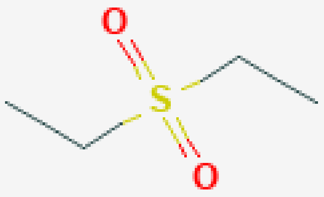	Solvent	Low melting point and high conductivity
60	InChI=1S/C2H4O/c1-2-3-1/h1-2H2	ethylene oxide	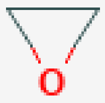	Polymer host	Polymer electrolyte material
61	InChI=1S/C6H12O3/c1-3-5-9-6(7)8-4-2/h3-5H2,1-2H3	ethyl propyl carbonate	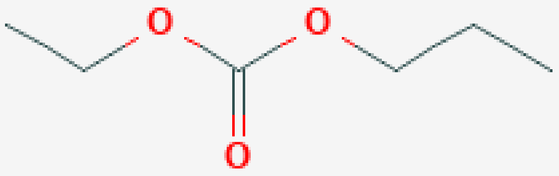	Solvent	
62	InChI=1S/C5H12O/c1-3-5-6-4-2/h3-5H2,1-2H3	ethyl propyl ether	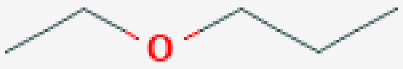	Solvent	
63	InChI=1S/C2H4O3S/c3-6-4-1-2-5-6/h1-2H2	ethylene sulfite	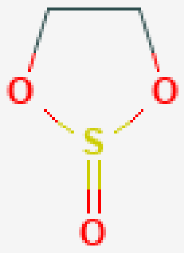	Additive	Film forming electrolyte additive
64	InChI=1S/C7H14O2S/c1-2-10(8,9)7-5-3-4-6-7/h7H,2-6H2,1H3	ethyl cyclopentyl sulfone	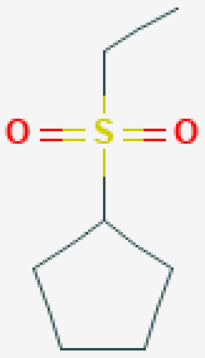	Solvent	High oxidation potential
65	InChI=1S/C5H7F3O3/c1-2-10-4(9)11-3-5(6,7)8/h2-3H2,1H3	ethyl trifluoro ethyl carbonate	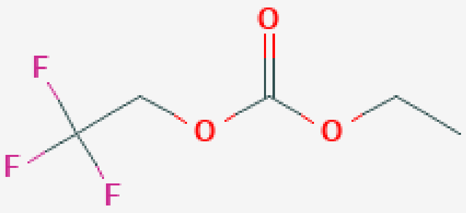	Solvent	Fluorinated; Good low temperature performance

66	InChI=1S/C6H6F9O4P/c7-4(8,9)1-17-20(16,18-2-5(10,11)12)19-3-6(13,14)15/h1-3H2	tris(2,2,2-trifluoroethyl) phosphate	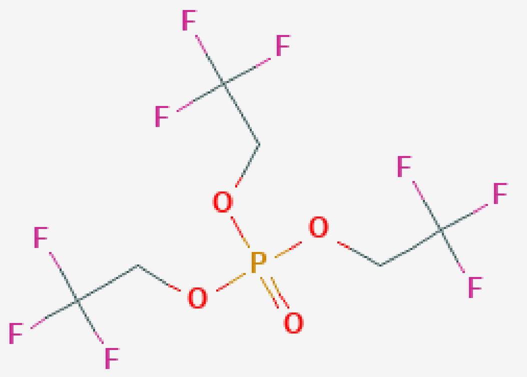	Co-solvent	Flame-retarding in EC-PC-EMC electrolytes
67	InChI=1S/C4H8O2S/c1-3-7(5,6)4-2/h3H,1,4H2,2H3	ethyl vinyl sulfone	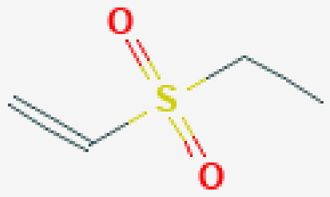	Solvent	High conductivity in LiTFSI electrolyte
68	InChI=1S/C3H3FO3/c4-2-1-6-3(5)7-2/h2H,1H2	fluoro ethylene carbonate	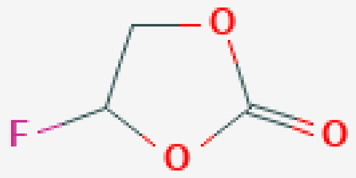	Solvent	Fluorinated; Forms a protective SEI layer on the graphite anode upon reduction
69	InChI=1S/C5H11FO2/c1-7-4-5-8-3-2-6/h2-5H2,1H3	fluoro ethoxy methoxy ethane	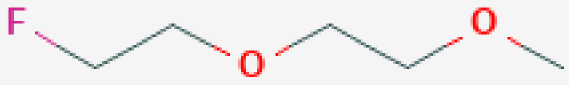	Solvent	Fluorinated, High donor number
70	InChI=1S/C4H5FO2/c5-3-1-2-7-4(3)6/h3H,1-2H2	fluoro-γ-butyrolactone	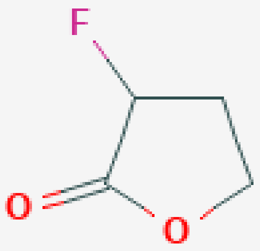	Additive	
71	InChI=1S/C4H5FO3/c5-1-3-2-7-4(6)8-3/h3H,1-2H2	fluoro propylene carbonate	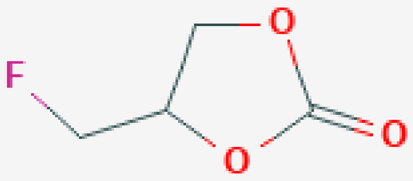	Solvent	Highly polar
72	InChI=1S/C5H9FO3/c1-8-5(7)9-4-2-3-6/h2-4H2,1H3	fluoro propyl methyl carbonate	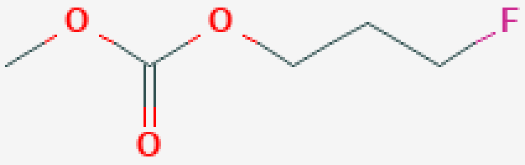	Solvent	
73	InChI=1S/C4H7F3O2S/c1-10(8,9)3-2-4(5,6)7/h2-3H2,1H3	trifluoro propyl methyl sulfone	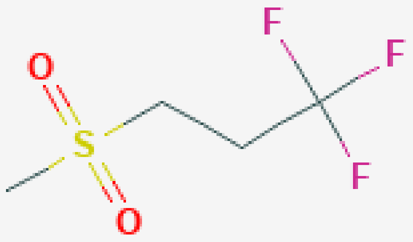	Solvent	High-voltage electrolyte
74	InChI=1S/C4H2O3/c5-3-1-2-4(6)7-3/h1-2H	maleic Anhydride	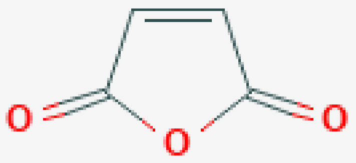	Additive	Film-forming additive used in gel-based electrolytes
75	InChI=1S/C5H10O2/c1-3-4-5(6)7-2/h3-4H2,1-2H3	methyl butyrate	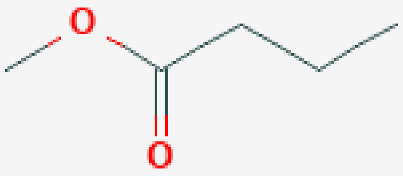	Cosolvent	
76	InChI=1S/C4H5NO2/c1-7-4(6)2-3-5/h2H2,1H3	methyl cyanoacetate	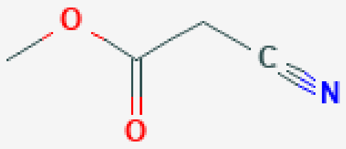	Additive	nucleophile
77	InChI=1S/C4H10O3S/c1-7-3-4-8(2,5)6/h3-4H2,1-2H3	methoxy ethyl methyl sulfone	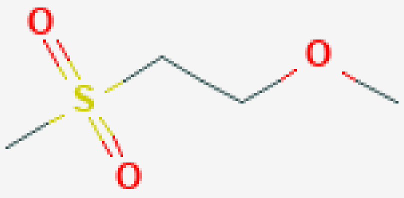	Solvent	
78	InChI=1S/C3H5FO3/c1-6-3(5)7-2-4/h2H2,1H3	mono fluoro dimethyl carbonate	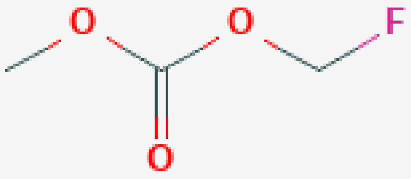	Solvent	Fluorinated, Higher conductivities than dimethyl carbonates at low concentrations

79	InChI=1S/C4H7FO3/c1-7-4(6)8-3-2-5/h2-3H2,1H3	Mono fluoro ethyl methyl carbonate	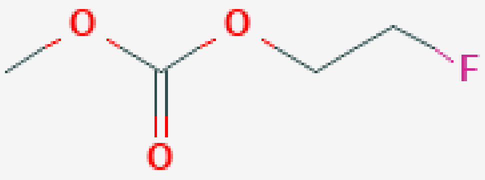	Solvent	Fluorinated
80	InChI=1S/C5H10O3/c1-4(2)8-5(6)7-3/h4H,1-3H3	methyl isopropyl carbonate	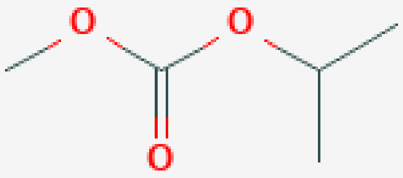	Solvent	Alkyl carbonate
81	InChI=1S/C5H10O3/c1-3-4-8-5(6)7-2/h3-4H2,1-2H3	methyl propyl carbonate	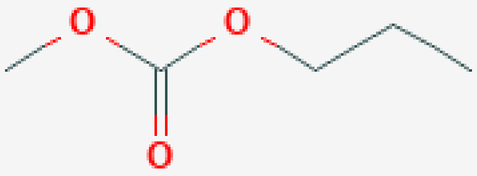	Solvent	
82	InChI=1S/C4H8O2S/c1-4-2-3-7(4,5)6/h4H,2-3H2,1H3	methyl tri methylene sulfone	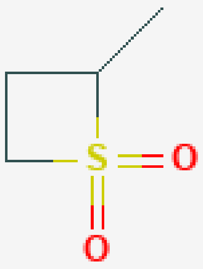	Solvent	
83	InChI=1S/C2H6O2S/c1-5(2,3)4/h1-2H3	dimethyl sulfone	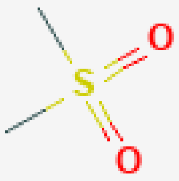	Solvent	Enhanced safety with ethylene carbonate due to the high flash point of the mixture
84	InChI=1S/C5H5F5O3/c1-12-3(11)13-2-4(6,7)5(8,9)10/h2H2,1H3	penta fluoro propyl methyl carbonate	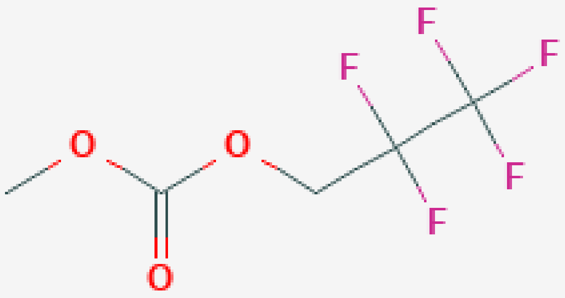	Solvent	Fluorinated; High molecular volume and molecular weight
85	InChI=1S/C3H6O3S/c1-3-2-5-7(4)6-3/h3H,2H2,1H3	propylene sulfite	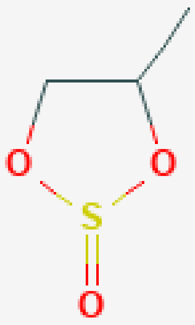	Additive	Film-forming
86	InChI=1S/C5H6F4O3/c1-11-4(10)12-2-5(8,9)3(6)7/h3H,2H2,1H3	tetra fluoro propyl methyl carbonate	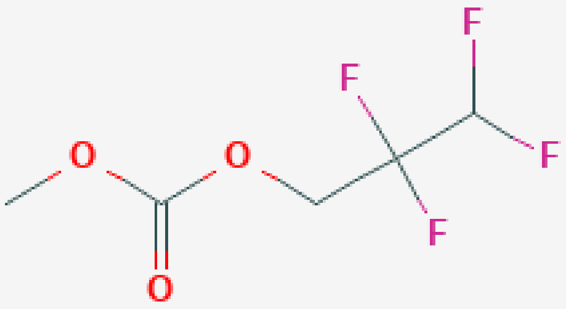	Solvent	Fluorinated
87	InChI=1S/C4H5F3O3/c1-9-3(8)10-2-4(5,6)7/h2H2,1H3	trifluoro ethyl methyl carbonate	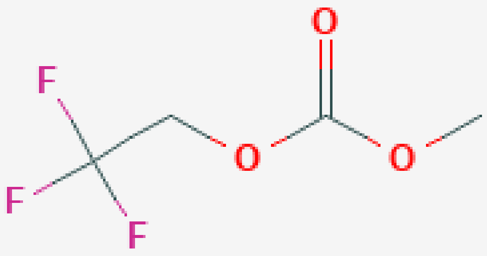	Solvent	Fluorinated
88	InChI=1S/C5H7F3O3/c1-10-4(9)11-3-2-5(6,7)8/h2-3H2,1H3	trifluoro propyl methyl carbonate	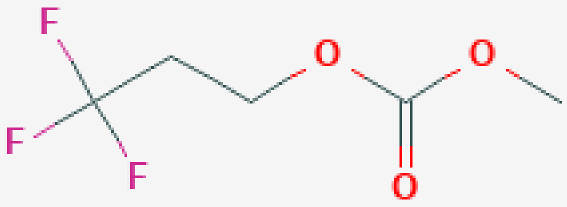	Solvent	Fluorinated
89	InChI=1S/C12H26O2S/c1-7-11(9(3)4)15(13,14)12(8-2)10(5)6/h9-12H,7-8H2,1-6H3	ethyl-iso-butyl sulfone	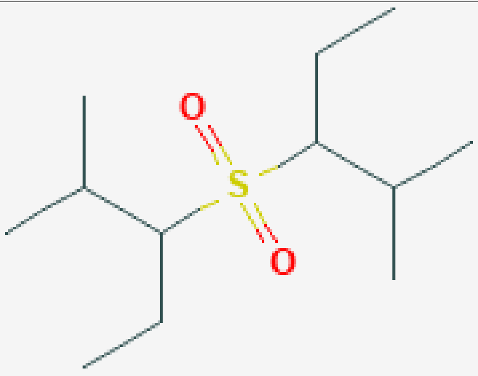	Solvent	

90	InChI=1S/C10H22O2S/c1-7-9(3,4)13(11,12)10(5,6)8-2/h7-8H2,1-6H3	ethyl-iso-propyl sulfone	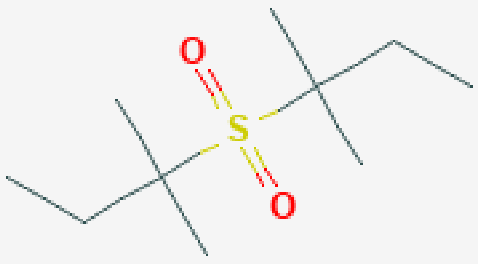	Solvent	
91	InChI=1S/C8H12F6O2S/c9-7(10,11)3-1-5-17(15,16)6-2-4-8(12,13)14/h1-6H2	3,3,3-trifluoropropyl methyl sulfone	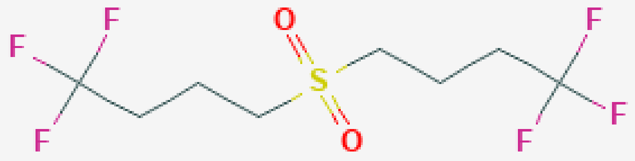	Solvent	
92	InChI=1S/C4H8O3/c1-6-3-4(5)7-2/h3H2,1-2H3	methyl methoxy acetate	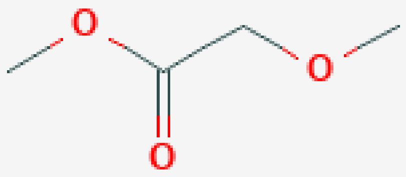	Solvent	Improvement in safety compared to ethylene carbonate
93	InChI=1S/C6H12O3/c1-5(4-8-3)9-6(2)7/h5H,4H2,1-3H3	methoxy-2-propyl acetate	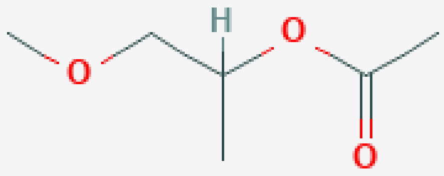	Solvent	
94	InChI=1S/C3H5FO2/c1-6-3(5)2-4/h2H2,1H3	methyl fluoro acetate	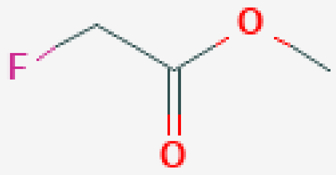	Solvent	Fluorinated
95	InChI=1S/C5H12O2/c1-3-6-5-7-4-2/h3-5H2,1-2H3	ethoxy methoxy ethane	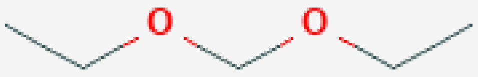	Solvent	Organic
96	InChI=1S/C2H6O3S/c1-4-6(3)5-2/h1-2H3	dimethyl sulfite	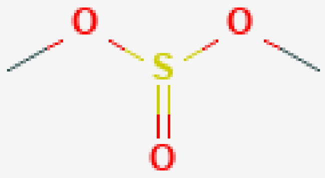	Solvent	
97	InChI=1S/C2H6O3S/c1-5-6(2,3)4/h1-2H3	methyl methane sulfonate	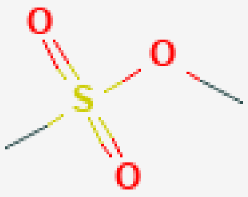	Solvent	
98	InChI=1S/C2H6O4S/c1-5-7(3,4)6-2/h1-2H3	dimethyl sulfate	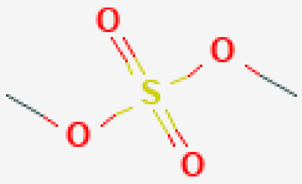	Solvent	
99	InChI=1S/C4H8O2S/c1-4-2-7(5,6)3-4/h4H,2-3H2,1H3	2-methyltrimethylene sulfone	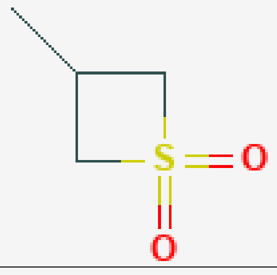	Solvent	
100	InChI=1S/C5H8O2/c1-4-2-3-5(6)7-4/h4H,2-3H2,1H3	γ-valero lactone	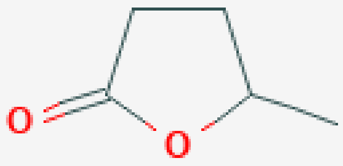	Solvent	Lactone

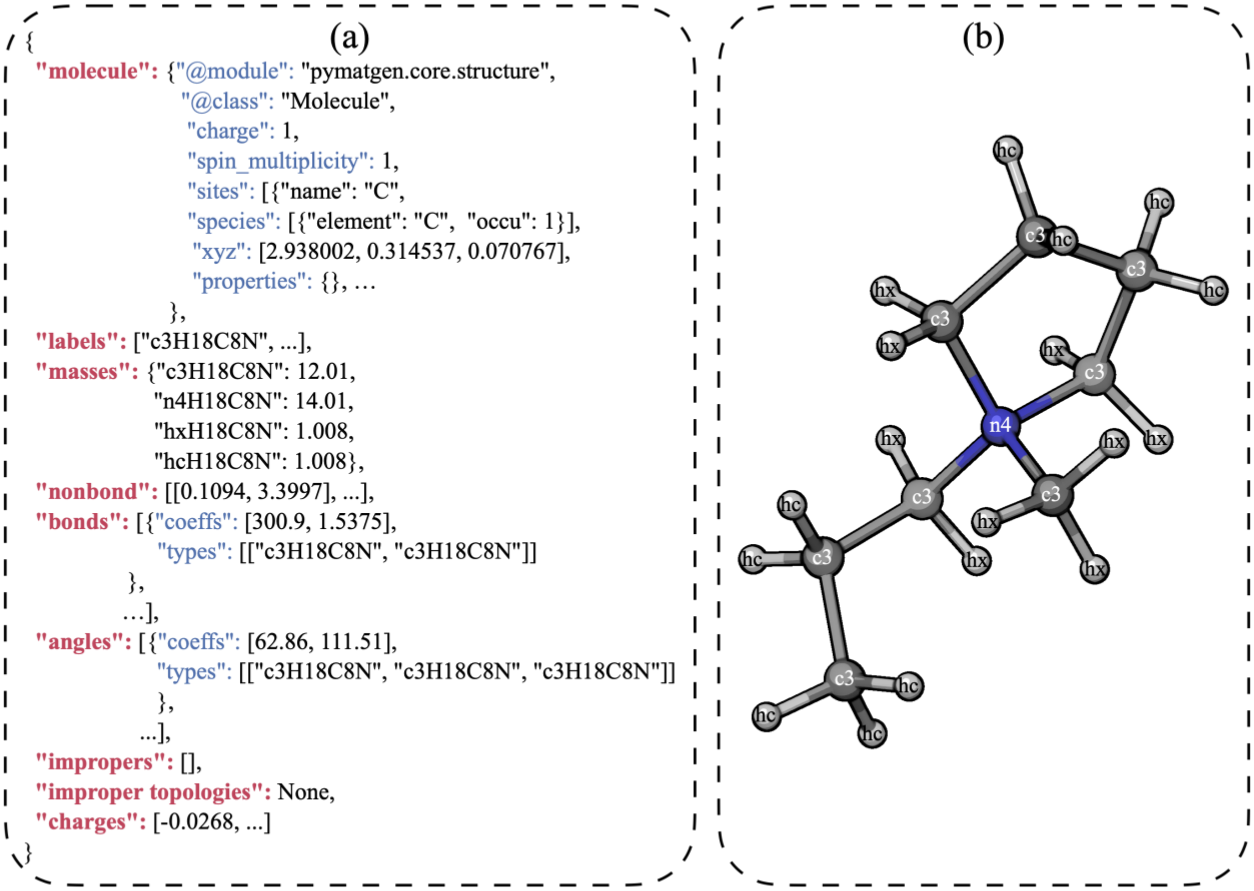

Fig. S4 Example of a (a) force field dictionary and (b) the labeled structure of the corresponding molecule

(a)

System	Solvent	Donor number	Structure
1	Triglyme	14.0	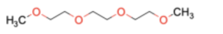
2	Acetonitrile	14.1	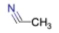
3	Ethyl methyl carbonate	15.1	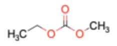
4	Propylene carbonate	16.4	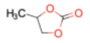
5	Butyrolactone	16.6	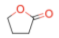
6	Tetraglyme	17.0	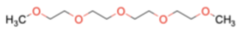
7	Ethylene carbonate	18.0	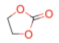
8	Diglyme	20.0	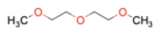
9	Monoglyme	24.0	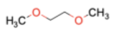
10	Water	33.0	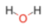

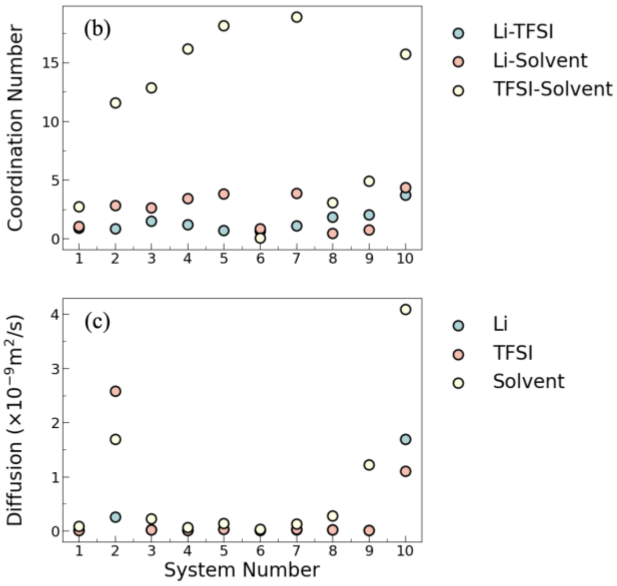

Fig. S5 (a) Solvents used for MD simulations with 1 M lithium bis(trifluoromethylsulfonyl)imide, (b) coordination number, and (c) diffusion coefficients computed using MISPR

```python
from collections import OrderedDict

import numpy as np

from pymatgen.core.structure import Molecule

from fireworks import LaunchPad

from mispr.hybrid.workflows.core import run_hybrid_calcs


lpad = LaunchPad.auto_load()

mol_names = ["dme", "tfsi", "li"]
li_mol = Molecule.from_file("li.xyz")
li_mol.set_charge_and_spin(1, 1)
li_param = {
    "Labels": ["li"],
    "Masses": OrderedDict({"li": 6.941}),
    "Nonbond": [[0.191, 1.46]],
    "Bonds": [],
    "Angles": [],
    "Dihedrals": [],
    "Impropers": [],
    "Improper Topologies": None,
    "Charges": np.asarray([1.0]),
}

wf = run_hybrid_calcs(
    mol_operation_type=["get_from_file", "get_from_file", "get_from_mol"],
    mol=["dme.xyz", "tfsi.xyz", li_mol],
    mol_type=["Solvents", "Solutes", "Solutes"],
    mol_data=[648, 36, 36],
    box_data=50.0,
    mol_name=mol_names,
    ff_method=["get_from_esp", "get_from_esp", "get_from_dict"],
    ff_params=[{}, {}, li_param],
    skips=[None, None, ["opt"]],
    charge=[0, -1, 1],
    data_file_name="data.mixture",
    analysis_list=["diffusion", "rdf", "cn", "clusters"],
    analysis_settings=[
        {
            "diff_names": mol_names,
            "timestep": 1,
            "com_drift": True,
            "msd_method": "from_dump",
            "dump_freq": 100,
            "diff_dist": True,
            "avg_interval": True,
        },
        {"rdf_type": "atomic"},
        {"cn_type": "atomic"},
        {"atom_type": 10, "r_cut": 2.25},
    ],
    process_mol_func=False
)

lpad.add_wf(wf)
```

Fig. S6 Code snippet of the submission of a hybrid DFT-MD workflow for a lithium bis(trifluoromethylsulfonyl)imide in monoglyme liquid solution using the default MD recipe in MISPR, GAFF parameters for the anion and solvent, and user-defined parameters for the cation

(a)


```
{
  "_id": {"$oid": "5f7231b69f337f2f00735287"},
  "molecule": {"@module": "pymatgen.core.structure",
               "@class": "Molecule",
               "charge": 0,
               "spin_multiplicity": 1,
               "sites": [...]},
  "smiles": "[S]SSS[Li].[Li].C1=Cc2cccc3c2[C@](C1)(CC=C3)O",
  "inchi": "InChI=1S/C13H12O.2Li.HS8/c14-13-8-2-6-10-4-1-5-11
           (12(10)13)7-3-9-13;;;1-3-5-7-8-6-4-2/h1-7,14H,8-9H2;;;
           1H/q;;+1;/p-1",
  "formula_pretty": "Li2H12C13S4O",
  "chemsys": "C-H-Li-O-S",
  "energy": -2185.8227,
  "be_eV": {"sites": [4,19],
            "atoms": ["Li", "O"],
            "value": -0.3175},
  "functional": "B3LYP",
  "basis": "6-31G(d)",
  "phase": "gas",
  "tag": {"project: "Li-S"},
  "state": "successful",
  "wall_time (s)": 779.6842,
  "last_updated": {"$date": "2020-09-28T18:55:49.768Z"},
  "version": "0.0.1",
  "gauss_version": "ES64L-G16RevC.01",
  "functional_group": "hydroxyl",
  "polysulfide": "Li2S4",
  "run_ids": […]
}
```


(b)


```
{
  "smiles": ["O(C)CCOC",
             "[N](S(=O)(=O)C(F)(F)F)S(=O(=O)C(F (F)F",
             "[Li]"],
  "nmols": [648, 36, 36],
  "natoms_per_mol": [16, 15, 1],
  "mass": [16, 12.01, 12.01, 1.008, 14.01, 32.06, 16.0, 12.01, 19.0, 6.94],
  "box": {"@module": "pymatgen.io.lammps.data",
          "@class": "LammpsBox",
          "@version": null,
          "bounds": [[-0.25, 50.25], [-0.25, 50.25], [-0.25, 50.25]],
          "tilt": null},
  "diffusion": {"timestep": 1, "units": "real", "tao_coeff": 4,
                "msd_type": "com", "return_all": false,
                "com_drift": true, …,
                "diffusion": [1.237e-09, 5.769e-10, 5.888e-10],
                "std": [8.038e-13, 1.469e-12, 2.006e-12],
                "r2": [0.999, 0.993, 0.988]},
  "rdf": {"r_cut": 20, "bin_size": [0.05], …,
          "rdf_type": "atomic", "rdf_use_default_atom_ids": true},
  "cn": {"r_cut": [2.7, 9.82, 7.26, 10.27],
         "bin_size": [0.05], …,
         "partial_relations": [[10, 10, 10, 10], [1, 5, 7, 10]],
         "cn_type": "atomic", "cn_use_default_atom_ids": true,
         "cn": {"cn_10-1": 5.253, "cn_10-5": 1.828,
                "cn_10-7": 2.652, "cn_10-10": 0.487}},
  "clusters": {"atom_type": 10, "r_cut": 2.7, …,
               "num_clusters": 36036, "num_configurations": 8, …},
  "run_ids": […]
}
```


Fig. S7 Example of (a) binding energy document from the DFT database and (b) MD document

Table S5 Details of the library used for the NMR calculations along with the experimental measurements made in chloroform solvent at 298 K and referenced to tetramethylsilane

Molecule number	InChI code	Molecule name	2D representation	^{13}C NMR spectra frequency (MHz)	^{1}H NMR spectra frequency (MHz)	^{13}C NMR chemical shift (ppm)	^{1}H NMR chemical shift (ppm)	Ref
1	InChI=1S/H2O/h1H2	water	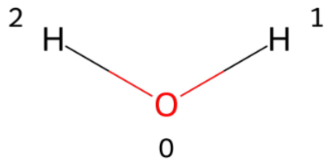		75.5, 126, 151		1,2: 1.56	[2]
2	InChI=1S/C2H4O2/c 1-2(3)4/h1H3,(H,3,4)	acetic acid	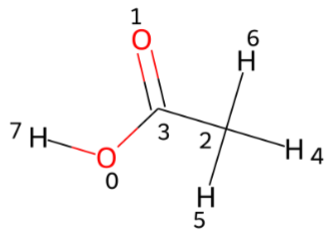	300, 500, 600	75.5, 126, 151	3: 175.99	4,5,6: 2.10	[2]
3	InChI=1S/C3H6O/c1 -3(2)4/h1-2H3	acetone	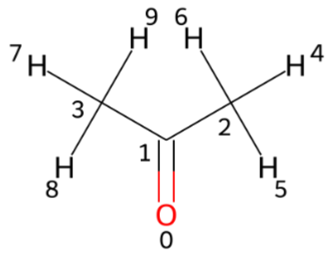	300, 500, 600	75.5, 126, 151	1: 207.07<br>2,3: 30.92	4,5,6,7,8,9: 2.17	[2]
4	InChI=1S/C2H3N/c1 -2-3/h1H3	acetonitrile	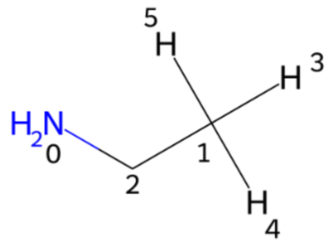	300, 500, 600	75.5, 126, 151	1: 1.89<br>2: 116.43	3,4,5: 2.10	[2]
5	InChI=1S/C6H6/c1- 2-4-6-5-3-1/h1-6H	benzene	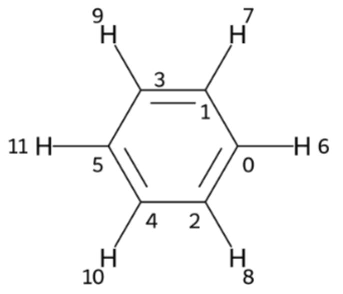	300, 500, 600	75.5, 126, 151	0,1,2,3,4,5: 128.37	6,7,8,9,10,11: 7.36	[2]
6	InChI=1S/C4H10O/c 1-4(2,3)5/h5H,1-3H3	tert -butyl alcohol	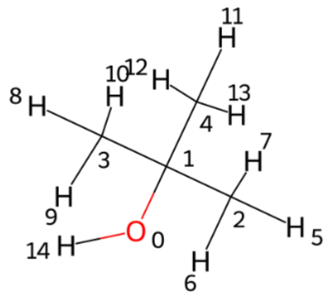	25.16	300	2,3,4: 31.23<br>1: 69.05	5,6,7,8,9,10,11,12,1 3: 1.262<br>14: 2.01	[3]
7	InChI=1S/CO2/c2-1-3	carbon dioxide	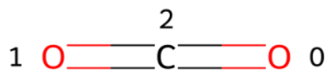	300, 500, 600	75.5, 126, 151	2: 124.99		[2]
8	InChI=1S/CS2/c2-1-3	carbon disulfide	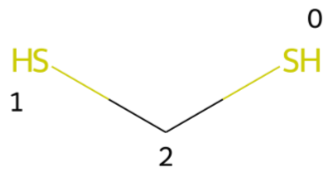	300, 500, 600	75.5, 126, 151	2: 192.83		[2]
9	InChI=1S/CCl4/c2-1(3,4)5	carbon tetrachloride	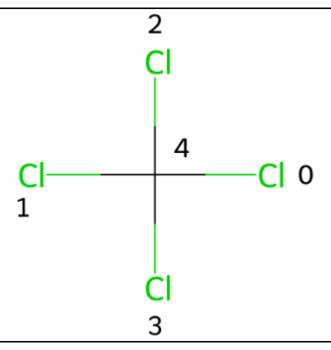	300, 500, 600	75.5, 126, 151	4: 96.34		[2]

10	InChI=1S/CHCl3/c2-1(3)4/h1H	chloroform	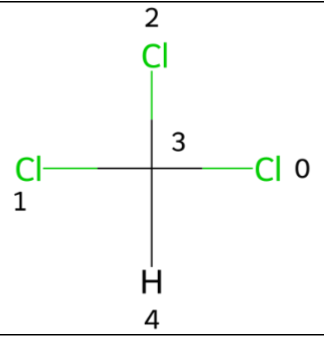	300, 500, 600	75.5, 126, 151	3: 77.36	4: 7.26	[2]
11	InChI=1S/C12H24O6/c1-2-14-5-6-16-9-10-18-12-11-17-8-7-15-4-3-13-1/h1-12H2	18-crown-6	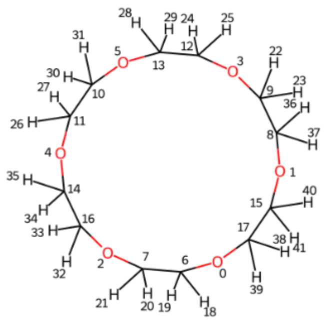	300, 500, 600	75.5, 126, 151	6,7,8,9,10,11,12,13,14,15,16,17: 70.55	18,19,20,21,22,23,24,25,26,27,28,29,30,31,32,33,34,35,36,37,38,39,40,41: 3.67	[2]
12	InChI=1S/C6H12/c1-2-4-6-5-3-1/h1-6H2	cyclohexane	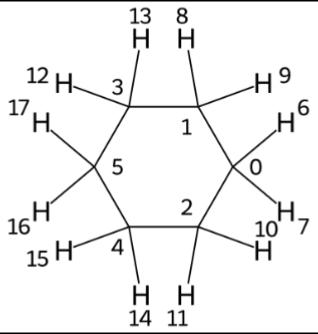	300, 500, 600	75.5, 126, 151	0,1,2,3,4,5: 26.94	6,7,8,9,10,11,12,13,14,15,16,17: 1.43	[2]
13	InChI=1S/C2H4Cl2/c3-1-2-4/h1-2H2	1,2-dichloroethane	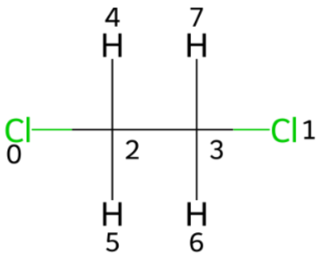	300, 500, 600	75.5, 126, 151	2,3: 43.5	4,5,6,7: 3.73	[2]
14	InChI=1S/CH2Cl2/c2-1-3/h1H2	dichloro-methane	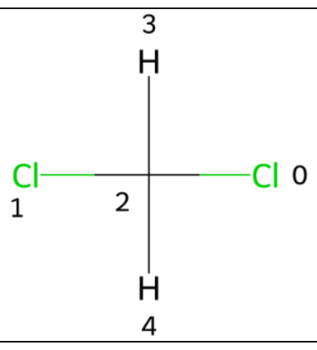	300, 500, 600	75.5, 126, 151	2: 53.52	3,4: 5.30	[2]
15	InChI=1S/C4H10O/c1-3-5-4-2/h3-4H2,1-2H3	diethyl ether	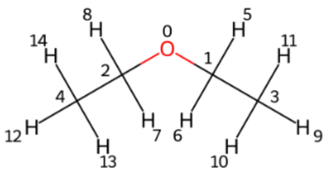	300, 500, 600	75.5, 126, 151	1,2: 65.91<br>3,4: 15.2	5,6,7,8: 3.48<br>9,10,11,12,13,14: 1.2	[2]
16	InChI=1S/C6H14O3/c1-7-3-5-9-6-4-8-2/h3-6H2,1-2H3	diglyme	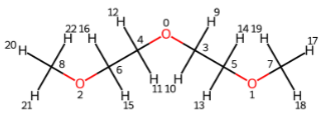	300, 500, 600	75.5, 126, 151	3,4,5,6: 70.51<br>7,8: 59.01	17,18,19,20,21,22: 3.65<br>9,10,11,12,13,14,15,16: 3.57	[2]
17	InChI=1S/C3H7NO/c1-4(2)3-5/h3H,1-2H3	Dimethyl-formamide	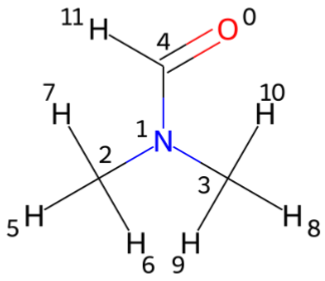	300, 500, 600	75.5, 126, 151	2: 36.5<br>3: 31.45<br>4: 162.62	5,6,7: 2.96<br>8,9,10: 2.88<br>11: 8.02	[2]
18	InChI=1S/C4H8O2/c1-2-6-4-3-5-1/h1-4H2	1,4-dioxane	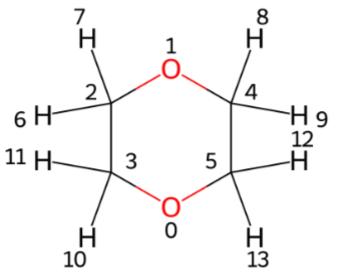	300, 500, 600	75.5, 126, 151	2,3,4,5: 67.14	6,7,8,9,10,11,12,13: 3.71	[2]

19	InChI=1S/C4H10O2/c1-5-3-4-6-2/h3-4H2,1-2H3	1,2-dimethoxy ethane	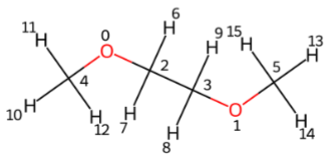	300, 500, 600	75.5, 126, 151	2,3: 71.84<br>4,5: 59.08	6,7,8,9: 3.55<br>10,11,12,13,14,15: 3.40	[2]
20	InChI=1S/C2H6/c1-2/h1-2H3	ethane	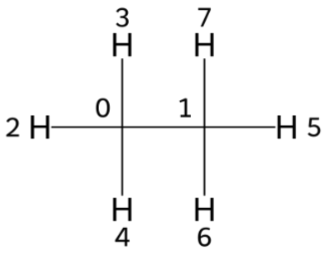	300, 500, 600	75.5, 126, 151	0,1: 6.89	2,3,4,5,6,7: 0.87	[2]
21	InChI=1S/C2H6O/c1-2-3/h3H,2H2,1H3	ethanol	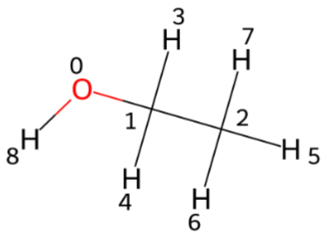	300, 500, 600	75.5, 126, 151	1: 58.28<br>2: 18.41	3,4: 3.66<br>5,6,7: 1.19<br>8: 1.33	[2]
22	InChI=1S/C4H8O2/c1-3-6-4(2)5/h3H2,1-2H3	ethyl acetate	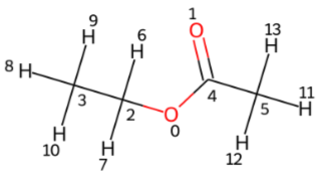	300, 500, 600	75.5, 126, 151	2: 60.49<br>3: 14.19<br>4: 171.36<br>5: 21.04	6,7: 4.12<br>8,9,10: 1.26<br>11,12,13: 2.05	[2]
23	InChI=1S/C2H4/c1-2/h1-2H2	ethylene	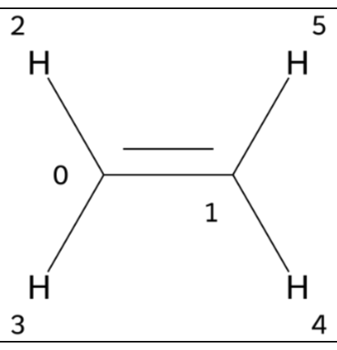	300, 500, 600	75.5, 126, 151	0,1: 123.13	2,3,4,5: 5.4	[2]
24	InChI=1S/C2H6O2/c3-1-2-4/h3-4H,1-2H2	ethylene glycol	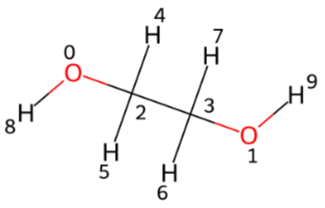	300, 500, 600	75.5, 126, 151	2,3: 63.79	4,5,6,7: 3.76	[2]
25	InChI=1S/C12H18/c1-7-8(2)10(4)12(6)11(5)9(7)3/h1-6H3	hexamethyl benzene	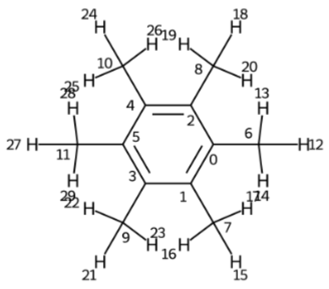	300, 500, 600	75.5, 126, 151	0,1,2,3,4: 132.21<br>6,7,8,9,10,11: 16.98	12,13,14,15,16,17,18,19,20,21,22,23,24,25,26,27,28,29: 2.24	[2]
26	InChI=1S/C6H14/c1-3-5-6-4-2/h3-6H2,1-2H3	n-hexane	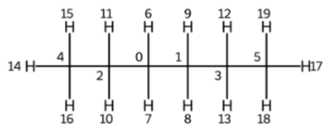	300, 500, 600	75.5, 126, 151	0,1: 31.64<br>2,3: 22.70<br>4,5: 14.14	14,15,16,17,18,19: 0.89<br>6,7,8,9,10,11,12,13: 1.27	[2]
27	InChI=1S/C6H18OSi2/c1-8(2,3)7-9(4,5)6/h1-6H3	hexamethyl disiloxane	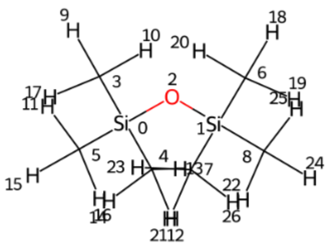	300, 500, 600	75.5, 126, 151	3,5,6,8: 1.97	9,10,11,15,16,17,18,19,20 ,24,25,26: 0.07	[2]

28	InChI=1S/C6H18N3OP/c1-7(2)11(10,8(3)4)9(5)6/h1-6H3	hexamethyl phosphoramide	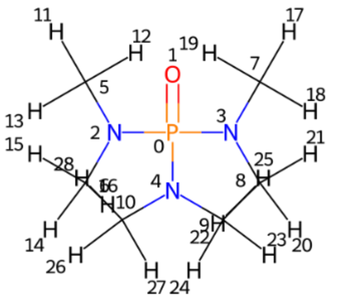	300, 500, 600	75.5, 126, 151	5,6,7,8,9,10: 36.87	11,12,13,14,15,16,17,18,19,20,21,22,23,24,25,26,27,28: 2.65	[2]
29	InChI=1S/H2/h1H	hydrogen	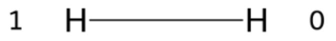	300, 500, 600	75.5, 126, 151		0,1: 4.62	[2]
30	InChI=1S/C3H4N2/c1-2-5-3-4-1/h1-3H,(H,4,5)	imidazole	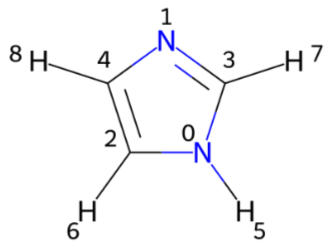	300, 500, 600	75.5, 126, 151	2,4: 122.0<br>3: 135.38	6,8: 7.10<br>7: 7.67	[2]
31	InChI=1S/CH4/h1H4	methane	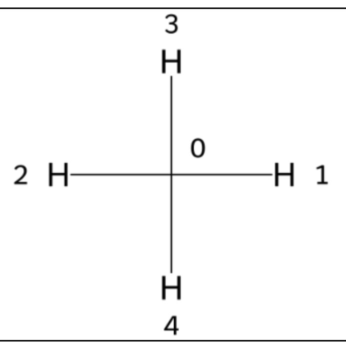	300, 500, 600	75.5, 126, 151	0: -4.63	1,2,3,4: 0.22	[2]
32	InChI=1S/CH4O/c1-2/h2H,1H3	methanol	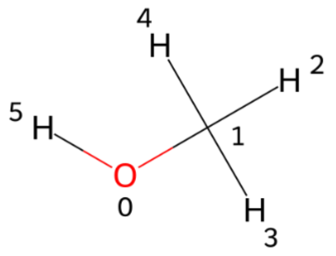	300, 500, 600	75.5, 126, 151	1: 50.41	2,3,4: 3.49<br>5: 1.09	[2]
33	InChI=1S/CH3NO2/c1-2(3)4/h1H3	nitromethane	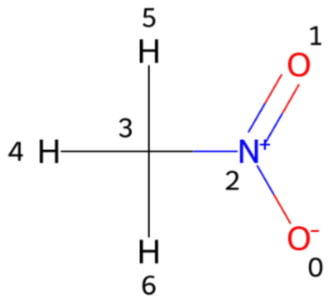	300, 500, 600	75.5, 126, 151	3: 62.50	4,5,6: 4.33	[2]
34	InChI=1S/C5H12/c1-3-5-4-2/h3-5H2,1-2H3	n-pentane	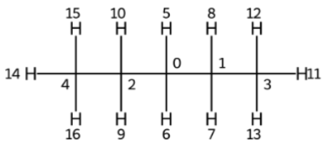	300, 500, 600	75.5, 126, 151	0: 34.16<br>1,2: 22.38<br>3,4: 14.08	5,6,7,8,9,10: 1.27<br>11,12,13,14,15,16: 0.88	[2]
35	InChI=1S/C3H8/c1-3-2/h3H2,1-2H3	propane	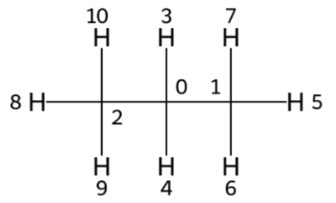	300, 500, 600	75.5, 126, 151	0: 16.37<br>1,2: 16.63	3,4: 1.32<br>5,6,7,8,9,10: 0.90	[2]
36	InChI=1S/C3H8O/c1-3(2)4/h3-4H,1-2H3	2-propanol	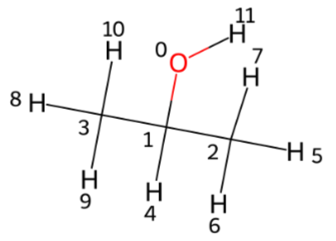	15.09	75.5, 126, 151	1: 64.04<br>2,3: 25.29	4: 4.008<br>5,6,7,8,9,10: 1.2<br>11: 2.16	[3]

37	InChI=1S/C3H6/c1-3-2/h3H,1H2,2H3	propylene	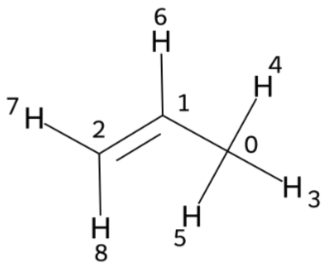	300, 500, 600	75.5, 126, 151	0: 19.50<br>1: 133.91<br>2: 115.74	3,4,5: 1.73<br>6: 5.83<br>7: 4.94<br>8: 5.03	[2]
38	InChI=1S/C5H5N/c1-2-4-6-5-3-1/h1-5H	pyridine	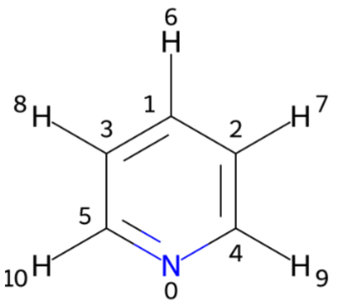	300, 500, 600	75.5, 126, 151	1: 135.96<br>2,3: 123.75<br>4,5: 149.90	6: 7.68<br>7,8: 7.29<br>9,10: 8.62	[2]
39	InChI=1S/C4H5N/c1-2-4-5-3-1/h1-5H	pyrrole	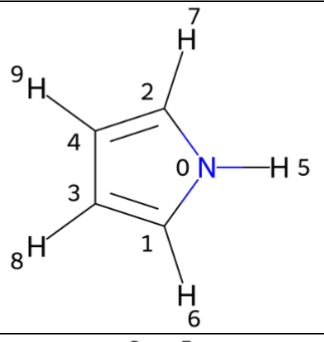	300, 500, 600	75.5, 126, 151	1,2: 117.77<br>3,4: 107.98	5: 8.40<br>6,7: 6.83<br>8,9: 6.26	[2]
40	InChI=1S/C4H9N/c1-2-4-5-3-1/h5H,1-4H2	pyrrolidine	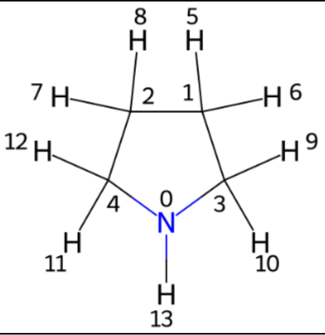	300, 500, 600	75.5, 126, 151	1,2: 25.56<br>3,4: 46.93	5,6,7,8: 1.68<br>9,10,11,12: 2.87	[2]
41	InChI=1S/C4H8O/c1-2-4-5-3-1/h1-4H2	tetrahydrofuran	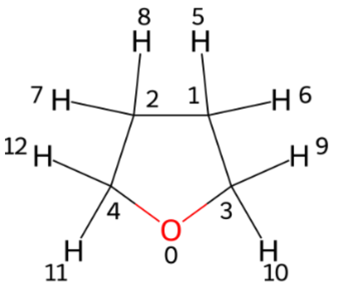	300, 500, 600	75.5, 126, 151	1,2: 25.62<br>3,4: 67.97	5,6,7,8: 1.85<br>9,10,11,12: 3.76	[2]
42	InChI=1S/C7H8/c1-7-5-3-2-4-6-7/h2-6H,1H3	toluene	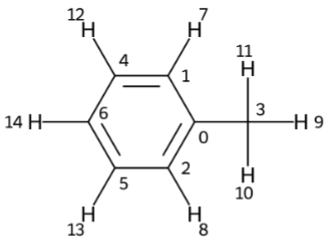	25.16	89.56	0: 137.83<br>1,2: 129.09<br>3: 21.41<br>4,5: 128.28<br>6: 125.38	7,8,12,13,14:<br>7.38-7.0<br>9,10,11: 2.34	[3]
43	InChI=1S/C6H15N/c1-4-7(5-2)6-3/h4-6H2,1-3H3	triethylamine	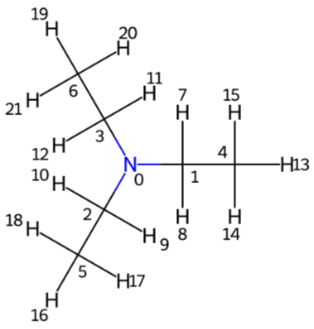	15.09	300	1,2,3: 46.46<br>4,5,6: 11.78	7,8: 2.425<br>13,14,15: 0.97	[3]
44	InChI=1S/C2H3NS/c1-3-2-4/h1H3	methyl isothiocyanate	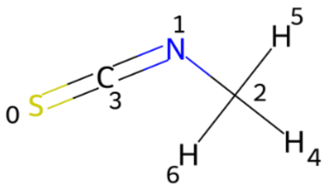	50.18	300	2: 30.48<br>3: 128.9	4,5,6: 3.325	[3]
45	InChI=1S/C4H2O3/c5-3-1-2-4(6)7-3/h1-2H	maleic anhydride	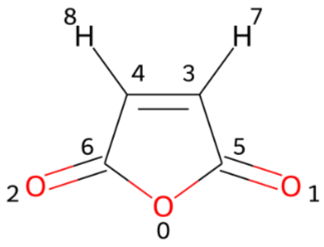	25.16	300	3,4: 136.76<br>5,6: 164.58	7,8: 7.048	[3]

46	InChI=1S/C5H6N2/c1-5-4-6-2-3-7-5/h2-4H,1H3	2-methylpyrazine	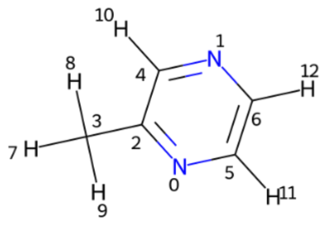	25.16	89.56	2: 154.11<br>3: 21.53<br>4: 144.85<br>5: 141.90<br>6: 143.90	7,8,9: 2.572<br>10: 8.38<br>11,12: 8.5-8.42	[3]
47	InChI=1S/C3H7N/c1-2-3-4/h2H,1,3-4H2	allylamine	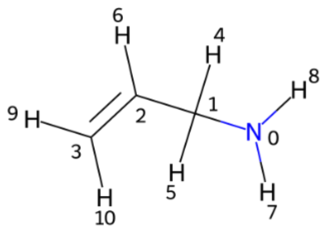	25.16	89.56	1: 44.82<br>2: 140.01<br>3: 113.48	4,5: 3.311<br>6: 5.954<br>7,8: 1.29<br>9: 5.051<br>10: 5.159	[3]
48	InChI=1S/C2H4O/c1-2-3/h2H,1H3	acetaldehyde	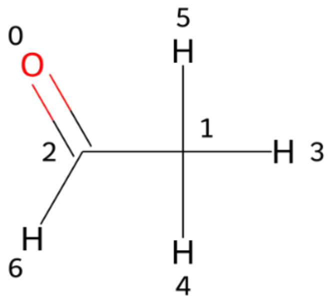	50.18	300	1: 30.89<br>2: 199.93	3,4,5: 2.206<br>6: 9.789	[3]
49	InChI=1S/CH2O2/c2-1-3/h1H,(H,2,3)	formic acid	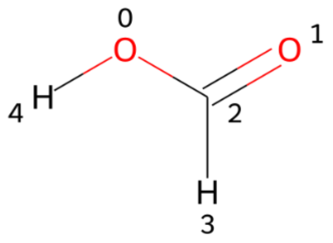	15.09	89.56	2: 166.22	3: 8.06<br>4: 10.99	[3]
50	InChI=1S/CH6N2/c1-3-2/h3H,2H2,1H3	methyl hydrazine	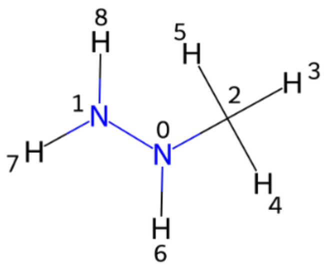	25.16	89.56	2: 43.49	3,4,5: 2.624<br>6,7,8: 3.21	[3]
51	InChI=1S/C3H3N/c1-2-3-4/h2H,1H2	acrylonitrile	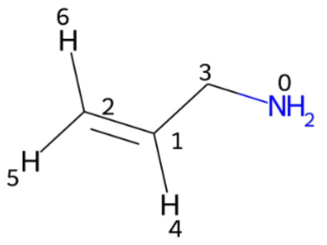	15.09	89.56	1: 107.81<br>2: 137.52<br>3: 117.13	4: 5.69<br>5: 6.24<br>6: 6.11	[3]
52	InChI=1S/C4H6/c1-3-4-2/h1-2H3	2-butyne	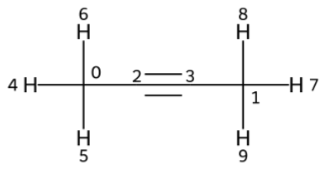	50.18	300	0,1: 3.33<br>2,3: 74.56	4,5,6,7,8,9: 1.74	[3]
53	InChI=1S/C3H4O/c1-2-3-4/h1,4H,3H2	propargyl alcohol	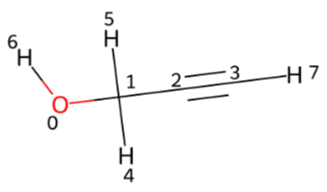	25.16	89.56	1: 50.40<br>2: 82.18<br>3: 73.88	4,5: 4.274<br>6: 2.37<br>7: 2.481	[3]
54	InChI=1S/C3H7N/c1-3-2-4-3/h3-4H,2H2,1H3	2methyl aziridine	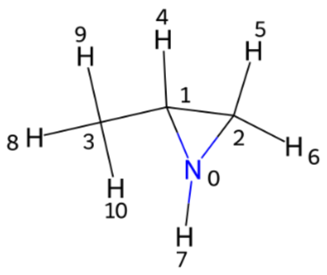	25.16	89.56	1: 25.23<br>2: 26.0<br>3: 19.54	4: 1.98<br>5: 1.30<br>6: 1.75<br>7: 0.24<br>8,9,10: 1.204	[3]

55	InChI=1S/C2H5NO/c1-3-2-4/h2H,1H3,(H,3,4)	N-methyl formamide	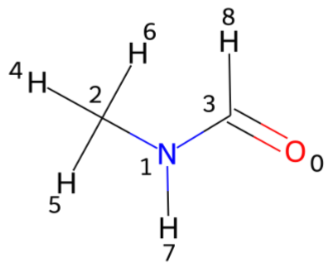	15.09	399.65	2: 28.21<br>3: 166.49	4,5,6: 2.815<br>7: 7.4<br>8: 8.159	3
56	InChI=1S/C3H9N/c1-3(2)4/h3H,4H2,1-2H3	isopropylamine	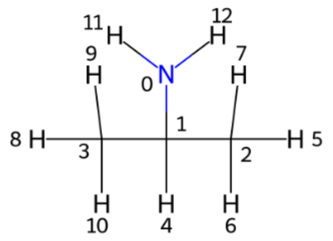	15.09	89.56	1: 42.83<br>2,3: 26.21	4: 3.102<br>5,6,7,8,9,10: 1.071<br>11,12: 1.221	3
57	InChI=1S/C3H8O/c1-2-3-4/h4H,2-3H2,1H3	propanol	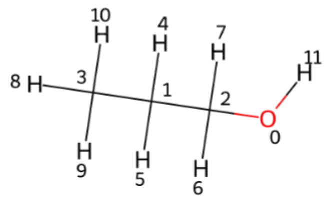	15.09	89.56	1: 25.89<br>2: 64.25<br>3: 10.28	4,5: 1.57<br>6,7: 3.582<br>8,9,10: 0.94<br>11: 2.26	3
58	InChI=1S/CH4NO2/c1-2(3)4/h1H3,(H,3,4)/q+1	nitromethane	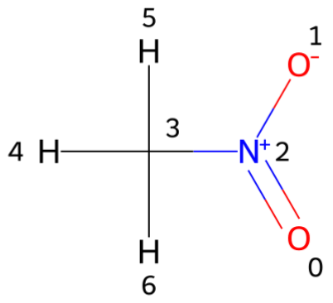	15.09	399.65	3: 62.59	4,5,6: 4.341	3
59	InChI=1S/C2H7NO/c3-1-2-4/h4H,1-3H2	ethanolamine	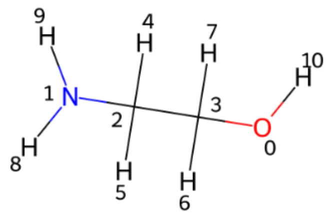	15.09	89.56	2: 43.96<br>3: 63.18	4,5: 2.78<br>6,7: 3.577<br>8,9: 2.76	3
60	InChI=1S/C3H2N2/c4-2-1-3-5/h1H2	malononitrie	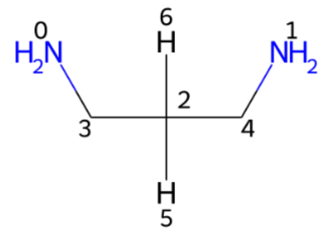	25.16	300	2: 8.77<br>3,4: 109.35	5,6: 3.604	3
61	InChI=1S/C3H4N2/c1-2-5-3-4-1/h1-3H,(H,4,5)	imidazole	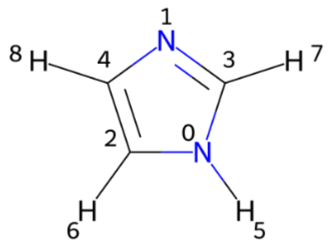	15.09	89.56	2,4: 121.88<br>3: 135.35	6,8: 7.129<br>7: 7.729	3
62	InChI=1S/C5H8/c1-3-5-4-2/h3-4H,1-2,5H2	1,4-pentadiene	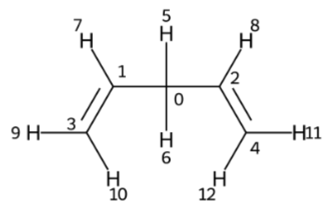	25.16	300	0: 38.01<br>1,2: 136.46<br>3,4: 115.47	5,6: 2.723<br>7,8: 5.71<br>9,12: 4.920<br>10,11: 4.95	3
63	InChI=1S/C5H8/c1-2-4-5-3-1/h1-2H,3-5H2	cyclopentene	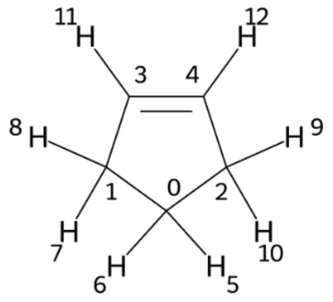	25.16	89.56	0: 22.98<br>1,2: 32.59<br>3,4: 130.77	5,6: 1.82<br>11,12: 5.73<br>7,8,9,10: 2.30	3

64	InChI=1S/C4H7N/c1-4(2)3-5/h4H,1-2H3	isobutyronitrile	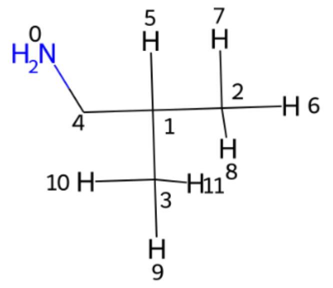	25.16	399.65	1: 19.87<br>2,3: 19.98<br>4: 123.83	5: 2.721<br>6,7,8,9,10,11: 1.331	[3]
65	InChI=1S/C4H6O/c1-2-3-4-5/h1,5H,3-4H2	3-butyn-1-OL	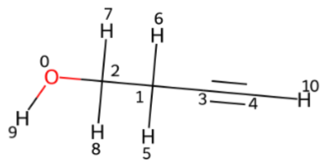	25.16	399.65	1: 22.68<br>2: 60.79<br>3: 81.40<br>4: 70.11	5,6: 2.449<br>7,8: 3.725<br>9: 3.48<br>10: 2.077	[3]
66	InChI=1S/C4H6O/c1-2-3-4-5/h2-4H,1H3/b3-2+	crotonaldehyde	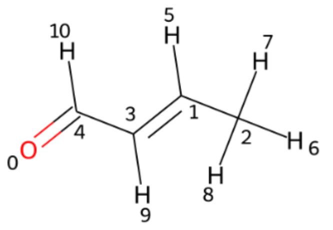	15.09	89.65	1: 154.32<br>2: 18.61<br>3: 134.61<br>4: 194.04	5: 6.88<br>6,7,8: 2.032<br>9: 6.145<br>10: 9.497	[3]
67	InChI=1S/C4H6O/c1-3-4(2)5/h1,4-5H,2H3	3-Butyn-2-ol	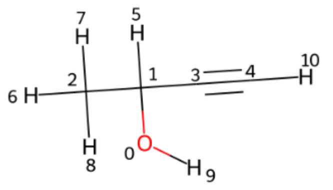	25.16	89.56	1: 57.86<br>2: 24.16<br>3: 86.03<br>4: 72.12	5: 4.533<br>6,7,8: 1.472<br>9: 3.0<br>10: 2.469	[3]
68	InChI=1S/C4H6O/c1-2-4-5-3-1/h1-2H,3-4H2	2,5-dihydrofuran	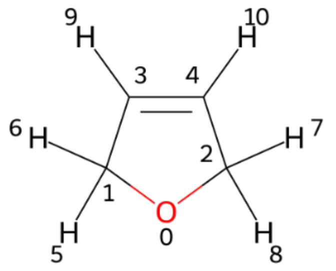	25.16	89.56	1,2: 75.42<br>3,4: 126.34	5,6,7,8: 4.636<br>9,10: 5.885	[3]
69	InChI=1S/C4H6O/c1-2-4-5-3-1/h1,3H,2,4H2	2,3-dihydrofuran	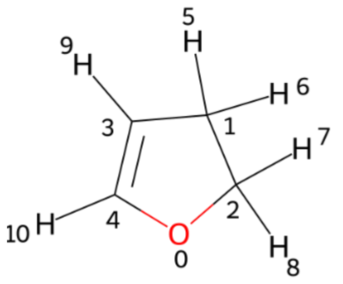	25.16	89.56	1: 29.28<br>2: 69.58<br>3: 99.54<br>4: 145.99	5,6: 2.61<br>7,8: 4.3<br>9: 4.951<br>10: 6.321	[3]
70	InChI=1S/C4H6O/c1-3-4(2)5/h3H,1H2,2H3	methyl vinyl ketone	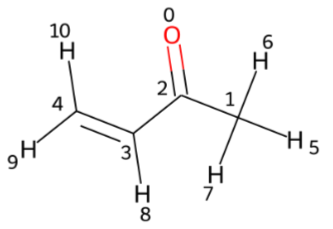	15.09	89.56	1: 26.32<br>2: 198.82<br>3: 137.49<br>4: 128.96	5,6,7: 2.286<br>8: 6.298<br>9: 5.908<br>10: 6.211	[3]
71	InChI=1S/C4H6O/c1-3-5-4-2/h1H,4H2,2H3	ethoxyethane	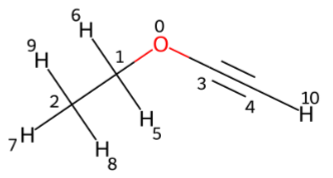	15.09	300	1: 74.61<br>2: 14.22<br>3: 90.85<br>4: 26.51	5,6: 4.125<br>7,8,9: 1.388<br>10: 1.530	[3]
72	InChI=1S/C5H10/c1-2-4-5-3-1/h1-5H2	cyclopentane	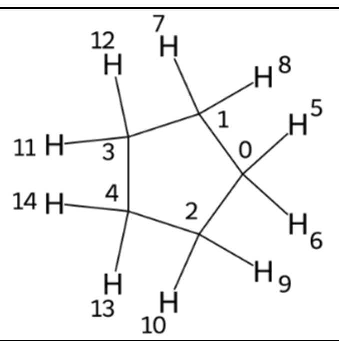	15.09	300	0,1,2,3,4: 26.05	5,6,7,8,9,10,11,12,13,14: 1.51	[3]

73	InChI=1S/C5H10/c1-4-5(2)3/h2,4H2,1,3H3	2-methyl-1-butene	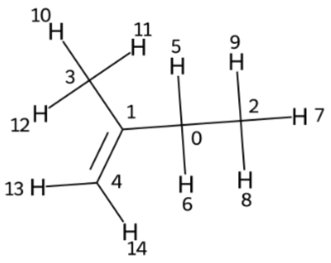	25.16	89.56	0: 30.75<br>1: 147.70<br>2: 12.41<br>3: 22.44<br>4: 108.57	5,6: 2.015<br>7,8,9: 1.031<br>10,11,12: 1.728<br>13,14: 4.67	[3]
74	InChI=1S/C5H10/c1-3-5-4-2/h3H,1,4-5H2,2H3	1-pentene	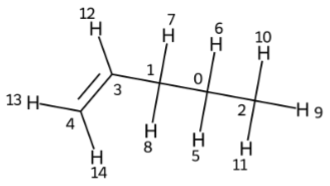	25.16	89.56	0: 22.26<br>1: 36.09<br>2: 13.66<br>3: 139.0<br>4: 114.39	5,6: 1.43<br>7,8: 2.02<br>9,10,11: 0.91<br>12: 5.81<br>13: 4.93<br>14: 4.97	[3]
75	InChI=1S/C5H10/c1-3-5-4-2/h3,5H,4H2,1-2H3/b5-3-	cis-2-pentene	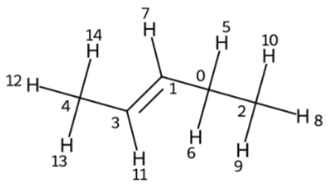	25.16	399.65	0: 20.25<br>1: 132.57<br>2: 14.16<br>3: 123.13<br>4: 12.59	5,6: 2.05<br>7: 5.38<br>8,9,10: 0.96<br>11: 5.40<br>12,13,14: 1.599	[3]
76	InChI=1S/C5H10/c1-3-5-4-2/h3,5H,4H2,1-2H3/b5-3+	trans-2-pentene	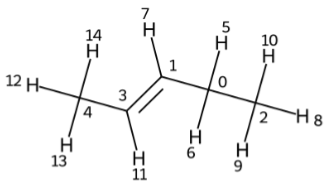	25.16	399.65	0: 25.80<br>1: 133.35<br>2: 14.02<br>3: 123.75<br>4: 17.90	5,6: 1.985<br>7: 5.45<br>8,9,10: 0.958<br>11: 5.41<br>12,13,14: 1.637	[3]
77	InChI=1S/C5H10/c1-4-5(2)3/h4H,1-3H3	2-methyl-2-butene	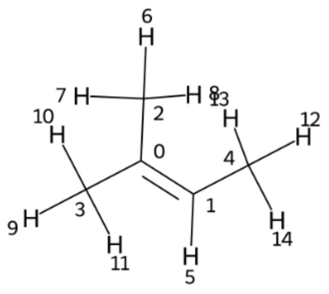	25.16	89.56	0: 132.08<br>1: 118.65<br>2: 25.67<br>3: 17.31<br>4: 13.43	5: 5.19<br>6,7,8: 1.60<br>9,10,11: 1.68<br>12,13,14: 1.56	[3]
78	InChI=1S/C3H5NO/c1-5-3-2-4/h3H2,1H3	methoxy acetonitrile	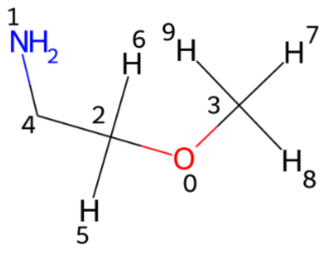	15.09	300	2: 59.08<br>3: 57.95<br>4: 116.22	5,6: 4.208<br>7,8,9: 3.477	[3]
79	InChI=1S/C5H12O/c1-2-3-4-5-6/h6H,2-5H2,1H3	1-pentanol	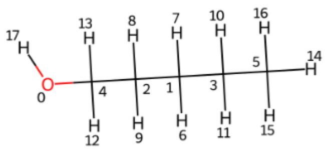	15.09	399.65	1: 28.17<br>2: 32.53<br>3: 22.66<br>4: 62.68<br>5: 14.09	6,7: 1.34<br>8,9: 1.558<br>10,11: 1.32<br>12,13: 3.596<br>14,15,16: 0.91<br>17: 3.05	[3]
80	InChI=1S/C3H5NO/c4-2-1-3-5/h5H,1,3H2	3-hydroxy propionitrile	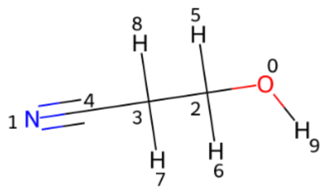	25.16	89.56	2: 57.43<br>3: 21.36<br>4: 118.89	5,6: 3.849<br>7,8: 2.612<br>9: 3.44	[3]
81	InChI=1S/C3H4O2/c1-2-3(4)5/h2H,1H2,(H,4,5)	acrylic acid	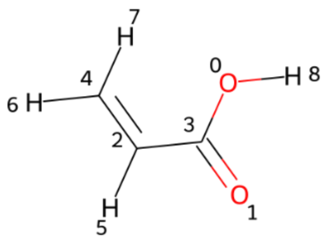	25.16	89.56	2: 128.14<br>3: 171.95<br>4: 133.15	5: 6.14<br>6: 5.96<br>7: 6.52<br>8: 12.0	[3]

82	InChI=1S/C4H8O/c1-3-4(2)5/h3H2,1-2H3	2-butanone	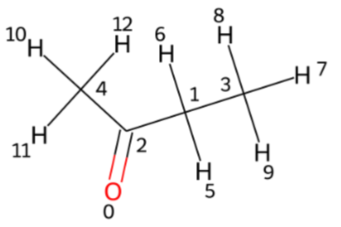	15.09	89.56	1: 36.87<br>2: 209.28<br>3: 7.87<br>4: 29.43	5,6: 2.449<br>7,8,9: 1.058<br>10,11,12: 2.139	[3]
83	InChI=1S/C4H8O/c1-2-4-3-5-4/h4H,2-3H2,1H3	1,2-epoxy butane	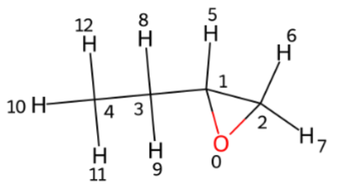	25.16	89.56	1: 53.40<br>2: 46.75<br>3: 25.59<br>4: 9.76	5: 2.887<br>6: 2.733<br>7: 2.475<br>8,9: 1.569<br>10,11,12: 1.012	[3]
84	InChI=1S/C4H8O/c1-2-3-4-5/h4H,2-3H2,1H3	butyraldehyde	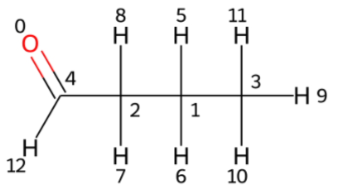	25.16	89.56	1: 15.69<br>2: 45.82<br>3: 13.71<br>4: 202.8	5,6: 1.64<br>7,8: 2.37<br>9,10,11: 0.97<br>12: 9.764	[3]
85	InChI=1S/C4H8O/c1-4(2)3-5/h3-4H,1-2H3	iso butyraldehyde	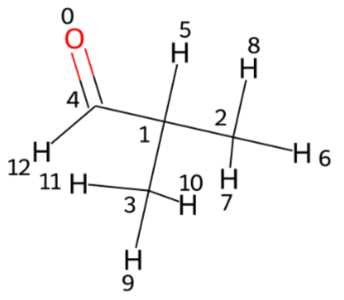	25.16		1: 41.07<br>2,3: 15.48<br>4: 204.86	5: 2.39<br>6,7,8,9,10,11: 1.06<br>12: 9.57	[3]
86	InChI=1S/C4H8O/c1-4(2)3-5/h5H,1,3H2,2H3	methallyl alcohol	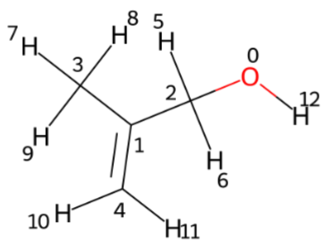	25.16	399.65	1: 145.05<br>2: 66.52<br>3: 19.14<br>4: 109.81	5,6: 3.991<br>7,8,9: 1.725<br>10: 4.832<br>11: 4.960<br>12: 3.35	[3]
87	InChI=1S/C4H8O/c1-3-5-4-2/h3H,1,4H2,2H3	ethyl vinyl ether	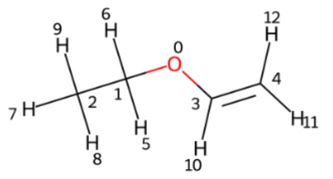	25.16	300	1: 63.63<br>2: 14.57<br>3: 151.93<br>4: 86.37	5,6: 3.74<br>7,8,9: 1.27<br>10: 6.46<br>11: 3.96<br>12: 4.17	[3]
88	InChI=1S/C4H8O/c1-2-3-4-5/h2,5H,1,3-4H2	3-Buten-1-ol	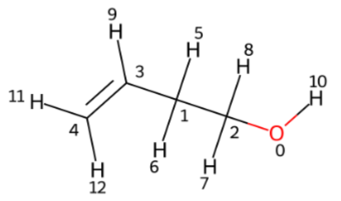	25.16	399.65	1: 37.11<br>2: 61.61<br>3: 135.02<br>4: 117.21	5,6: 2.318<br>7,8: 3.654<br>9: 5.81<br>10: 2.76<br>11: 5.1<br>12: 5.13	[3]
89	InChI=1S/C4H8O/c1-4(2)5-3/h1H2,2-3H3	2-methoxy propene	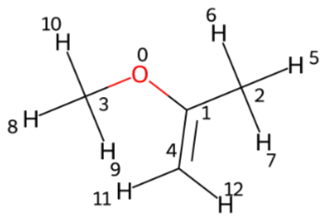	25.16	89.56	1: 160.89<br>2: 20.79<br>3: 54.7<br>4: 80.73	5,6,7: 1.82<br>8,9,10: 3.532<br>11,12: 3.85	[3]
90	InChI=1S/C5H12/c1-4-5(2)3/h5H,4H2,1-3H3	isopentane	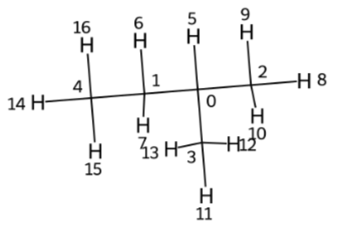	15.09	89.56	0: 30.02<br>1: 31.87<br>2: 11.83<br>3: 22.32	5: 1.447<br>6,7: 1.2<br>8,9,10: 0.869<br>14,15,16: 0.865	[3]

91	InChI=1S/C2H3NS/c1-4-2-3/h1H3	methyl thiocyanate	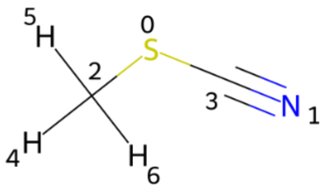	25.16	300	2: 16.39<br>3: 113.30	4,5,6: 2.596	[3]
92	InChI=1S/C3H7NO/c1-4(2)3-5/h3H,1-2H3	N,N-dimethyl formamide	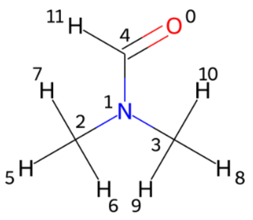	25.16	89.56	2: 36.43<br>3: 31.30<br>4: 162.6	5,6,7: 2.97<br>8,9,10: 2.883<br>11: 8.019	[3]
93	InChI=1S/C3H7NO/c1-3(2)4-5/h5H,1-2H3	acetone oxime	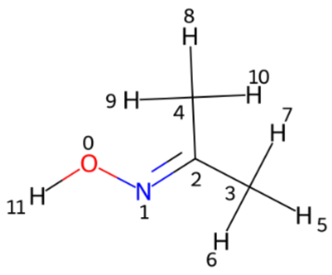	25.16	89.56	2: 155.51<br>3: 21.67<br>4: 15.0	5,6,7: 1.89<br>8,9,10: 1.9<br>11: 9.3	[3]
94	InChI=1S/C3H7NO/c1-2-3(4)5/h2H2,1H3,(H2,4,5)	propionamide	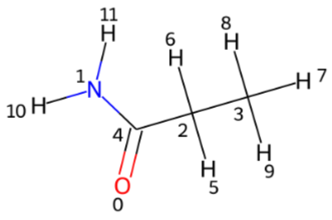	25.16	89.56	2: 28.95<br>3: 9.76<br>4: 177.68	5,6: 2.243<br>7,8,9: 1.153<br>10,11: 6.2	[3]
95	InChI=1S/C3H7NO/c1-2-4-3-5/h3H,2H2,1H3,(H,4,5)	N-ethyl formamide	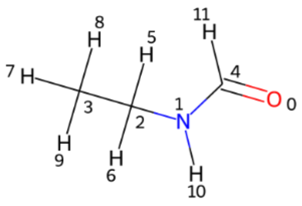	25.16	89.56	2: 36.87<br>3: 16.77<br>4: 164.92	5,6: 3.32<br>7,8,9: 1.173<br>10: 6.6<br>11: 8.13	[3]
96	InChI=1S/C4H11N/c1-3-4(2)5/h4H,3,5H2,1-2H3	sec-butylamine	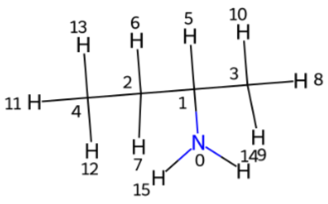	25.16	89.56	1: 48.55<br>2: 32.95<br>3: 23.50<br>4: 10.68	5: 2.795<br>6,7: 1.36<br>8,9,10: 1.053<br>11,12,13: 0.92<br>14,15: 1.25	[3]
97	InChI=1S/C4H11N/c1-4(2,3)5/h5H2,1-3H3	tert-butylamine	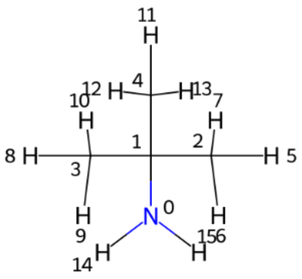	15.09	300	1: 47.39<br>2,3,4: 32.58	5,6,7,8,9,10,11,12,13: 1.149<br>14,15: 1.41	[3]
98	InChI=1S/C4H11N/c1-4-5(2)3/h4H2,1-3H3	N,N-dimethylamine	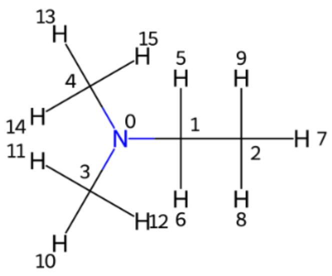	25.16	399.65	1: 53.63<br>2: 12.97<br>3,4: 45.11	5,6: 2.317<br>7,8,9: 1.064<br>10,11,12,13,14,15: 2.219	[3]
99	InChI=1S/C2H6N2O/c1-4(2)3-5/h1-2H3	N-nitroso dimethylamine	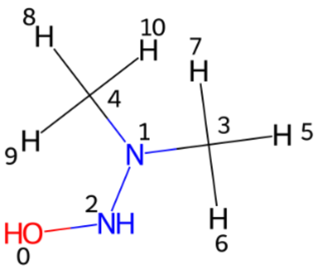	25.16	300	3: 32.75<br>4: 40.55	5,6,7: 3.804<br>8,9,10: 3.077	[3]
100	InChI=1S/C2H6N2O/c1-2(5)4-3/h3H2,1H3,(H,4,5)	acethydrazide	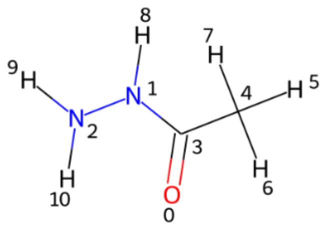	25.16	300	3: 171.23<br>4: 20.8	5,6,7: 1.976<br>8: 7.95<br>9,10: 3.97	[3]

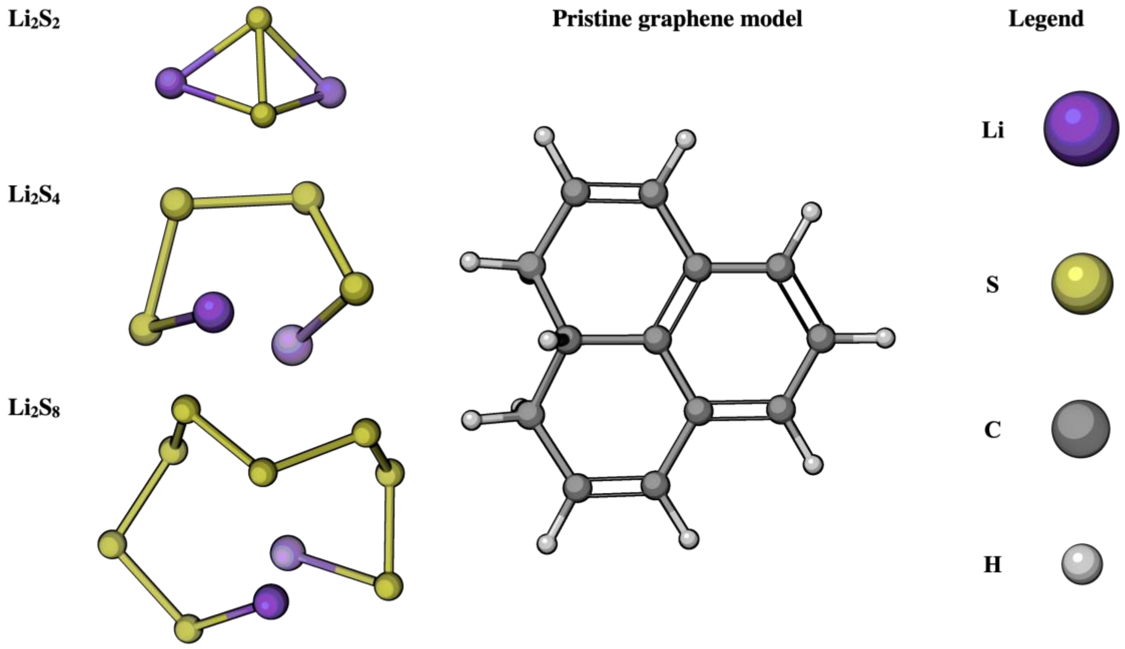

Fig. S8 Structure of Li_2S_x (x = 2, 4, 8) species and pristine graphene model used in the BE calculations

Table S6 Benchmarking results of the adiabatic IP/EA calculations using the SMD solvation model; potentials are reported relative to the Li^+/Li potential scale

	Experimental IP (V)	B3LYP/6-311++G**		B3LYP/def2-TZVPD	
		**IP (eV)**	**EA (eV)**	**IP (eV)**	**EA (eV)**
**6 – ethyl methyl sulfone**					
THF (ε = 7.4257)		6.191	-0.860	6.191	0.242
Acetone (ε = 20.493)	5.5 (LiTFSI)[4]	5.964	-0.776	5.947	0.527
Water (ε = 78.355)		6.072	-0.690	6.030	0.770
**27 – propylene carbonate**					
THF (ε = 7.4257)	5.8 ($LiClO_4$)[5]	6.904	-0.858	6.828	-0.734
Acetone (ε = 20.493)	> 6.0 ($LiPF_6$)[5]	6.697	-0.260	6.627	-0.493
Water (ε = 78.355)		6.564	0.303	6.482	0.091
**53 – ethoxy difluoro ethoxy ethane**					
THF (ε = 7.4257)		5.214	-1.031	5.213	0.175
Acetone (ε = 20.493)		5.025		5.002	0.507
Water (ε = 78.355)		4.995	-0.952	4.910	0.701

References

1 *Internet Bond-energy Databank (pKa and BDE)—iBonD Home Page*, <http://ibond.nankai.edu.cn/> (2022).

2 Fulmer, G. R. *et al.* NMR chemical shifts of trace impurities: common laboratory solvents, organics, and gases in deuterated solvents relevant to the organometallic chemist. *Organometallics* **29**, 2176-2179 (2010).

3 *SDBSWeb*, <https://sdbs.db.aist.go.jp> (2022).

4 Sun, X.-G. & Angell, C. A. New sulfone electrolytes for rechargeable lithium batteries.: Part I. Oligoether-containing sulfones. *Electrochemistry communications* **7**, 261-266 (2005).

5 Yamaki, J.-i. in *Advances in Lithium-Ion Batteries* 155-183 (Springer, 2002).
